# Headache in ADHD as comorbidity and a side effect of medications: a systematic review and meta-analysis

**DOI:** 10.1017/S0033291721004141

**Published:** 2022-01

**Authors:** Pei-Yin Pan, Ulf Jonsson, Sabriye Selin Şahpazoğlu Çakmak, Alexander Häge, Sarah Hohmann, Hjalmar Nobel Norrman, Jan K. Buitelaar, Tobias Banaschewski, Samuele Cortese, David Coghill, Sven Bölte

**Affiliations:** 1 Center of Neurodevelopmental Disorders (KIND), Centre for Psychiatry Research; Department of Women's and Children's Health, Karolinska Institutet & Stockholm Health Care Services, Region Stockholm, Stockholm, Sweden; 2 Child and Adolescent Psychiatry, Stockholm Health Care Services, Region Stockholm, Stockholm, Sweden; 3 Department of Neuroscience, Child and Adolescent Psychiatry, Uppsala University, Uppsala, Sweden; 4 Department of Child and Adolescent Psychiatry and Psychotherapy, Medical Faculty Mannheim, Central Institute of Mental Health, University of Heidelberg, Mannheim, Germany; 5 Department of Cognitive Neuroscience, Donders Institute for Brain, Cognition and Behaviour, Radboud University Nijmegen Medical Centre, and Karakter Child and Adolescent Psychiatry University Centre, Nijmegen, Netherlands; 6 Faculty of Environmental and Life sciences & Clinical and Experimental Sciences (CNS and Psychiatry), Faculty of Medicine, Centre for Innovation in Mental Health, School of Psychology, University of Southampton, Southampton, UK; 7 Solent NHS Trust, Southampton, UK; 8 Division of Psychiatry and Applied Psychology, School of Medicine, University of Nottingham, Nottingham, UK; 9 New York University Child Study Center, New York, NY, USA; 10 Departments of Paediatrics and Psychiatry, Faculty of Medicine, Dentistry and Health Sciences, University of Melbourne, and Murdoch Children's Research Institute, Melbourne, VIC, Australia; 11 Curtin Autism Research Group, School of Occupational Therapy, Social Work and Speech Pathology, Curtin University, Perth, WA, Australia

**Keywords:** ADHD medication, attention-deficit/hyperactivity disorder, headache, meta-analysis, systematic review

## Abstract

There is mixed evidence on the association between headache and attention-deficit/hyperactivity disorder (ADHD), as well as headache and ADHD medications. This systematic review and meta-analysis investigated the co-occurrence of headache in children with ADHD, and the effects of ADHD medications on headache. Embase, Medline and PsycInfo were searched for population-based and clinical studies comparing the prevalence of headache in ADHD and controls through January 26, 2021. In addition, we updated the search of a previous systematic review and network meta-analysis of double-blind randomized controlled trials (RCTs) on ADHD medications on June 16, 2020. Trials of amphetamines, atomoxetine, bupropion, clonidine, guanfacine, methylphenidate, and modafinil with a placebo arm and reporting data on headache as an adverse event, were included. Thirteen epidemiological studies and 58 clinical trials were eligible for inclusion. In epidemiological studies, a significant association between headache and ADHD was found [odds ratio (OR) = 2.01, 95% confidence interval (CI) = 1.63–2.46], which remained significant when limited to studies reporting ORs adjusted for possible confounders. The pooled prevalence of headaches in children with ADHD was 26.6%. In RCTs, three ADHD medications were associated with increased headache during treatment periods, compared to placebo: atomoxetine (OR = 1.29, 95% CI = 1.06–1.56), guanfacine (OR = 1.43, 95% CI = 1.12–1.82), and methylphenidate (OR = 1.33, 95% CI = 1.09–1.63). The summarized evidence suggests that headache is common in children with ADHD, both as part of the clinical presentation as such and as a side effect of some standard medications. Monitoring and clinical management strategies of headache in ADHD, in general, and during pharmacological treatment are recommended.

## Introduction

Headache in childhood is common and disabling. The estimated prevalence during 1 month to lifetime is 58.4%; migraine affects 7.7% (Abu-Arafeh, Razak, Sivaraman, & Graham, 2010). Pediatric headaches have been linked to sleep problems (Dosi et al., 2013; Esposito, Parisi, Miano, & Carotenuto, 2013), emotional dysfunction (Arruda & Bigal, 2012*a*; Fuh et al., 2010), impaired peer relations (Karwautz et al., 1999), and poor academic performance (Arruda & Bigal, 2012*b*; Genizi et al., 2013). Its clinical management is challenging due to the multifactorial origin and co-existing medical and psychiatric conditions (Bellini et al., 2013; Guidetti, Faedda, & Siniatchkin, 2016; Whitehouse & Agrawal, 2017).

Attention-deficit/hyperactivity disorder (ADHD) is a neurodevelopmental condition characterized by impairing inattention, hyperactivity and impulsivity, with a worldwide prevalence of 5–7% in children and adolescents (Polanczyk, Willcutt, Salum, Kieling, & Rohde, 2014; Thomas, Sanders, Doust, Beller, & Glasziou, 2015). Youths with ADHD have been reported to frequently suffer from comorbid headaches (Akmatov, Ermakova, & Batzing, 2021; Kutuk et al., 2018). Headache can not only have a negative effect on general well-being, but it may also aggravate cognitive problems defining or being associated with ADHD, such as inattention and impaired information processing (Moutran et al., 2011; Riva et al., 2006; Waldie, Hausmann, Milne, & Poulton, 2002). As a common side effect during pharmacological treatment of ADHD (Clavenna & Bonati, 2017), the headache might also decrease compliance to medications (Ahmed & Aslani, 2013) and increase rates of treatment failure (Buitelaar et al., 2015), with negative long-term consequences for outcomes (Barkley, 2008).

Therefore, a better understanding of the association of headache and ADHD, both as comorbidity and a side effect of medication, might help optimizing clinical management. However, although an extensive body of epidemiological research and clinical trials of ADHD medications exist, the current knowledge of the occurrence of headaches in children and adolescents with ADHD remains limited in several ways. First, estimated odds ratios (ORs) for headache in ADHD *v.* non-ADHD populations (typically developing children and those with neurodevelopmental disorders other than ADHD) vary across studies. Therefore, pooled results from a meta-analysis could provide a more robust and precise point estimate with a higher statistical power than the information derived from individual studies. Second, previous systematic reviews investigated the risk of ADHD in individuals with headaches, not vice versa (Salem et al., 2018). Thus, aggregated data focusing on headaches in pediatric ADHD as the target population are not available. Third, reviews on the association between ADHD medications and headache in children included randomized control trials (RCTs) regardless of blindness (Cheng, Chen, Ko, & Ng, 2007; Punja et al., 2016; Ruggiero et al., 2014; Storebø et al., 2015; Wang et al., 2017), which could introduce bias. In addition, there is a lack of information on possible differences between ADHD medications.

Thus, we conducted a systematic review and meta-analysis to examine two research questions (RQ): (1) Does the prevalence of headache differ between children with and without ADHD? (2) Is the use of common medications for ADHD associated with headaches in children?

## Methods

This systematic review was conducted in accordance with the PRISMA statement (Liberati et al., 2009). The protocol was preregistered with PROSPERO (CRD42020176574).

**Review question 1:** Does the prevalence of headache differ between children and adolescents with and without ADHD?

### Eligibility criteria

Case-control and cross-sectional/longitudinal studies which recruited either community or clinical samples with a control group comparing the odds of having headaches between children and adolescents diagnosed with ADHD and controls without ADHD were eligible. The population of interest was children and adolescents (aged ⩽18 years) with a diagnosis of ADHD according to the Diagnostic and Statistical Manual of Mental Disorders (DSM) (-III, -III-R, -IV, -IV-TR or 5) or hyperkinetic disorder (HKD) in the International Classification of Diseases and Related Health Problems (ICD) (−8, −9, −10), ascertained by either clinical assessment or validated instruments, or a positive answer to the question to parents: “Did a doctor/healthcare professional ever tell you that your child has ADHD?” The indicators for headache were either (1) A positive answer to the question to participants/parents: “Do you have (Have you ever had) headache/migraine?” “Does your child have (Has your child ever had) headache/migraine?”; or (2) a diagnosis of primary headache (migraine, tension-type headache, cluster headache, and other types) recorded in medical files/registries, or made by clinicians in a research setting. OR was the principal summary measure when comparing headaches in children and adolescents with ADHD *v.* the comparison group.

### Search strategy

A systematic search was conducted with the assistance of two information specialists from the Karolinska Institutet University Library using the databases Embase, Medline and PsycInfo from their inception to January 26, 2021. This search was limited to studies published in English and in peer-reviewed journals. The complete search strategy is provided in eSearch strategy in the Supplement.

### Study selection and coding

Two reviewers independently screened the abstracts of the retrieved articles for eligibility, and then assessed the full text of potentially relevant publications. A manual search for additional studies was performed by screening reference lists of identified pertinent review articles. Disagreements between reviewers were resolved by consensus. When no consensus could be reached, a third reviewer acted as arbitrator. Data extraction was conducted using a standardized form by two independent reviewers. Extracted information included: study type, study settings, diagnostic tools for ADHD and headache, number and demographics characteristics of participants, and information for risk of bias assessment.

### Risk of bias assessment

The risk of bias was assessed independently by two reviewers using the Newcastle–Ottawa scale (NOS) (Wells et al., 2009). Methodological quality including the risk of misclassification and selection bias, comparability between cases and controls, and appropriate statistical analysis was appraised and rated in eight items. Any discrepancies in the scoring were resolved by discussion and reviewer consensus.

**Review question 2:** Is the use of common medications for ADHD associated with headaches in children and adolescents?

### Eligibility criteria

We included double-blind RCTs in either parallel or crossover design with a placebo arm and treatment period for at least 7 consecutive days. Trials without adequate randomization (defined as in Cortese et al., 2018) or washout period were excluded. The study population was children and adolescents (aged ⩾5 years and ⩽18 years) with a diagnosis of ADHD by either DSM (III, III-R, IV, IV-TR or 5) or of HKD according to the ICD (−8, −9, −10). While Cortese et al. (2018), due to the need of adhering to the transitivity assumption, included only medications as monotherapy and in tablet/capsule forms, here we allowed the intervention as ADHD medications (monotherapy or add-on to non-pharmacological treatment) in any formulation (tablet, capsule, chewable compound, liquid, transdermal) and dosing strategy, including (1) methylphenidate (including dexmethylphenidate), (2) amphetamines (including lisdexamfetamine), and (3) non-stimulants (atomoxetine, bupropion, clonidine, guanfacine, and modafinil). The comparator was the placebo arm. The outcome of interest was a headache in the safety assessment, obtained by either spontaneously report, open-ended questioning (e.g. do you have any physical discomforts?), or questionnaires/checklists for side effects. Studies published in peer-reviewed journals or available data uploaded on websites of protocol registration for clinical trials were considered.

### Search strategy

We based this part of the review on a search performed for a systematic review and network meta-analysis of ADHD medications by Cortese et al. (2018), which, according to the AMSTAR 2 tool (Shea et al., 2017), we deemed to be of high quality, as all 16 items of the AMSTAR 2, including those related to the search, were scored as “Yes.” The search included a broad set of databases [PubMed, BIOSIS Previews, CINAHL, Cochrane Library, EMBASE, ERIC, MEDLINE, PsycINFO, OpenGrey, Web of Science Core Collection, ProQuest Dissertations & Theses: UK & Ireland, ProQuest Dissertations & Theses A&I, and WHO International Trials Registry Platform (CTRP) (including ClinicalTrials.gov)]. In addition, they had searched for unpublished studies or data sets by contacting drug companies and study authors. The search for the network meta-analysis by Cortese et al. (2018). is updated once per year, and the search performed on 16 June 2020 was used for the present review.

### Study selection and coding

All articles receiving full-text assessment in the previous review (the full list of included and excluded studies with exclusion reasons from Cortese et al., 2018) and the additional articles found in the updated search were screened independently by two reviewers using the inclusion and exclusion criteria of the present review. Disagreements were resolved by discussion between the two review authors. Data extraction was also performed independently for the following information: publication detail, trial registration identification number, characteristics of trial participants, study design, interventions, methods of outcome measurement, and information for risk of bias assessment.

### Risk of bias assessment

The revised Cochrane risk of bias tool for randomized trials (RoB 2.0) (Sterne et al., 2019) was used for assessing bias arising from the following five domains: randomization process, deviations from intended interventions, missing outcome data, measurement of the outcome, and selection of the reported result. The overall risk-of-bias judgment was rated as low risk of bias, some concerns, and high risk of bias, which was the lowest rating for any of the criteria (e.g. if any domain was scored high risk of bias, the study was considered high risk of bias). The appraisal was performed independently by two reviews and disagreements were solved by consensus.

### Planned methods of analysis

The metafor package (Viechtbauer, 2010) in R version 3.2.4. (RStudio Team, 2015) was used for computing pooled estimates and 95% confidence intervals (CIs). A random-effects model was adopted in data syntheses considering the sample and methodological diversity among the included epidemiological studies and clinical trials. For RQ1, the ORs of headache in children and adolescents with ADHD *v.* the comparison group were synthesized. In addition, a pooled prevalence estimate (the proportion of headaches in children with ADHD) was calculated. Freeman–Tukey double arcsine transformations (Freeman & Tukey, 1950) were used to stabilize the variance, and the harmonic mean of individual sample sizes (Miller, 1978) was used in the back-transformation. For RQ2, we calculated the pooled OR comparing the risk of headache among participants in the treatment arm with those in the placebo arm for each specific ADHD medication (data of safety analysis were used). The heterogeneity for each meta-analysis was estimated with the *Q* and *I*^2^ statistics (Higgins & Thompson, 2002). The significance of publication bias was tested using Egger's weighted regression and estimated visually based on funnel plots. Subgroup analysis based on potential covariates (e.g. maximal dosage, dosing strategy) was used to address significant heterogeneity of outcome measures across studies.

## Results

### Review question 1

#### Study selection and characteristics

**Fig. 1.**
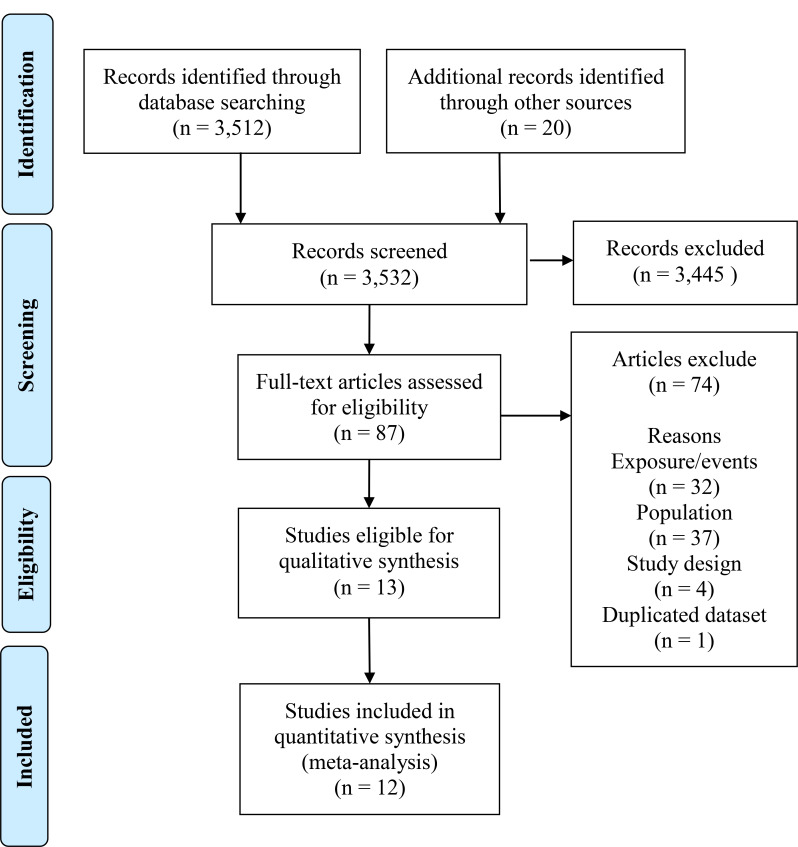
PRISMA flow chart of review process and study selection for epidemiological studies.

A total of 13 records were identified for inclusion in the review (eIncluded articles 1 in the Supplement). See study selection process in Fig. 1 and reasons for exclusion of references for which the full text was assessed in eExclusion reasons 1 in the Supplement. Characteristics of included articles are summarized in Table 1A and eTable 1 in the Supplement. The total sample included 267 556 children and adolescents with ADHD, identified mainly by validated instruments (*n* = 6). The comparison group comprised of 2 464 878 participants, all recruited from the community.

**Table 1.** Summary of characteristics of the included studies

A. Review question 1. Characteristics of epidemiological studies (*n* = 13)		
Country (*n*)		Brazil (2), Canada (1), Germany (1), Sweden (2), Turkey (1), USA (6)	
Type of study (*n*)		Case-control (3), Cross-sectional (10)	
Study temporality (*n*)		All cross-sectional	
Settings of ADHD recruitment (*n*)		Clinical (2), Community (11)	
ADHD diagnostic assessment (*n*)		ICD-9 (1), ICD-10 (1), DSM-III (1), DSM-IV (2), DSM-5 (1), A-TAC (1), SNAP-IV (2), SDQ (3), CBCL (1), Conner-10 items (1), parent-report questionnaire (2)	
Type of headache (*n*)		Headache/migraine (8), Migraine (5), Tension Headache (3)	
Diagnostic indicator for headache (*n*)		Clinical diagnosis (1), medical records/registry (2), parent report (10), self-report (1)	
Survey period (*n*)		1 year (7), 4 years (1), lifetime (5)	
B. Review question 2. Characteristics of clinical trials (*n* = 58)		
Amphetamine (*n* = 10)	Country (*n*)	USA (7), Japan (1), sample from 10 European countries (1), sample from USA, Canada, and European countries (1)
	Study design (*n*)	All double-blind; all parallel design; all naturalistic setting; all monotherapy
	Treatment duration (*n*)	3w (2), 4w (5), 6w (1), 7w (1), 8w (1)
	Method for identifying headache (*n*)	Parent report questionnaire (1), spontaneously report (1), open-ended question (1), NR (7)
	Participants discontinued due to headache (*n*)	Reported cases (1), no cases (6), NR (3)
	Total participants in the safety analysis	Treatment group: 1780, placebo group: 892
	Pooled rate of headache during treatment	Treatment group: 14.8%, placebo group: 13.1%
Atomoxetine (*n* = 22)	Country (*n*)	USA (10), Germany (2), Australia (1), Italy (1), Japan (1), Russia (1), Spain (1), Sweden (1), Taiwan (1), the Netherlands (1), sample from Europe and Australia (1), sample from USA, Europe, and Canada (1)
	Study design (*n*)	All double-blind; parallel (21), crossover (1); all natralistic setting; all monotherapy
	Treatment duration (*n*)	6w (6), 8w (7), 9w (2), 10w (2), 12w (3), 13w (1), 18w (1)
	Method for identifying headache (*n*)	Parent report questionnaire (1), Spontaneously report (2), open-ended question (12), NR (7)
	Other remarks (*n*)	All participants stimulant-naïve (2), all participants comorbid with ASD (1), all participants comorbid with an anxiety disorder (1), all participants comorbid with ODD or CD (3), all participants comorbid with MDD (1), all participants comorbid with tics (1)
	Participants discontinued due to headache (*n*)	Reported cases (4), no cases (11), NR (7)
	Total participants in the safety analysis	Treatment group: 2387, placebo group: 1470
	Pooled rate of headache during treatment	Treatment group: 16.3%, placebo group: 12.7%
Bupropion (*n* = 1)	Country	USA
	Study design	Quadruple blind, parallel design; natralistic setting; add-on to CBT
	Treatment duration	16w
	Method for identifying headache	NR
	Other remarks	all participants comorbid with SUD
	Participants discontinued due to headache	NR
	Total participants in the safety analysis	Treatment group: 53, placebo group: 52
	Rate of headache during treatment	Treatment group: 22.6%, placebo group: 26.9%
Clonidine (*n* = 1)	Country	USA
	Study design	Double-blind, parallel design; natralistic setting; monotherapy
	Treatment duration	16w
	Method for identifying headache	Spontaneously report
	Participants discontinued due to headache	0
	Total participants in the safety analysis	Treatment group: 31, placebo group: 30
	Rate of headache during treatment	Treatment group: 16.1%, placebo group: 10.0%
Guanfacine (*n* = 8)	Country (*n*)	USA (6), sample from USA and Canada (1), sample from USA, Canada and Europe (1)
	Study design (*n*)	All double-blind; all parallel design; natralistic setting (7), laboratory classroom study (1); all monotherapy
	Treatment duration (*n*)	5w (1), 6w (1), 8w (2), 9w (2), 13w (2)
	Method for identifying headache (*n*)	Spontaneously report (1), NR (7)
	Participants discontinued due to headache (*n*)	Reported cases (2), no cases (3), NR (3)
	Total participants in the safety analysis	Treatment group: 1275, placebo group: 681
	Pooled rate of headache during treatment	Treatment group: 23.0%, placebo group: 17.3%
Methylphenidate (*n* = 17)	Country (*n*)	USA (15), 10 European countries (1), sample from USA, Canada, Germany, Hungary, Sweden (1)
	Study design (*n*)	All double blind; parallel (15), crossover (2); natralistic setting (16), classroom setting (1); monotherapy (16), add-on to CBT (1)
	Route (*n*)	Oral (16), transdermal (1)
	Drug release profile (*n*)	Immediate-release (3), extended-release (7), OROS (6)
	Treatment duration (*n*)	1w (2), 3w(3), 4w(3), 5w (1), 6w (2), 7w (3), 8w (1), 12w (1), 16w(1)
	Method for identifying headache (*n*)	Spontaneously report (5), parent report questionnaire (3), open-ended question (1), NR (9)
	Other remarks (*n*)	All participants females (1), All participants comorbid with SUD (1), using both parent-report questionnaires and spontaneously report by participants to identify headache (1)
	Participants discontinued due to headache (*n*)	Reported cases (2), no cases (10), NR (5)
	Total participants in the safety analysis	Treatment group: 2077, placebo group: 1294
	Pooled rate of headache during treatment	Treatment group: 16.6%, placebo group: 12.5%
Modafinil (*n* = 6)	Country (*n*)	USA (5), Iran (1)
	Study design (*n*)	All double-blind; all parallel design; all natralistic setting; all monotherapy
	Treatment duration (*n*)	4w (1), 6w (2), 7w (1), 9w (2)
	Method for identifying headache (*n*)	Spontaneously report (3), parent report questionnaire (1), physician-administered questionnaire (1), NR (1)
	Participants discontinued due to headache (*n*)	Reported cases (2), no cases (2), NR (2)
	Total participants in the safety analysis	Treatment group: 520, placebo group: 298
	Pooled rate of headache during treatment	Treatment group: 16.7%, placebo group: 14.3%

ICD, International Classification of Diseases and Related Health Problems; DSM, Diagnostic and Statistical Manual of Mental Disorders; A-TAC, Autism-Tics, ADHD and other Comorbidities; SNAP-IV, Swanson, Nolan, and Pelham Rating Scale; SDQ, Strengths and Difficulties Questionnaire; CBCL, Child Behavior Checklist; NR, not reported; ASD, autism spectrum disorder; ODD, oppositional defiant disorder; CD, conduct disorder; MDD, major depressive disorder; SUD, substance use disorder; CBT, cognitive behavior therapy.

#### Results of individual studies and syntheses of results

**Fig. 2.**
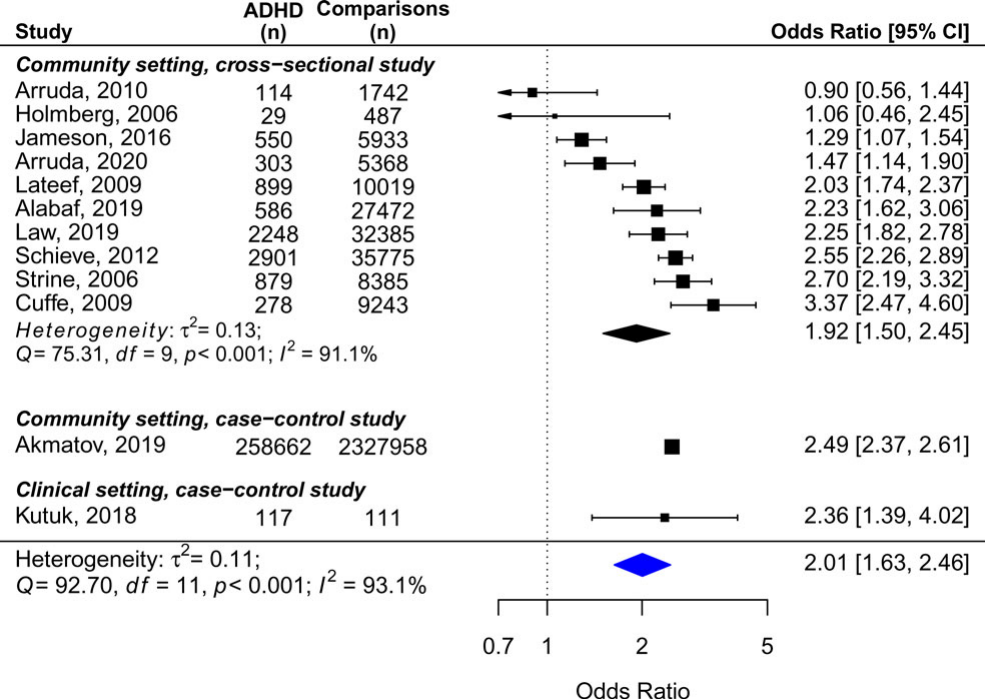
Unadjusted ORs expressing the association between headache and ADHD in children and adolescents.

Figure 2 displays the crude ORs of the included studies and the pooled estimates (2.01, 95% CI = 1.63 − 2.46, *n* = 12), as well as the results of subgroup analysis for the community studies with cross-sectional design. One small outlier study with an OR of 14.79 (Kaplan, McNicol, Conte, & Moghadam, 1987), which had recruited only preschool children in clinical settings, was excluded from the meta-analysis, owing to susceptibility of selection bias. In addition, the sample size of Akmatov et al. (2021) was much larger compared to other studies. We decided to include this article for the similar OR with other studies, and the high study quality according to the risk of bias assessment. Results were heterogeneous across studies when considering all types of headache, but most studies (*n* = 10) suggested higher rates of headache in ADHD than in non-ADHD controls (range of crude OR 0.90–3.37). Regarding the relationships between diagnostic assessments and ORs, three studies used clinical assessment for both ADHD and headache (Akmatov et al., 2021; Kutuk et al., 2018; Law, Palermo, Zhou, & Groenewald, 2019), and the ORs were 2.49, 2.36, and 2.25. Two studies used a single question to identify both ADHD and headache (Lateef et al., 2009; Schieve et al., 2012), and the ORs were 2.03 and 2.55. Other studies which used mixed diagnostic indicators reported ORs ranging from 0.90 to 3.37. Due to the heterogeneity of findings (*Q* = 92.70, *p* < 0.001; *I*^2^ = 93.1%), we also conducted subgroup analysis for the studies with adjusted OR, the studies measuring migraine, and those measuring tension headache (Table 2). The synthesized results indicated that studies that controlled for age, sex, race, and other socioeconomic status variables found a significant association between ADHD and overall headache [*n* = 4, pooled OR (95% CI) = 1.98 (1.60–2.45)]. However, heterogeneity was still high among the studies measuring migraine (*Q* = 10.38, *p* = 0.035; *I*^2^ = 67.6%), and no difference in the risk of tension headache was found between ADHD and controls. The pooled prevalence of overall headache in children with ADHD over a period of 1 year to lifetime is shown in eFigure 1 [*n* = 11; one study did not provide the data of prevalence; pooled proportion (95% CI) = 26.6 (14.2–41.3) %], with significant heterogeneity detected.

**Table 2.** Summary of results of pooled ORs about the association between ADHD and headache in the main and in the subgroup meta-analyses

Type of analysis	Studies (*n*)	Participants (*n*)		OR	95% CI	*p*	Heterogeneity				Egger's test	
							*τ*^2^	*Q*	*p*	*I*^2^ (%)	*t*	*p*
**Review question 1**		ADHD	Control									
Overall crude OR	12	267 556	2 464 878	2.01	1.63–2.46	<0.001	0.11	92.70	<0.001	93.1	−1.79	0.104
Adjusted OR^a^	4	5229	60 112	1.98	1.60–2.45	<0.001	0.33	7.81	0.050	64.7	−0.31	0.789
Migraine	5	259 782	2 362 651	2.22	1.76–2.79	<0.001	0.04	10.38	0.035	67.6	−0.94	0.418
Tension headache	3	534	7221	0.80	0.57–1.12	0.196	0.03	3.38	0.185	36.5	1.07	0.478
**Review question 2**		Treatment	Placebo									
Amphetamine	10	1780	892	1.05	0.80–1.38	0.723	0.04	11.26	0.258	21.53	2.10	0.069
Atomoxetine	22	2387	1470	1.29	1.06–1.56	0.010	0	18.90	0.528	0	1.27	0.221
Maximum dosage < 1.5 mg/kg	9	902	620	1.53	1.01–2.30	0.043	0.11	12.76	0.240	28.7	1.37	0.214
Maximum dosage ⩾ 1.5 mg/kg	13	1485	850	1.22	0.96–1.54	0.105	0	7.88	0.724	0	0.16	0.878
Forced titration	11	1368	708	1.33	1.01–1.75	0.041	0.02	13.26	0.209	8.46	−0.11	0.918
Flexible/optimized dose	11	1019	762	1.24	0.93–1.65	0.148	0	5.51	0.788	0	2.66	0.029
Bupropion	1	53	52	0.79	0.33–1.93	0.613						
Clonidine	1	31	30	1.73	0.38–7.99	0.482						
Guanfacine	8	1275	681	1.43	1.12–1.82	0.005	0	4.17	0.760	0	2.05	0.086
Methylphenidate	17	2077	1294	1.33	1.09–1.63	0.005	0	10.96	0.812	0	−0.58	0.570
Monotherapy with oral formation	15	1781	1070	1.41	1.12–1.78	0.003	0	9.81	0.776	0	−1.22	0.245
OROS/extended release	13	1793	1132	1.37	1.11–1.70	0.003	0	7.74	0.805	0	0.57	0.578
Immediate release	3	139	90	1.00	0.33–3.00	0.999	0.28	2.38	0.305	27.3	−2.64	0.231
Modafinil	6	520	298	1.24	0.73–2.13	0.429	0.18	7.61	0.179	42.3	0.59	0.589

OROS, osmotic-release oral system.

a Adjusted variables: Jameson et al. (2016), adjusted for age, sex, race, and parental education; Lateef et al. (2009), adjusted for sex, age, race, and poverty index ratio; Schieve et al. (2012), adjusted for age, sex, race, and maternal education; Strine, Okoro, McGuire, & Balluz. (2006), adjusted for age, sex, race, parental structure, poverty status, type of health care coverage, and number of comorbid conditions.

#### Risk of bias across studies

Data on the risk of bias of each study are presented in eTable 1 in the Supplement. The most frequent sources of risk of bias were inadequate ascertainment of ADHD and headache, as well as the lack of non-response rate in both ADHD and non-ADHD groups. Regarding the comparability of cases and controls, most studies were cross-sectional studies without control groups matched for demographic characteristics. The heterogeneity across studies became non-significant in the adjusted subgroup analysis. This underscores the need for appropriate statistical methods to address the influence of possible confounders on the estimates. In addition, most studies did not include information on the treatment status of ADHD, which might influence the prevalence of headaches.

The results of Egger's test indicated no evidence of publication bias (*t* = − 1.79, *p* = 0.104). Nevertheless, some estimates lying on the top of the funnel plot but outside the triangular region suggest sample selection variance across studies (eFigure 2 in the Supplement), which could arise from the diversity of methods for identifying participants with ADHD.

### Review question 2

#### Study selection and characteristics

The update search by Cortese et al. (2018) retrieved 194 additional RCTs. A total of 58 trials met the eligibility criteria for the present systematic review. See all the related articles of the included trials and the exclusion reasons in eIncluded articles 2 and eExclusion reasons 2 in the Supplement.

The main characteristics of the included trials are displayed in Table 1B and eTable 2 in the Supplement. The eligible trials involved a total of 8254 participants who were allocated to the treatment groups and included in the safety analysis, and 4087 participants in the control groups assessed in the same way.

#### Risk of bias across studies

The majority of included trials (*n* = 41, 70.7%) were rated as overall low risk of bias regarding the outcome of headache, 16 trials (27.6%) with some concerns, and 1 trial (1.7%) as high risk of bias (eTable 2 in the Supplement).

#### Results of individual studies, syntheses of results, and publication bias

**Fig. 3.**
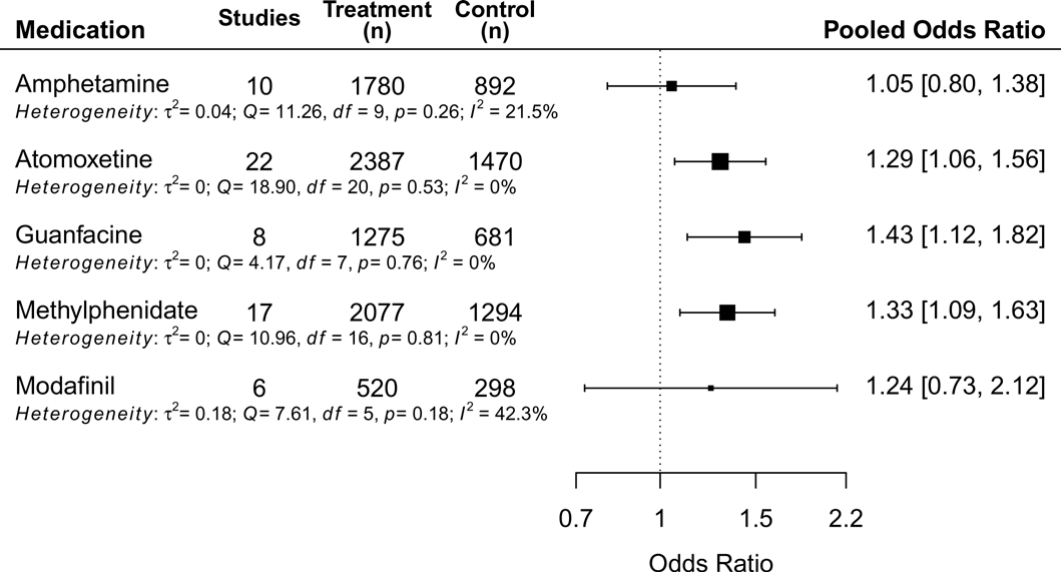
Forest plot of risk of headache during treatment periods compared with placebo as reference.

Except for bupropion and clonidine (one eligible study for each was identified by our search), the pooled OR on the risk of headache between treatment and control groups for five different ADHD medications are summarized in Fig. 3. The forest plots and funnel plots of each medication are presented in the eFigure 3–13 in the Supplement. The pooled prevalence of headache for the treatment and placebo groups across studies for each medication ranged from 14.8% (amphetamine) to 23.0% (guanfacine) in the treatment groups, and from 12.2% (methylphenidate) to 17.3% (guanfacine) in the control groups (Table 1B, eFigure 14–15). eFigure 16 displays the pooled prevalence of headache across different age groups in placebo arms (children 12.6%, adolescents 16.5%). The results of subgroup analyses are presented in Table 2. The results for each active medication (in alphabetic order) are detailed below.

#### Amphetamine

None of the included nine trials reported a significant difference in risk of headache between treatment and control arms [eFigure 3, pooled OR (95% CI): 1.05 (0.80 − 1.83)]. No evidence of heterogeneity was found in the meta-analysis, although the CI is wide compared to the synthesized results of other ADHD medications.

#### Atomoxetine

Among the total 22 trials (21 datasets), one trial reported a higher risk of headache in participants treated with atomoxetine, while others showed no difference between treatment and control groups. The synthesized results indicated a significant association between atomoxetine and headache in children with ADHD [pooled OR (95% CI): 1.29 (1.06 − 1.56)]. Two types of subgroup analyses were performed. The effects of dosage showed paradoxical findings, in which the association remained significant in trials with smaller dosage (*p* = 0.043), but did not survive in those with larger (maximum dosage cutoff value 1.5 mg/kg/day, Table 2 and eFigure 5). However, forced titration was more commonly used in trials with smaller dosages (55.6% *v.* 41.7%), and the association was also found in trials using forced titration (*p* = 0.041), but not in those using flexible/optimized dose (Table 2 and eFigure 6). Therefore, it should be considered that the paradoxical effect of dosage could be driven by the dosing strategy. Despite that the sample in this meta-analysis included participants with a diverse range of comorbidities, all estimates except one fitted within the triangular region of the funnel plot, indicating small effects of sampling variation on the pooled estimate.

#### Bupropion

One trial comparing the effectiveness of bupropion add-on to CBT in the treatment of adolescents with comorbid ADHD and substance use disorders was included. No significant difference in the risk of headache during the intervention period was found between the treatment and placebo group [OR (95% CI): 0.79 (0.33 − 1.93)].

#### Clonidine

The only included trial investigated the efficacy and safety profiles of clonidine and methylphenidate among children with ADHD. There was no significant difference in the risk of headache between the group treated with clonidine and placebo [OR (95% CI): 1.73 (0.38 − 7.99)].

#### Guanfacine

None of the eight trials in the meta-analysis reported a difference in the risk of headache between treatment and placebo groups. However, the pooled estimates indicated that the odds of headache in the treatment group were 1.43 times higher than in the placebo group [pooled OR (95% CI): 1.43 (1.12 − 1.82)].

#### Methylphenidate

Seventeen trials were included in the meta-analysis. None of them found a significant association between methylphenidate treatment and headache. Nevertheless, an increased risk of headache with methylphenidate was indicated by the pooled estimates [pooled OR (95% CI): 1.33 (1.09 − 1.63)], and also in the subgroup analysis on trials using an oral formation with monotherapy design [*n* = 15, pooled OR (95% CI): 1.41 (1.12 − 1.78)]. When considering the duration of drug action, long-acting formation [the osmotic-release oral system (OROS) and extended release] was significantly associated with a higher risk of headache with the treatment of methylphenidate compared to controls (Table 2). The pooled estimate of the three trials with immediate-release methylphenidate showed no significant difference between treatment and controls, but the CI was wide [pooled OR (95% CI): 1.00 (0.33–3.00)].

#### Modafinil

Of the six trials included in this review, none reported a significant difference in risk of headache between treatment and placebo arms. The results of the meta-analysis did not find a significant association between modafinil and headache [pooled OR (95% CI): 1.24 (0.73 − 2.13)]. However, the CI was wide due to the relatively small sample size.

## Discussion

This is the first systematic review and meta-analysis on the association between headache and ADHD, and the effects of ADHD medications in childhood. Results indicate that in pediatric ADHD there is a doubled risk compared to those without ADHD to have a headache, with a pooled prevalence of 26.6%. Despite the heterogeneity across studies in the meta-analysis of crude data, the association survived when pooling ORs adjusted for possible confounders, including age, sex, and socioeconomic status (Bigal & Lipton, 2006). Moreover, in short-term RCTs of ADHD medications, increased risks of headache were found for atomoxetine, guanfacine, and methylphenidate treatments compared to placebos. However, no statistically significant associations of headache with amphetamine, bupropion, clonidine, and modafinil were found; yet, the precision of these estimates was limited by the number of studies, sample sizes, and methods for measuring headache. In summary, at this stage, it is premature to draw conclusions about the difference between medication in its effects on headaches.

The effect size of the pooled estimate for atomoxetine was relatively small, and there was an effect of dosing strategy but not dose-related effects on headache. However, the finding may reflect a long-term tolerability profile of atomoxetine, in which headache is the most frequent treatment-emergent adverse event, reported by more than 50% of pediatric patients with 3–4 years treatment duration (Donnelly et al., 2009). For guanfacine, headache has been found to be amongst the most common reasons for discontinuation, although the most common adverse effects reported are sedation and somnolence, in part explained by its affinity to *α*2A adrenergic receptors (Bello, 2015). Methylphenidate is the most widely prescribed ADHD medication among children and adolescents owing to its overall efficacy and safety (Cortese et al., 2018), but has also been found to be associated with headache in a previous meta-analysis of 37 trials (Storebø et al., 2015). Moreover, long-acting methylphenidate (OROS/extended-release), which is thought with the advantage of better adherence *v.* immediate-release formulation (Adler & Nierenberg, 2010), was also associated with a headache when analyzed separately.

Most trials included in this systematic review were underpowered to detect side effects. This makes it difficult to draw conclusions from single trials, especially since headache naturally co-occurs with ADHD. The majority of included trials did not report an increased risk of headache. Still, a significant risk was observed for several agents when looking at the totality of trials. This might explain why headache, despite being among the most common treatment-related adverse events of ADHD medication (Clavenna & Bonati, 2017), has received limited attention in previous research and clinical practice guidelines, compared to sleep disturbance, growth delay, loss of appetite, and cardiovascular risks (Cortese et al., 2013; Graham et al., 2011). Our results highlight that clinicians should be aware of the risk of headache during treatment with ADHD medications, in order to improve adherence and avoid unnecessary harm.

Headache phenotypes in pediatric ADHD have not been discussed in detail in the literature previously. Our results suggest an increased risk of migraine in children with ADHD, but not a risk of tension-type headache, in line with the findings from a previous meta-analysis (Salem et al., 2018). Nevertheless, the classification of childhood headaches is still debated. The main reason is that headache phenotypes often change over time (Brna, Dooley, Gordon, & Dewan, 2005), and different headache diagnoses may represent a continuum rather than discrete entities (Turner et al., 2015). Postulated pathophysiological links between ADHD and headache include malfunction of the dopaminergic system and brain iron deficiency, as well as shared genetic pathways (Kutuk et al., 2018; Parisi et al., 2014). In addition, the association between ADHD and headache could be mediated by internalizing symptoms and sleep disorders, where the common dysfunction of sleep–wake and arousal system might lead to alteration of the pain processing (Guidetti et al., 2016; Pan & Yeh, 2017; Parisi et al., 2014). Unfortunately, there is currently no evidence-based guidance available regarding headache management in children with ADHD, neither regarding the effects of prophylaxis of migraine using valproate, amitriptyline and flunarizine on ADHD symptoms (Villa, Agessi, Moutran, Gabbai, & Carvalho Dde, 2016), nor for strategies of medications hierarchies for those with ADHD who also suffer from headache.

Several gaps in the literature were identified. For example, no eligible longitudinal studies aiming to disentangle the temporal relationship between the emerging of attention problems and headache symptoms were identified. In addition, only a limited number of studies recruited clinical samples of children with ADHD, which might experience headache profiles different from community samples. Further, most of the included epidemiological studies did not obtain the participants' history of pharmacological treatment. Finally, studies were not adjusted for other risk factors of headache, such as family history, medical conditions, and psychiatric comorbidity (Bellini et al., 2013; Lateef & Merikangas, 2017).

The results of this systematic review should be considered in the context of several limitations. First, the heterogeneity of outcomes across the included epidemiological studies diminishes the confidence of pooled ORs. This could partly reflect a bias of misclassification introduced by the inconsistent indicators used for identifying ADHD. Therefore, our confidence in the pooled proportion of headaches in ADHD children is restricted. Second, the majority of the included clinical trials did not detect treatment-related headaches in a standardized way, such as the Side Effects Rating Scale for stimulants (Barkley & Murphy, 2005). On the contrary, a physical examination, which always includes an open-ended question “do you have any physical discomforts?”, was the most commonly adopted method in the trials to obtain information. However, the sensitivity to headache identification by one open-ended item might be limited, compared to a well-designed instrument that collects the elicited responses from the participants in a consistent manner (Takemura et al., 2005). In addition, specific diagnoses of headaches were generally not provided. Third, the summary measure of headache used in clinical trials is the cumulative incidence rate. Since headache was detected at different visits during the study period, we were unable to analyze the relationship between time of exposure to drug and risk of headache for each medication. Fourth, we could not include studies lacking relevant data, and the prerequisite of data availability could have introduced publication bias. Fifth, the study samples in clinical trials could be highly selected. In addition to the inclusion and exclusion criteria, only voluntary participants who could comply with the protocol and had rather a good family support would be eligible to enter the trials. Furthermore, there is no data available on children with subclinical ADHD variants, who are often also in need of support and some sort of clinical action (Kirova et al., 2019). Thus, the external validity of the results for both ADHD diagnosis and symptoms of ADHD should be considered due to the possible overall limited representativeness of the samples.

In conclusion, findings show that children and adolescents with ADHD are more likely to experience headaches than their peers. Clinicians should be aware that headache is a common comorbidity of pediatric ADHD, but also a frequent side effect of pharmacological treatments. To enable practitioners to provide better care for children with comorbid ADHD and headaches, clinical guidelines supported by empirical evidence are desirable.

## Supplementary material

For supplementary material accompanying this paper visit https://doi.org/10.1017/S0033291721004141. click here to view supplementary material
