# Supplementary material for: Headache in ADHD as comorbidity and a side effect of medications: a systematic review and meta-analysis
Source: Psychol Med. 2021 Oct 12;52(1):14–25. doi: 10.1017/S0033291721004141 (PMC8711104; doi:10.1017/S0033291721004141)

**Supplementary Content**

**eSearch strategy.** Search strategy for review question 1.

**eIncluded articles 1.** Studies/citations retained for review question 1 in the present systematic review

**eExclusion reasons 1.** Studies/citations discarded after assessing their full text for review question 1, with reasons for exclusions.

**eTable 1.** Summary of characteristics of included case-control/cross-sectional studies (*n* = 13)

**eFigure 1.** Caterpillar plot of pooled prevalence of overall headache in children with ADHD

**eFigure 2.** Funnel plot of epidemiological studies on the association between ADHD and headache among children

**eIncluded articles 2.** Studies/citations retained for review question 2 in the present meta-analysis

**eExclusion reasons 2.** Studies/citations discarded after assessing their full text for review question 2, with reasons for exclusions.

**eTable 2.** Summary of characteristics of included clinical trials (*n* = 58)

**eFigure 3.** Forest plot of risk of headache between amphetamine treatment groups and placebo groups

**eFigure 4.** Funnel plot of trials on the association between amphetamine and headache among children with ADHD

**eFigure 5.** Forest plot of risk of headache between atomoxetine treatment groups and placebo groups, with subgroup analysis based on maximum dosage

**eFigure 6.** Forest plot of risk of headache between atomoxetine treatment groups and placebo groups, with subgroup analysis based on dosing strategy

**eFigure 7.** Funnel plot of trials on the association between atomoxetine and headache among children with ADHD

**eFigure 8.** Forest plot of risk of headache between guanfacine treatment groups and placebo groups

**eFigure 9.** Funnel plot of trials on the association between guanfacine and headache among children with ADHD

**eFigure 10.** Forest plot of risk of headache between methylphenidate treatment groups and placebo groups

**eFigure 11.** Funnel plot of trials on the association between methylphenidate and headache among children with ADHD

**eFigure 12.** Forest plot of risk of headache between modafinil treatment groups and placebo groups

**eFigure 13.** Funnel plot of trials on the association between modafinil and headache among children with ADHD

**eFigure 14.** Caterpillar plot of trials on the prevalence of headache among children with ADHD treated with ADHD medications

**eFigure 15.** Caterpillar plot of trials on the prevalence of headache among children with ADHD in placebo arms

**eFigure 16.** Caterpillar plot of trials on the prevalence of headache among youths with ADHD in placebo arms based on different age groups

**eSearch strategy.** Search strategy for review question 1.

Documentation of search strategies

University Library search consultation group

Date: January 26, 2021

Topic/research question: Headache in ADHD: comorbidity and effects of medication

Name of researcher(s): Pei-Yin Pan, Ulf Jonsson & Sven Bölte, KBH/Center of Neurodevelopmental Disorders at Karolinska Institutet (KIND)

Librarian(s): Magdalena Svanberg, Emma-Lotta Säätelä & Narcisa Hannerz

Databases:

1. Medline (Ovid)
2. Embase (embase.com)
3. PsycInfo (Ovid)

Total number of hits:

- Before deduplication: 4,350
- After deduplication: 3,512

Comments:

1. Medline

| Interface: Ovid MEDLINE(R) and Epub Ahead of Print, In-Process & Other Non-Indexed Citations and Daily  Date of Search: 26 January 2021  Number of hits: 641  Comment: In Ovid, two or more words are automatically searched as phrases; i.e. no quotation marks are needed | Field labels   - exp/ = exploded MeSH term - / = non exploded MeSH term - .ti,ab,kf. = title, abstract and author keywords - adjx = within x words, regardless of order - * = truncation of word for alternate endings |
| --- | --- |
| Database(s): **Ovid MEDLINE(R) and Epub Ahead of Print, In-Process & Other Non-Indexed Citations and Daily**1946 to January 25, 2021 Search Strategy:   \| **#** \| **Searches** \| **Results** \| \| --- \| --- \| --- \| \| 1 \| exp Attention Deficit Disorder with Hyperactivity/ \| 29522 \| \| 2 \| ((attention or hyperactiv* or inattention) adj3 (deficit* or disabilit* or disorder*)).ti,ab,kf. \| 35204 \| \| 3 \| (hyperactivity and (attention or inattention)).ti,ab,kf. \| 29719 \| \| 4 \| (addh or adhd or hyperkinetic*).ti,ab,kf. \| 30129 \| \| 5 \| or/1-4 \| 51180 \| \| 6 \| Headache/ \| 28194 \| \| 7 \| exp Headache disorders/ \| 35164 \| \| 8 \| (cephalalgia* or head ache* or headache* or migraine*).ti,ab,kf. \| 107342 \| \| 9 \| or/6-8 \| 118816 \| \| 10 \| 5 and 9 \| 641 \| | |

2. Embase

| Interface: embase.com  Date of Search: 26 January 2021  Number of hits: 3,402  Comment: Emtree is the controlled vocabulary in Embase | Field labels   - /exp = exploded Emtree term - /de = non exploded Emtree term - ti,ab = title and abstract - NEAR/x = within x words, regardless of order - * = truncation of word for alternate endings |
| --- | --- |
| Embase Session Results  \| **#1** \| **'attention deficit disorder'**/exp \| 64,750* \| \| --- \| --- \| --- \| \| **#2** \| ((**attention*** OR **hyperactiv*** OR **inattention***) NEAR/3 (**deficit*** OR **disabilit*** OR **disorder***)):ti,ab,kw \| 48,571* \| \| **#3** \| **hyperactivity**:ti,ab,kw AND (**inattention**:ti,ab,kw OR **attention**:ti,ab,kw) \| 38,524* \| \| **#4** \| **addh**:ti,ab,kw OR **adhd**:ti,ab,kw OR **hyperkinetic*** \| 43,929* \| \| **#5** \| #1 OR #2 OR #3 OR #4 \| 83,766* \| \| **#6** \| **'headache and facial pain'**/exp \| 320,522* \| \| **#7** \| **cephalalgia***:ti,ab,kw OR **'head ache*'**:ti,ab,kw OR **headache***:ti,ab,kw OR **migraine***:ti,ab,kw \| 168,666* \| \| **#8** \| #6 OR #7 \| 346,268* \| \| **#9** \| #5 AND #8 \| 3,402 \| | |

3. Psycinfo

| Interface: Ovid  Date of Search: 26 January 2021  Number of hits: 307  Comment: In Ovid, two or more words are automatically searched as phrases; i.e. no quotation marks are needed | Field labels   - exp/ = exploded controlled term - / = non exploded controlled term - .ti,ab,id. = title, abstract and author keywords - adjx = within x words, regardless of order - * = truncation of word for alternate endings |
| --- | --- |
| Database(s): **PsycINFO**1806 to January Week 4 2021 Search Strategy:   \| **#** \| **Searches** \| **Results** \| \| --- \| --- \| --- \| \| 1 \| exp attention deficit disorder/ \| 27923 \| \| 2 \| ((attention or hyperactiv* or inattention) adj3 (deficit* or disabilit* or disorder*)).ti,ab,id. \| 36533 \| \| 3 \| (hyperactivity and (attention or inattention)).ti,ab,id. \| 31150 \| \| 4 \| (addh or adhd or hyperkinetic*).ti,ab,id. \| 30390 \| \| 5 \| or/1-4 \| 44297 \| \| 6 \| exp headache/ \| 15480 \| \| 7 \| (cephalalgia* or head ache* or headache* or migraine*).ti,ab,id. \| 23606 \| \| 8 \| or/6-7 \| 23725 \| \| 9 \| 5 and 8 \| 307 \| | |

**eInclusion articles 1.** Studies/citations retained for review question 1 in the present systematic reivew

1. **Akmatov 2021**

Akmatov MK, Ermakova T, Bätzing J. Psychiatric and Nonpsychiatric Comorbidities Among Children With ADHD: An Exploratory Analysis of Nationwide Claims Data in Germany. *J Atten Disord*. 2021; 25(6), 874–884. doi: 10.1177/1087054719865779.

1. **Alabaf 2019**

Alabaf S, Gillberg C, Lundström S, et al. Physical health in children with neurodevelopmental disorders. *J Autism Dev Disord*. 2019;49(1):83-95. doi: 10.1007/s10803-018-3697-4.

1. **Arruda 2010**

Arruda MA, Guidetti V, Galli F, Albuquerque RC, Bigal ME. Migraine, tension-type headache, and attention-deficit/hyperactivity disorder in childhood: a population-based study. *Postgrad Med*. 2010;122(5):18-26. doi: 10.3810/pgm.2010.09.2197.

1. **Arruda 2020**

Arruda MA, Arruda R, Guidetti V, Bigal ME. ADHD Is Comorbid to Migraine in Childhood: A Population-Based Study. *J Atten Disord*. 2020;24(7):990-1001. doi: 10.1177/1087054717710767.

1. **Cuffe 2009**

Cuffe SP, Moore CG, McKeown R. ADHD and health services utilization in the national health interview survey. *J Atten Disord*. 2009;12(4):330-340. doi: 10.1177/1087054708323248.

1. **Holmberg 2006**

Holmberg K, Hjern A. Health complaints in children with attention-deficit/hyperactivity disorder. *Acta Paediatr*. 2006;95(6):664-670. doi: 10.1080/08035250600717121.

1. **Jameson 2016**

Jameson ND, Sheppard BK, Lateef TM, Vande Voort JL, He JP, Merikangas KR. Medical Comorbidity of Attention-Deficit/Hyperactivity Disorder in US Adolescents. *J Child Neurol*. 2016;31(11):1282-1289. doi: 10.1177/0883073816653782.

1. **Kaplan 1987**

Kaplan BJ, McNicol J, Conte RA, Moghadam HK. Physical signs and symptoms in preschool-age hyperactive and normal children. *J Dev Behav Pediatr*. 1987;8(6):305-310.

1. **Kutuk 2018**

Kutuk MO, Tufan AE, Guler G, et al. Migraine and associated comorbidities are three times more frequent in children with ADHD and their mothers. *Brain Dev*. 2018;40(10):857-864. doi: 10.1016/j.braindev.2018.06.001.

1. **Lateef 2009**

Lateef TM, Merikangas KR, He J, Kalaydjian A, Khoromi S, Knight E, Nelson KB. Headache in a national sample of American children: prevalence and comorbidity. *J Child Neurol*. 2009;24(5):536-543. doi: 10.1177/0883073808327831.

1. **Law 2019**

Law EF, Palermo TM, Zhou C, Groenewald CB. Economic Impact of Headache and Psychiatric Comorbidities on Healthcare Expenditures Among Children in the United States: A Retrospective Cross-Sectional Study. *Headache*. 2019;59(9):1504-1515. doi: 10.1111/head.13639.

1. **Schieve 2012**

Schieve LA, Gonzalez V, Boulet SL, et al. Concurrent medical conditions and health care use and needs among children with learning and behavioral developmental disabilities, National Health Interview Survey, 2006-2010. *Res Dev Disabil*. 2012;33(2):467-476. doi: 10.1016/j.ridd.2011.10.008.

1. **Strine 2006**

Strine TW, Okoro CA, McGuire LC, Balluz LS. The associations among childhood headaches, emotional and behavioral difficulties, and health care use. Pediatrics. 2006;117(5):1728-1735. doi: 10.1542/peds.2005-1024.

**eExclusion reasons 1.** Studies/citations discarded after assessing their full text for review question 1, with reasons for exclusions.

**Review question 1: Does the prevalence of headache differ between children and adolescents with and without ADHD?**

1. **Aggarwal 2020**

Aggarwal SS, Ott SD, Padhye NS, Schulz PE. Sex, race, ADHD, and prior concussions as predictors of concussion recovery in adolescents. *Brain Inj*. 2020;34(6):809-817. doi: 10.1080/02699052.2020.1740942.

*Reason for exclusion: Target population was not ADHD*

1. **Al-Haidar 2003**

Al-Haidar FA. Inpatient child and adolescent psychiatric referrals in Saudi Arabia: clinical profiles and treatment. *East Mediterr Health J.* 2003;9(5-6):996-1002.

*Reason for exclusion: Target population was not ADHD*

1. **Al-Sharbati 2003**

Al-Sharbati MM, Al-Hussaini AA, Antony SX. Profile of child and adolescent psychiatry in Oman. *Saudi Med J*. 2003;24(4):391-395.

*Reason for exclusion: Target population was not ADHD*

1. **Anttila 2004**

Anttila P, Sourander A, Metsähonkala L, Aromaa M, Helenius H, Sillanpää M. Psychiatric symptoms in children with primary headache. *J Am Acad Child Adolesc Psychiatry*. 2004;43(4):412-419. doi: 10.1097/00004583-200404000-00007.

*Reason for exclusion: No diagnosis of ADHD*

1. **Arruda 2014**

Arruda MA, Arruda R. Psychological adjustment in children with episodic migraine: a population-based study. *Psychol Neurosci*. 2014;7(1),33-41. doi: 10.3922/j.psns.2014.1.06

*Reason for exclusion: No diagnosis of ADHD; same dataset as Arruda 2017.*

1. **Arruda 2012**

Arruda MA, Bigal ME. Migraine and migraine subtypes in preadolescent children: association with school performance. *Neurology*. 2012;79(18):1881-1888. doi: 10.1212/WNL.0b013e318271f812.

*Reason for exclusion: No diagnosis of ADHD; same dataset as Arruda 2017.*

1. **Bai 2017**

Bai G, Herten MH, Landgraf JM, Korfage IJ, Raat H. Childhood chronic conditions and health-related quality of life: Findings from a large population-based study. *PLoS One*. 2017;12(6):e0178539. doi: 10.1371/journal.pone.0178539.

*Reason for exclusion: No specific question used to obtain the diagnosis of ADHD*

1. **Bansal 2011**

Bansal PD, Barman R. Psychopathology of school going children in the age group of 10-15 years. *Int J Appl Basic Med Res*. 2011;1(1):43-47. doi: 10.4103/2229-516X.81980.

*Reason for exclusion: No data of comorbidity of ADHD and headache provided*

1. **Battistutta 2009**

Battistutta S, Aliverti R, Montico M, Zin R, Carrozzi M. Chronic tension-type headache in adolescents. Clinical and psychological characteristics analyzed through self- and parent-report questionnaires. *J Pediatr Psychol*. 2009;34(7):697-706. doi: 10.1093/jpepsy/jsn102.

*Reason for exclusion: No diagnosis of ADHD*

1. **Beales 2012**

Beales DJ, Smith AJ, O'Sullivan PB, Straker LM. Low back pain and comorbidity clusters at 17 years of age: a cross-sectional examination of health-related quality of life and specific low back pain impacts. *J Adolesc Health*. 2012;50(5):509-516. doi: 10.1016/j.jadohealth.2011.09.017.

*Reason for exclusion: No diagnosis of ADHD*

1. **Blaauw 2014**

Blaauw BA, Dyb G, Hagen K, et al. Anxiety, depression and behavioral problems among adolescents with recurrent headache: the Young-HUNT study. *J Headache Pain*. 2014;15(1):38. doi: 10.1186/1129-2377-15-38.

*Reason for exclusion: No diagnosis of ADHD*

1. **Blackman 2011**

Blackman JA, Gurka MJ, Gurka KK, Oliver MN. Emotional, developmental and behavioural co-morbidities of children with chronic health conditions. *J Paediatr Child Health*. 2011;47(10):742-747. doi: 10.1111/j.1440-1754.2011.02044.x.

*Reason for exclusion: Target population was not ADHD; no comparison group*

1. **Bruijn 2009**

Bruijn J, Arts WF, Duivenvoorden H, Dijkstra N, Raat H, Passchier J. Quality of life in children with primary headache in a general hospital. *Cephalalgia*. 2009;29(6):624-630. doi: 10.1111/j.1468-2982.2008.01774.x.

*Reason for exclusion: No data of comorbidity of ADHD and headache provided*

1. **Bursztein 2006**

Bursztein C, Steinberg T, Sadeh A. Sleep, sleepiness, and behavior problems in children with headache. *J Child Neurol*. 2006;21(12):1012-1019. doi: 10.1177/7010.2006.00239.

*Reason for exclusion: No diagnosis of ADHD; target population was not ADHD; no comparison group*

1. **Carpenet 2019**

Carpenet C, Guichard E, Tzourio C, Kurth T. Self-perceived attention deficit and hyperactivity symptom levels and risk of non-migraine and migraine headaches among university students: A cross-sectional study. *Cephalalgia*. 2019;39(6):711-721. doi: 10.1177/0333102418804155.

*Reason for exclusion: Target population was adults*

1. **Carter-Pokras 2019**

Carter-Pokras OD, Bugbee BA, Gold RS, Lauver PE, Aiken R, Arria AM. Utilizing Student Health and Academic Data: A County-Level Demonstration Project. *Health Promot Pract*. Published online Aug 9, 2019. doi: 10.1177/1524839919862796.

*Reason for exclusion: No data of comorbidity of ADHD and headache provided*

1. **Cook 2020**

Cook NE, Sapigao RG, Silverberg ND, et al. Attention-Deficit/Hyperactivity Disorder Mimics the Post-concussion Syndrome in Adolescents. *Front Pediatr*. 2020;8:2. doi: 10.3389/fped.2020.00002.

*Reason for exclusion: No comparison group; ADHD was identified by self-report questionnaires*

1. **Del Bene 1982**

Del Bene E. Multiple aspects of headache risk in children. *Adv Neurol*. 1982;33:187-198.

*Reason for exclusion: No diagnosis of ADHD; target population was not ADHD; no comparison group*

1. **Egger 1998**

Egger HL, Angold A, Costello EJ. Headaches and psychopathology in children and adolescents. *J Am Acad Child Adolesc Psychiatry*. 1998;37(9):951-958. doi: 10.1097/00004583-199809000-00015.

*Reason for exclusion: No baseline data provided*

1. **Egger 1999**

Egger HL, Costello EJ, Erkanli A, Angold A. Somatic complaints and psychopathology in children and adolescents: stomach aches, musculoskeletal pains, and headaches*. J Am Acad Child Adolesc Psychiatry*. 1999;38(7):852-860. doi: 10.1097/00004583-199907000-00015.

*Reason for exclusion: No baseline data provided*

1. **Fasmer 2012**

Fasmer OB, Riise T, Lund A, Dilsaver SC, Hundal O, Oedegaard KJ. Comorbidity of migraine with ADHD. *J Atten Disord*. 2012;16(4):339-345. doi: 10.1177/1087054710385784.

*Reason for exclusion: Target population was ADHD with pharmacological treatment*

1. **Frank-Briggs 2011**

Frank-Briggs AI, D Alikor EA. Pattern of paediatric neurological disorders in port harcourt, Nigeria. *Int J Biomed Sci*. 2011;7(2):145-149.

*Reason for exclusion: No diagnosis of ADHD and headache*

1. **Galli 2007**

Galli F, D'Antuono G, Tarantino S, et al. Headache and recurrent abdominal pain: a controlled study by the means of the Child Behaviour Checklist (CBCL). *Cephalalgia*. 2007;27(3):211-219. doi: 10.1111/j.1468-2982.2006.01271.x.

*Reason for exclusion: No diagnosis of ADHD*

1. **Genizi 2013**

Genizi J, Gordon S, Kerem NC, Srugo I, Shahar E, Ravid S. Primary headaches, attention deficit disorder and learning disabilities in children and adolescents. *J Headache Pain*. 2013;14(1):54. doi: 10.1186/1129-2377-14-54.

*Reason for exclusion: Target population was not ADHD; no comparison group*

1. **Genizi 2016**

Genizi J, Marom D, Srugo I, Kerem NC. The Relations between Attention Deficit Hyperactivity Disorder and Headaches in a Non-Clinical Sample of Adolescents. *Acad J Ped Neonatol*. 2016;2(2):555583. doi: 10.19080/AIBM.2016.02.555583

*Reason for exclusion: ADHD was identified by self-report questionnaires*

1. **Ghandour 2010**

Ghandour RM, Kogan MD, Blumberg SJ, Perry DF. Prevalence and correlates of internalizing mental health symptoms among CSHCN. *Pediatrics*. 2010;125(2):e269-e277. doi: 10.1542/peds.2009-0622.

*Reason for exclusion: No data of comorbidity of ADHD and headache provided*

1. **Gladstein 1996**

Gladstein J, Holden EW. Chronic daily headache in children and adolescents: a 2-year prospective study. *Headache*. 1996;36(6):349-351. doi: 10.1046/j.1526-4610.1996.3606349.x.

*Reason for exclusion: No diagnosis of ADHD; target population was not ADHD; no comparison group*

1. **Groenewald 2015**

Groenewald CB, Wright DR, Palermo TM. Health care expenditures associated with pediatric pain-related conditions in the United States. *Pain*. 2015;156(5):951-957. doi: 10.1097/j.pain.0000000000000137.

*Reason for exclusion: No data of comorbidity of ADHD and headache provided*

1. **Güler 2017**

Güler G, Kütük MÖ, Toros F, Özge A, Taşdelen BJ. The high level of psychiatric disorders associated with migraine or tension-type headache in adolescents. *J Neurol Sci*. 2017;34(4):312-321. doi: 10.24165/jns.10112.17

*Reason for exclusion: Target population was not ADHD; no comparison group*

1. **Halfon 2013**

Halfon N, Larson K, Slusser W. Associations between obesity and comorbid mental health, developmental, and physical health conditions in a nationally representative sample of US children aged 10 to 17. *Acad Pediatr*. 2013;13(1):6-13. doi: 10.1016/j.acap.2012.10.007.

*Reason for exclusion: Target population was not ADHD; no data of comorbidity of ADHD and headache provided*

1. **Haynes 2015**

Haynes V, Lopez-Romero P, Anand E. Attention-deficit/hyperactivity disorder Under Treatment Outcomes Research (AUTOR): a European observational study in pediatric subjects. *Atten Defic Hyperact Disord*. 2015;7(4):295-311. doi: 10.1007/s12402-015-0177-y.

*Reason for exclusion: Target population was individuals with ADHD receiving pharmacological treatment*

1. **Holmberg 2010**

Holmberg K. The association of bullying and health complaints in children with attention-deficit/hyperactivity disorder. *Postgrad Med*. 2010;122(5):62-68. doi: 10.3810/pgm.2010.09.2202.

*Reason for exclusion: Same dataset as Holmberg 2006*

1. **Hughes 2021**

Hughes A, Wade KH, Dickson M, et al. Common health conditions in childhood and adolescence, school absence, and educational attainment: Mendelian randomization study. *NPJ Sci Learn*. 2021;6(1):1. doi: 10.1038/s41539-020-00080-6.

*Reason for exclusion: Target population was not ADHD; No diagnosis of ADHD*

1. **Hvidberg 2020**

Hvidberg MF, Johnsen SP, Davidsen M, Ehlers L. A Nationwide Study of Prevalence Rates and Characteristics of 199 Chronic Conditions in Denmark. *Pharmacoecon Open*. 2020;4(2):361-380. doi: 10.1007/s41669-019-0167-7.

*Reason for exclusion:* *The study population including both adolescents and adults; No data of comorbidity of ADHD and headache provided*

1. **Jacobs 2019**

Jacobs H, Pakalnis A. Premonitory Symptoms in Episodic and Chronic Migraine From a Pediatric Headache Clinic. *Pediatr Neurol*. 2019;97:26-29. doi: 10.1016/j.pediatrneurol.2019.03.023.

*Reason for exclusion: Target population was not ADHD; no comparison group*

1. **Jayaprakash 2012**

Jayaprakash R. Clinical profile of children and adolescents attending the behavioural paediatrics unit OPD in a tertiary care set up. *J Indian Assoc Child Adolesc Ment Health* 2012;8(3):51-66.

*Reason for exclusion: Target population was not ADHD; no data of comorbidity of ADHD and headache provided*

1. **Just 2003**

Just U, Oelkers R, Bender S, et al. Emotional and behavioural problems in children and adolescents with primary headache. *Cephalalgia*. 2003;23(3):206-213. doi: 10.1046/j.1468-2982.2003.00486.x.

*Reason for exclusion: No diagnosis of ADHD; target population was not ADHD; no comparison group*

1. **Lateef 2019**

Lateef T, He JP, Nelson K, et al. Physical-Mental Comorbidity of Pediatric Migraine in the Philadelphia Neurodevelopmental Cohort. *J Pediatr*. 2019;205:210-217. doi: 10.1016/j.jpeds.2018.09.033.

*Reason for exclusion: The study population including both children and adults*

1. **Lee 2015**

Lee SM, Yoon JR, Yi YY, et al. Screening for depression and anxiety disorder in children with headache. *Korean J Pediatr*. 2015;58(2):64-68. doi: 10.3345/kjp.2015.58.2.64.

*Reason for exclusion: Target population was not ADHD; no comparison group*

1. **Liakopoulou-Kairis 2002**

Liakopoulou-Kairis M, Alifieraki T, Protagora D, et al. Recurrent abdominal pain and headache--psychopathology, life events and family functioning. *Eur Child Adolesc Psychiatry*. 2002;11(3):115-122. doi: 10.1007/s00787-002-0276-0.

*Reason for exclusion: Target population was not ADHD; no comparison group*

1. **Liu 2018**

Liu T, Lingam R, Lycett K, et al. Parent-reported prevalence and persistence of 19 common child health conditions. *Arch Dis Child*. 2018;103(6):548-556. doi: 10.1136/archdischild-2017-313191.

*Reason for exclusion: No data of comorbidity of ADHD and headache provided*

1. **Lollar 2012**

Lollar DJ, Hartzell MS, Evans MA. Functional difficulties and health conditions among children with special health needs. *Pediatrics*. 2012;129(3):e714-e722. doi: 10.1542/peds.2011-0780.

*Reason for exclusion: No diagnosis of ADHD*

1. **Machnes-Maayan 2014**

Machnes-Maayan D, Elazar M, Apter A, Zeharia A, Krispin O, Eidlitz-Markus T. Screening for psychiatric comorbidity in children with recurrent headache or recurrent abdominal pain. *Pediatr Neurol*. 2014;50(1):49-56. doi: 10.1016/j.pediatrneurol.2013.07.011.

*Reason for exclusion: Target population was not ADHD; no comparison group*

1. **Mahaj 2016**

Mahaj M, Sharkia R, Shorbaji N, Zelnik N. Clinical Profile of Attention Deficit Hyperactivity Disorder: Impact of Ethnic and Social Diversities in Israel. *Isr Med Assoc J*. 2016;18(6):322-325.

*Reason for exclusion: No comparison group; headache was measures as a side effect of ADHD medication*

1. **Mahajnah 2020**

Mahajnah M, Sharkia R, Shorbaji N, Zelnik N. The Clinical Characteristics of ADHD Diagnosed in Adolescents in Comparison With Younger Children. *J Atten Disord*. 2020;24(8):1125-1131. doi: 10.1177/1087054717696768.

*Reason for exclusion: No comparison group; headache was measures as a side effect of ADHD medication*

1. **Mazzone 2006**

Mazzone L, Vitiello B, Incorpora G, Mazzone D. Behavioural and temperamental characteristics of children and adolescents suffering from primary headache. *Cephalalgia*. 2006;26(2):194-201. doi: 10.1111/j.1468-2982.2005.01015.x.

*Reason for exclusion: No diagnosis of ADHD; target population was not ADHD; no comparison group*

1. **Milde-Busch 2010**

Milde-Busch A, Boneberger A, Heinrich S, et al. Higher prevalence of psychopathological symptoms in adolescents with headache. A population-based cross-sectional study. *Headache*. 2010;50(5):738-748. doi: 10.1111/j.1526-4610.2009.01605.x.

*Reason for exclusion: No diagnosis of ADHD; no data of comorbidity of ADHD and headache provided*

1. **Mitchell 2019**

Mitchell BL, Campos AI, Rentería ME, et al. Twenty-Five and Up (25Up) Study: A New Wave of the Brisbane Longitudinal Twin Study. *Twin Res Hum Genet*. 2019;22(3):154-163. doi: 10.1017/thg.2019.27.

*Reason for exclusion: Study population was adults*

1. **Muzina 2011**

Muzina DJ, Chen W, Bowlin SJ. A large pharmacy claims-based descriptive analysis of patients with migraine and associated pharmacologic treatment patterns. *Neuropsychiatr Dis Treat*. 2011;7:663-672. doi: 10.2147/NDT.S25463.

*Reason for exclusion: No diagnosis of ADHD*

1. **Needham 2010**

Needham B, Hill TD. Do gender differences in mental health contribute to gender differences in physical health? *Soc Sci Med*. 2010;71(8):1472-1479. doi: 10.1016/j.socscimed.2010.07.016.

*Reason for exclusion: Study population was adults*

1. **Operto 2018**

Operto FF, Craig F, Peschechera A, Mazza R, Lecce PA, Margari L. Parenting Stress and Emotional/Behavioral Problems in Adolescents with Primary Headache. *Front Neurol*. 2018;8:749. doi: 10.3389/fneur.2017.00749.

*Reason for exclusion: Target population was not ADHD; no comparison group*

1. **Pakalnis 2005**

Pakalnis A, Gibson J, Colvin A. Comorbidity of psychiatric and behavioral disorders in pediatric migraine. *Headache*. 2005;45(5):590-596. doi: 10.1111/j.1526-4610.2005.05113.x.

*Reason for exclusion: Target population was not ADHD; no comparison group*

1. **Pakalnis 2008**

Pakalnis A, Tischner J, Colvin A, Kring D. Emotional and behavioral disorders in pediatric episodic tension headaches. *J Pediatr Neurol*. 2008;6(2):109-113

*Reason for exclusion: Target population was not ADHD; no comparison group*

1. **Park 2017**

Park KJ, Lee JS, Kim HW. Medical and Psychiatric Comorbidities in Korean Children and Adolescents with Attention-Deficit/Hyperactivity Disorder. *Psychiatry Investig*. 2017;14(6):817-824. doi: 10.4306/pi.2017.14.6.817

*Reason for exclusion: No measurement of headache*

1. **Pavone 2012**

Pavone P, Rizzo R, Conti I, et al. Primary headaches in children: clinical findings on the association with other conditions. *Int J Immunopathol Pharmacol*. 2012;25(4):1083-1091. doi: 10.1177/039463201202500425.

*Reason for exclusion: Target population was not ADHD; no comparison group*

1. **Pitrou 2010**

Pitrou I, Shojaei T, Chan-Chee C, Wazana A, Boyd A, Kovess-Masféty V. The associations between headaches and psychopathology: a survey in school children. *Headache*. 2010;50(10):1537-1548. doi: 10.1111/j.1526-4610.2010.01781.x.

*Reason for exclusion: Target population was not ADHD; no comparison group*

1. **Reale 2011**

Reale L, Guarnera M, Grillo C, Maiolino L, Ruta L, Mazzone L. Psychological assessment in children and adolescents with Benign Paroxysmal Vertigo. *Brain Dev*. 2011;33(2):125-130. doi: 10.1016/j.braindev.2010.03.006.

*Reason for exclusion: Target population was not ADHD; no diagnosis of ADHD*

1. **Riva 2012**

Riva D, Usilla A, Aggio F, Vago C, Treccani C, Bulgheroni S. Attention in children and adolescents with headache. *Headache*. 2012;52(3):374-384. doi: 10.1111/j.1526-4610.2011.02033.x.

*Reason for exclusion: Target population was not ADHD; no diagnosis of ADHD*

1. **Romano 2020**

Romano C, Cho SY, Marino S, Raucci U, Fiumara A, Falsaperla R, Massimino CR, Taibi R, Greco F, Venti V, Sullo F, Fontana A, Rizzo R, Pustorino E, Jin DK, Pavone P. Primary headache in childhood associated with psychiatric disturbances: an update. *Eur Rev Med Pharmacol Sci*. 2020;24(12):6893-6898. doi: 10.26355/eurrev_202006_21680.

*Reason for exclusion: Target population was not ADHD; No clear information on the method of ADHD identification*

1. **Salvadori 2007**

Salvadori F, Gelmi V, Muratori F. Present and previous psychopathology of juvenile onset migraine: a pilot investigation by Child Behavior Checklist. *J Headache Pain*. 2007;8(1):35-42. doi: 10.1007/s10194-007-0334-y.

*Reason for exclusion: Target population was not ADHD; no diagnosis of ADHD*

1. **Sarioglu 2003**

Sarioglu B, Erhan E, Serdaroglu G, Doering BG, Erermis S, TutuncuoGlu S. Tension-type headache in children: a clinical evaluation. *Pediatr Int*. 2003;45(2):186-189. doi: 10.1046/j.1442-200x.2003.01678.x.

*Reason for exclusion: Target population was not ADHD; no diagnosis of ADHD*

1. **Slater 2012**

Slater SK, Kashikar-Zuck SM, Allen JR, et al. Psychiatric comorbidity in pediatric chronic daily headache. *Cephalalgia*. 2012;32(15):1116-1122. doi: 10.1177/0333102412460776.

*Reason for exclusion: Target population was not ADHD; no comparison group*

1. **Tegethoff 2015**

Tegethoff M, Belardi A, Stalujanis E, Meinlschmidt G. Comorbidity of Mental Disorders and Chronic Pain: Chronology of Onset in Adolescents of a National Representative Cohort. *J Pain*. 2015;16(10):1054-1064. doi: 10.1016/j.jpain.2015.06.009.

*Reason for exclusion: No comparison group*

1. **Trangkasombat 2008**

Trangkasombat U. Clinical characteristics of ADHD in Thai children. *J Med Assoc Thai*. 2008;91(12):1894-1898.

*Reason for exclusion: No measurement of headache; no comparison group*

1. **Uçar 2020**

Uçar HN, Tekin U, Tekin E. Irritability and its relationships with psychological symptoms in adolescents with migraine: a case-control study. *Neurol Sci*. 2020;41(9):2461-2470. doi: 10.1007/s10072-020-04331-7.

*Reason for exclusion: Target population was not ADHD; no diagnosis of ADHD*

1. **Wade 2008**

Wade TJ, Mansour ME, Line K, Huentelman T, Keller KN. Improvements in health-related quality of life among school-based health center users in elementary and middle school. *Ambul Pediatr*. 2008;8(4):241-249. doi: 10.1016/j.ambp.2008.02.004.

*Reason for exclusion: No measurement of headache*

1. **Wagner 2015**

Wagner JL, Wilson DA, Smith G, Malek A, Selassie AW. Neurodevelopmental and mental health comorbidities in children and adolescents with epilepsy and migraine: a response to identified research gaps. *Dev Med Child Neurol*. 2015;57(1):45-52. doi: 10.1111/dmcn.12555.

*Reason for exclusion: Target population was not ADHD; no comparison group*

1. **Valeriani 2009**

Valeriani M, Galli F, Tarantino S, et al. Correlation between abnormal brain excitability and emotional symptomatology in paediatric migraine. *Cephalalgia*. 2009;29(2):204-213. doi: 10.1111/j.1468-2982.2008.01708.x.

*Reason for exclusion: No diagnosis of ADHD; target population was not ADHD; no comparison group*

1. **Vannatta 2008**

Vannatta K, Getzoff EA, Powers SW, Noll RB, Gerhardt CA, Hershey AD. Multiple perspectives on the psychological functioning of children with and without migraine. *Headache*. 2008;48(7):994-1004. doi: 10.1111/j.1526-4610.2007.01051.x.

*Reason for exclusion: No diagnosis of ADHD; target population was not ADHD; no comparison group*

1. **Warfield 2018**

Warfield ME, Adams RS, Ritter GA, Valentine A, Williams TV, Larson MJ. Health care utilization among children with chronic conditions in military families. *Disabil Health J*. 2018;11(4):624-631. doi: 10.1016/j.dhjo.2018.06.002.

*Reason for exclusion:* *No data of comorbidity of ADHD and headache provided*

1. **Whiteford 2015**

Whiteford HA, Ferrari AJ, Degenhardt L, Feigin V, Vos T. The global burden of mental, neurological and substance use disorders: an analysis from the Global Burden of Disease Study 2010. *PLoS One*. 2015;10(2):e0116820. doi: 10.1371/journal.pone.0116820.

*Reason for exclusion: The study population including both children and adults*

1. **Vigo 2020**

Vigo D, Jones L, Thornicroft G, Atun R. Burden of Mental, Neurological, Substance Use Disorders and Self-Harm in North America: A Comparative Epidemiology of Canada, Mexico, and the United States. *Can J Psychiatry*. 2020;65(2):87-98. doi: 10.1177/0706743719890169.

*Reason for exclusion: The study population including both children and adults*

1. **Wittchen 2011**

Wittchen HU, Jacobi F, Rehm J, et al. The size and burden of mental disorders and other disorders of the brain in Europe 2010. *Eur Neuropsychopharmacol*. 2011;21(9):655-679. doi: 10.1016/j.euroneuro.2011.07.018.

*Reason for exclusion: The study population including both children and adults*

1. **Öztop 2016**

Öztop DB, Taşdelen Bİ, PoyrazoğLu HG, et al. Assessment of Psychopathology and Quality of Life in Children and Adolescents With Migraine. *J Child Neurol*. 2016;31(7):837-842. doi: 10.1177/0883073815623635.

*Reason for exclusion: No diagnosis of ADHD; target population was not ADHD; no comparison group*

**eTable 1.** Summary of characteristics of included case-control/cross-sectional studies (*n* = 13)

| Study | Country, Recruitment/Dataset | Participants (male%) | Age range in years (Mean) | Diagnostic assessment | | Headache type | Matching/  Adjustment | Odds  ratio | the Newcastle-Ottawa Scale | | | | | | | |
| --- | --- | --- | --- | --- | --- | --- | --- | --- | --- | --- | --- | --- | --- | --- | --- | --- |
|  |  |  |  | ADHD | Headache |  |  |  | S1 | S2 | S3 | S4 | C1 | E1 | E2 | E3 |
| Akmatov (2021) | Germany, data of health insurance | ADHD: n = 258,662 (75.6); Control: n = 2,327,958 (75.6) | 5-14 (NR) | ICD-10 | ICD-10 | Migraine | Age, sex, region matched | 2.49 (2.37-2.61) | 0 | 1 | 1 | 1 | 2 | 0 | 1 | 1 |
| Alabaf (2019) | Sweden, CATSS | ADHD: n = 586 (NR); Control: n = 27,472 (NR) | 9, 12 | A-TAC | Parent report | Migraine | N/A | 2.23 (1.62-3.06) | 0 | 0 | 1 | 1 | 0 | 0 | 1 | 0 |
| Arruda (2010) | Brazil, Attention–Brazil Project | ADHD: n = 114 (64.9); Control: n = 1,742 (50.8) | 5-12 (NR) | SNAP-IV, CBCL | Parent report  (the ICHD-2 criteria) | Migraine, Tension headache | N/A | 0.90 (0.56-1.44) | 0 | 1 | 1 | 1 | 0 | 0 | 1 | 0 |
| Arruda (2020) | Brazil, Learning Child | ADHD: n = 303 (71.3); Control: n = 5,368 (50.8) | 5-12 (NR) | SNAP-IV, SDQ | Parent report  (the ICHD-2 criteria) | Migraine, Tension headache | N/A | 1.47 (1.14-1.90) | 0 | 1 | 1 | 1 | 0 | 0 | 1 | 0 |
| Cuffe (2009) | USA,  2001 NHIS | ADHD: n = 278 (69.8); Control: n = 9,243 (50.8) | 4-17 (NR) | SDQ | Parent report | Migraine/headache | N/A | 3.37 (2.47-4.60) | 0 | 1 | 1 | 1 | 0 | 0 | 1 | 0 |
| Holmberg (2006) | Sweden, school students | ADHD: n = 29 (82.8); Control: n = 487 (49.3) | 9-13 (NR) | DSM-IV, Conner-10 items | Self-report | Headache | N/A | 1.06 (0.46-2.45) | 1 | 1 | 1 | 1 | 0 | 0 | 1 | 0 |
| Jameson (2016) | USA,  NCS-A | ADHD: n = 550 (NR); Control: n = 5,933 (NR) | 13-18 (15.2) | DSM-IV | Self-/parent- report | Migraine/headache | Adjusted for age, sex, race, parental education | 1.46 (1.07-1.98) | 1 | 1 | 1 | 1 | 2 | 0 | 1 | 0 |
| Kaplan (1987)^a^ | Canada, hospital | ADHD: n = 40 (75.0); Control: n = 40 (75.0) | 3.5-6 (NR) | DSM-III | Parent report | Headache | Age, sex matched | 14.79 (1.81-121.14) | 1 | 0 | 1 | 1 | 2 | 0 | 1 | 0 |
| Kutuk (2018) | Turkey, hospital | ADHD: n = 117 (82.1); Control: n = 111 (76.6) | 6-18 (NR) | DSM-5 | Clinical diagnosis | Migraine, Tension headache | Age, sex matched | 2.36 (1.39-4.02) | 1 | 1 | 1 | 1 | 2 | 0 | 1 | 0 |
| Lateef (2009) | USA, NHANES | ADHD: n = 899 (NR); Control: n = 10019 (NR) | 4-18 (NR) | Question-naire | Parent-report | Migraine/headache | Adjusted for sex, age, race, income | 2.03 (1.74-2.37) | 0 | 1 | 1 | 1 | 2 | 0 | 1 | 0 |
| Law (2019) | USA,  2012-2015 MEPS | ADHD: n = 2,248 (NR); Control: n = 32,385 (NR) | 2-17 (NR) | Parent report, healthcare providers | Parent-report, healthcare providers | Headache | N/A | 2.25 (1.82-2.78) | 1 | 1 | 1 | 1 | 0 | 1 | 1 | 0 |
| Schieve (2012) | USA,  2006-2010 NHIS | ADHD: n = 2,901 (70.4); Control: n = 35,775 (48.8) | 3-17 (NR) | Question-naire | Parent-report | Migraine/headache | Adjusted for age, sex, race, maternal education | 2.55 (2.26-2.89) | 0 | 1 | 1 | 1 | 2 | 0 | 1 | 0 |
| Strine (2006) | USA,  2003 NHIS | ADHD: n = 879 (70.4); Control: n = 8,385 (48.8) | 4-17 (NR) | SDQ | Parent-report | Headache | Adjusted for age, sex, race, and other^b^ | 2.70 (2.19-3.32) | 0 | 1 | 1 | 1 | 2 | 0 | 1 | 0 |

N/A, Not applicable; NR, Not reported; CATSS, Child and Adolescent Twin Study in Sweden; A-TAC, the Autism-Tics, ADHD and other Comorbidities; ASD, Autism spectrum disorder; LD, Learning disorder; ICHD, International Classification of Headache Disorders; SDQ, the Strengths and Difficulties Questionnaire; NHIS, National Health Interview Survey; NCS-A, the National Comorbidity Survey Replication-Adolescent Supplement; NHANES, the National Health and Nutrition Examination Surveys; MEPS, Medical Expenditure Panel Surveys;

^a^Only study 1 was eligible by the inclusion criteria; however, the data in this study were not included in the meta-analysis due to the outlying odds ratio

^b^Parental structure (single parent household, both parents, and neither parent.), poverty status, type of health care coverage, and number of comorbid conditions.

**
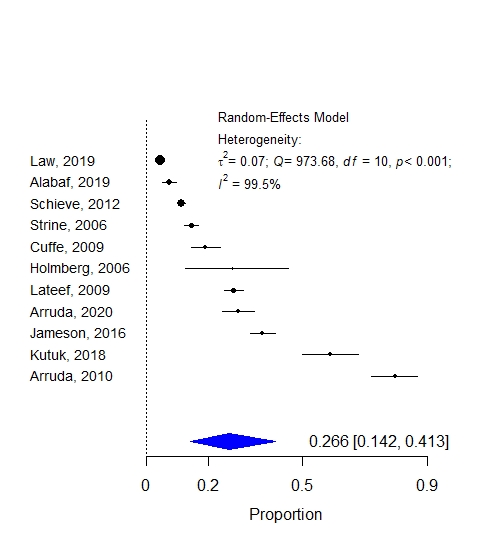
eFigure 1.** Caterpillar plot of pooled prevalence of overall headache in children with ADHD

**eFigure 2.** Funnel plot of epidemiological studies on the association between ADHD and headache among children


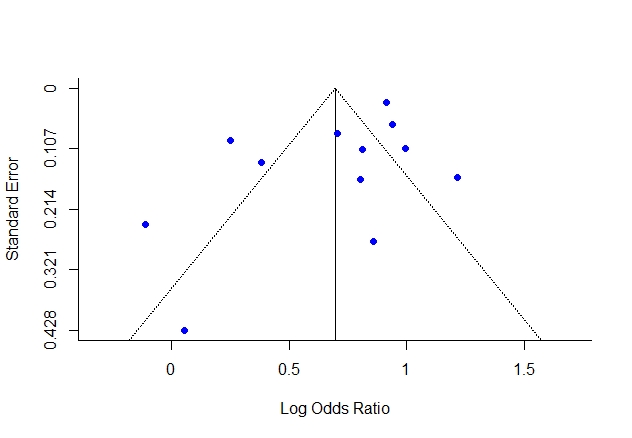


Egger's test: *t* = -1.79, *df* = 10, *p* = 0.104

**eIncluded articles 2.** Studies/citations retained for review question 2 in the present meta-analysis

**Review question 2: Is the use of common medications for ADHD associated with headache in children and adolescents?**

**1. Allen2005, B4Z-MC-LYAS**

- Lewis D, Linder S, Kurlan R, et al. Atomoxetine for the treatment of attention deficit hyperactivity disorder and comorbid tics in children. *Ann Neurol.* 2003;54(Suppl. 7):S106.
- Bangs ME, Allen AJ, Kurlan R, et al. Atomoxetine treatment in children with attention-deficit/hyperactivity disorder and comorbid TIC disorders. *Int J Neuropsychopharmacol*. 2004;7:S441.
- Coffey BJ, Kelsey DK, Feldman PD, et al. Atomoxetine treatment in children with ADHD and comorbid tic disorders. *157th Annual Meeting of the American Psychiatric Association; 2004 May 1-6; New York, NY 2004.*
- Allen AJ, Kurlan RM, Gilbert DL, et al. Atomoxetine treatment in children and adolescents with ADHD and comorbid tic disorders. *Neurology.* 2005;65(12):1941-1949.
- Post hoc subgroup analysis in: Spencer TJ, Sallee FR, Gilbert DL, et al. Atomoxetine treatment of ADHD in children with comorbid Tourette syndrome. *J Atten Disord.* 2008;11(4):470-481.
- Pooled in: Newcorn JH, Sutton VK, Zhang S, et al. Characteristics of placebo responders in pediatric clinical trials of attention-deficit/hyperactivity disorder. *J Am Acad Child Adolesc Psychiatry.* 2009;48(12):1165-1172.
- Commentary in: Anonymous. Atomoxetine may improve tic severity in children with ADHD, Tourette syndrome. *Brown university child & adolescent psychopharmacology update.* 2017;10(3):1, 6-7
- https://assets.contentful.com/hadumfdtzsru/5dD90Njz9uwIc2k00aEASe/ecc2afee105479b66fcc818a322d997d/Atomoxetine-B4Z-MC-LYAS.pdf

**2. Bangs2007, B4Z-MC-LYAX**

- Bangs ME, Emslie GJ, Spencer TJ, et al. A study of atomoxetine in adolescents with ADHD and comorbid depression. *45th Annual NCDEU (New Clinical Drug Evaluation Unit) Meeting; 2005 June 6 - 9; Boca Raton, FL.* *2005*:184.
- Bangs ME, Emslie GJ, Spencer TJ, et al. Efficacy and safety of atomoxetine in adolescents with attentiondeficit/hyperactivity disorder and major depression. *J Child Adolesc Psychopharmacol.* 2007;17(4):407-420.
- Pooled in: Newcorn JH, Sutton VK, Zhang S, et al. Characteristics of placebo responders in pediatric clinical trialsof attention-deficit/hyperactivity disorder. *J Am Acad Child Adolesc Psychiatry.* 2009;48(12):1165-1172.
- http://art45-paediatric-studies-docs.ema.europa.eu/GROUP%20A/Atomoxetine/atomoxetine%20B4Z-MCLYAX%20-%20clinical%20study%20summary.pdf https://www.accessdata.fda.gov/drugsatfda_docs/nda/2002/21-411_Strattera.cfm

**3. Bangs2008 , B4Z-MC-LYBX, NCT00191698**

- Bangs ME, Hazell P, Danckaerts M, et al. Atomoxetine for the treatment of attention-deficit/hyperactivity disorder and oppositional defiant disorder. [Erratum appears in *Pediatrics*. 2008;122(1): 227]. *Pediatrics.* 2008;121(2):e314-320.
- Post hoc analysis in: Hazell P, Becker K, Nikkanen EA, et al. Relationship between atomoxetine plasma concentration, treatment response and tolerability in attention-deficit/hyperactivity disorder and comorbid oppositional defiant disorder. *Atten Defic Hyperact Disord.* 2009;1(2):201-210.
- https://clinicaltrials.gov/ct2/show/NCT00191698
- https://assets.contentful.com/hadumfdtzsru/1S1dKYi8huoyqCecagkagQ/eff47adc1fb45190a286081c7b731169/Atomoxetine-B4Z-MC-LYBX.pdf
- https://www.accessdata.fda.gov/drugsatfda_docs/nda/2002/21-411_Strattera.cfm

**4. Biederman2002, SLI 381-301**

- Lopez FA, Biederman J, Chandler M, Smith W, Boellner SW, Arnold LE. Randomized, controlled trial of a oncedaily amphetamine formulation, SLI381, in children with attention-deficit hyperactivity disorder. *Ann Neurol.* 2001;50(3):S96-S96.
- Biederman J, Lopez FA, Boellner SW, Chandler MC. A randomized, double-blind, placebo-controlled, parallelgroup study of SLI381 (Adderall XR) in children with attention-deficit/hyperactivity disorder. *Pediatrics.* 2002;110(2 Pt 1):258-266.
- Findling RL, McCracken JT, Frazer N, Tulloch SJ. Time course of vital signs after once-daily adderall extended release in ADHD children. *156th Annual Meeting of the American Psychiatric Association; 2003 May 17-22; San* *Francisco, CA 2003:Nr649*
- Findling RL, Biederman J, Wilens TE, et al. Short- and long-term cardiovascular effects of mixed amphetamine salts extended release in children. *J Pediatr.* 2005;147(3):348-354.
- Pooled in: McGough JJ, Biederman J, Wigal SB, et al. Long-term tolerability and effectiveness of once-daily mixed amphetamine salts (Adderall XR) in children with ADHD. *J Am Acad Child Adolesc Psychiatry.* 2005;44(6):530-538.

**5. Biederman2005, Study 311 Cephalon**

- Biederman J, Swanson JM, Lopez FA. Modafinil improves adhd symptoms in children in a randomized, doubleblind, placebo-controlled study. *156th Annual Meeting of the American Psychiatric Association, May 17-22, San* *Francisco CA. 2003*:No. 36.
- Biederman J, Swanson JM, Wigal SB, et al. Efficacy and safety of modafinil film-coated tablets in children and adolescents with attention-deficit/hyperactivity disorder: results of a randomized, double-blind, placebo-controlled, flexible-dose study. *Pediatrics.* 2005;116(6):e777-784.
- Swanson JM, Boellner S, Rugino T, Sangal RB, Wigal SB. Pediatric formulation of modafinil effective in children and adolescents with adhd. *158th Annual Meeting of the American Psychiatric Association; 2005 May 21-26;* *Atlanta, GA.2005*:No. 47.
- Pooled in: Wigal SB, Biederman J, Swanson JM, Yang R, Greenhill LL. Efficacy and safety of modafinil filmcoated tablets in children and adolescents with or without prior stimulant treatment for attentiondeficit/hyperactivity disorder: pooled analysis of 3 randomized, double-blind, placebo-controlled studies. *Prim Care* *Companion J Clin Psychiatry.* 2006;8(6):352-360.
- Barry RJ, Clarke AR. Modafinil improves symptoms of ADHD compared with placebo in young people. *Evid Based Ment Health.* 2006;9(3):68.
- Pooled in: Biederman J, Pliszka SR. Modafinil improves symptoms of attention-deficit/hyperactivity disorder across subtypes in children and adolescents. *J Pediatr.* 2008;152(3):394-399.
- https://www.accessdata.fda.gov/drugsatfda_docs/nda/98/020717A_Provigil.cfm
- Modafinil (CEP-1538) Tablets, Supplemental NDA 20-717/S-019, ADHD Indication Briefing Document for Psychopharmacologic Drugs Advisory Committee Meeting (Document provided by Dr Eric Konofal)

**6. Biederman2006b**

- Biederman J, Swanson JM, Lopez FA. Modafinil improves adhd symptoms in children in a randomized, doubleblind, placebo-controlled study. *156th Annual Meeting of the American Psychiatric Association, May 17-22, San* *Francisco CA. 2003*:No. 36.
- Swanson JM, Greenhill LL, Biederman J. Modafinil in children with ADHD: a randomized, placebo-controlled study. *156th Annual Meeting of the American Psychiatric Association; 2003 May 17-22; San Francisco, CA. 2003*:No. 44.
- Biederman J, Swanson JM, Boellner SW, Lopez E. Modafinil as therapy for ADHD in children: A 4-week, doubleblind, placebo-controlled study. *Eur Neuropsychopharmacol.* 2004;14, S3, S364.
- Swanson JM, Biederman J, Boelliner SW, Lopez FA. Modafinil as therapy for ADHD in children: A 4-week, doubleblind, placebo-controlled study. *J Psychopharmacol.* 2004;18(3, Suppl. S):A53.
- Biederman J, Swanson JM, Wigal SB, Boellner SW, Earl CQ, Lopez FA. A comparison of once-daily and divided doses of modafinil in children with attention-deficit/hyperactivity disorder: a randomized, double-blind, and placebo-controlled study. *J Clin Psychiatry.* 2006;67(5):727-735.

**7. Biederman2007, NRP104-301, NCT00248092**

- Biederman J, Krishnan S, Zhang Y, McGough JJ, Findling RL. Efficacy and tolerability of lisdexamfetamine dimesylate (NRP-104) in children with attention-deficit/hyperactivity disorder: a phase III, multicenter, randomized, double-blind, forced-dose, parallel-group study. *Clin Ther.* 2007;29(3):450-463.
- Lopez FA, Ginsberg LD, Arnold V. Effect of lisdexamfetamine dimesylate on parent-rated measures in children aged 6 to 12 years with attention-deficit/hyperactivity disorder: a secondary analysis. *Postgrad Med.* 2008;120(3):89-102.
- Findling RL, Adeyi B, Chen G, et al. Clinical response and symptomatic remission in children treated with lisdexamfetamine dimesylate for attention-deficit/hyperactivity disorder. *CNS Spectr.* 2010(9):559-568.
- Pooled for a post hoc analysis in: Goodman D, Faraone SV, Adler LA, Dirks B, Hamdani M, Weisler R. Interpreting ADHD rating scale scores: Linking ADHD rating scale scores and CGI levels in two randomized controlled trials of lisdexamfetamine dimesylate in ADHD. *Primary Psychiatry.* 2010;17(3):44-52.
- Pooled for a post hoc analysis in: Waxmonsky JG, Waschbusch DA, Glatt SJ, Faraone SV. Prediction of placebo response in 2 clinical trials of lisdexamfetamine dimeslylate for the treatment of ADHD. *J Clin Psychiatry.* 2011;72(10):1366-1375.
- Post hoc analysis in: Jain R, Babcock T, Burtea T, et al. Efficacy of lisdexamfetamine dimesylate in children with attention-deficit/hyperactivity disorder previously treated with methylphenidate: a post hoc analysis. *Child Adolesc* *Psychiatry Ment Health.* 2011;5(1):35. (NCT00556296)-
- Previous reference related to: Conference proceeding: Jain R, Babcock T, Burtea T, et al. Efficacy of lisdexamfetamine dimesylate in children with attention-deficit/hyperactivity disorder and suboptimal response to methylphenidate. *163rd Annual Meeting of the American Psychiatric Association; 2010 May 22-26; New Orleans,* *LA* 2010.
- Jain R, Duncan D, Babcock T, et al. Lisdexamfetamine dimesylate (LDX) in children with ADHD after suboptimal response to methylphenidate. *Eur Child Adolesc Psychiatry.* 2011;20:S126-S127.
- Pooled for a post hoc analysis in: McGough JJ, Greenbaum M, Adeyi B, et al. Sex subgroup analysis of treatment response to lisdexamfetamine dimesylate in children aged 6 to 12 years with attention-deficit/hyperactivity disorder. *J Clin Psychopharmacol.* 2012;32(1):138-140.
- Included in: Coghill D, Sorooshian S, Caballero B. Safety outcomes from the clinical development programme for lisdexamfetamine dimesylate: A prodrug stimulant for the treatment of attention-deficit/hyperactivity disorder. *Eur* *Child Adolesc Psychiatry*. 2013;1):S224.
- Post hoc analysis in: Childress AC, Arnold V, Adeyi B, et al. The effects of lisdexamfetamine dimesylate on emotional lability in children 6 to 12 years of age with ADHD in a double-blind placebo-controlled trial. *J Atten* *Disord.* 2014;18(2):123-132.
- Used in a post hoc analysis in: Weisler RH, Adler LA, Kollins SH, et al. Analysis of individual items on the attention deficit/hyperactivity disorder symptom rating scale in children and adults: The effects of age and sex in pivotal trials of lisdexamfetamine dimesylate. *Neuropsychiatr Dis Treat.* 2014;10:1-12.
- Lopez FA, Childress A, Adeyi B, et al. ADHD Symptom Rebound and Emotional Lability With Lisdexamfetamine Dimesylate in Children Aged 6 to 12 Years. *J Atten Disord.* 2017;21(1):52-61.
- https://clinicaltrials.gov/ct2/show/NCT00248092
- https://www.accessdata.fda.gov/drugsatfda_docs/nda/2007/021977s000TOC.cfm

**8. Biederman2008, SPD503-301, NCT00152009**

- Biederman J, Melmed RD, Patel A, et al. A randomized, double-blind, placebo-controlled study of guanfacine extended release in children and adolescents with attention-deficit/hyperactivity disorder. *Pediatrics.* 2008;121(1):e73-84.
- Open label continuation phase: Biederman J, Melmed RD, Patel A, McBurnett K, Donahue J, Lyne A. Long-term, open-label extension study of guanfacine extended release in children and adolescents with ADHD. *CNS Spectr.* 2008;13(12):1047-1055.
- Pooled in: Faraone SV, Glatt SJ. Effects of extended-release guanfacine on ADHD symptoms and sedation-related adverse events in children with ADHD. *J Atten Disord.* 2010;13(5):532-538.
- Pooled in: Sallee FR, Kollins SH, Wigal TL. Efficacy of guanfacine extended release in the treatment of combined and inattentive only subtypes of attention-deficit/hyperactivity disorder. *J Child Adolesc Psychopharmacol*. 2012;22(3):206-214.
- Pooled in: Huss M, McBurnett K, Cutler AJ, et al. Separating efficacy and sedative effects of guanfacine extended release in children and adolescents with ADHD from four randomized, controlled, phase 3 clinical trials. *Eur* *Psychiatry.* 2016;33:S76-S77.
- Pooled in: Huss M, McBurnett K, Cutler AJ, Hervás A, Bliss C, Gao J, Dirks B, Newcorn JH. Distinguishing the efficacy and sedative effects of guanfacine extended release in children and adolescents with attention-deficit/hyperactivity disorder. Eur Neuropsychopharmacol. 2019;29(3):432-443. and in Faraone SV, Glatt SJ. Effects of extended-release guanfacine on ADHD symptoms and sedation-related adverse events in children with ADHD. J Atten Disord. 2010;13(5):532-8.
- https://clinicaltrials.gov/ct2/show/NCT00152009
- https://www.accessdata.fda.gov/drugsatfda_docs/nda/2009/022037_intuniv_toc.cfm
- http://www.shirecanada.com/-/media/shire/shireglobal/shirecanada/pdffiles/product%20information/intuniv-xr-pmen.pdf

**9. Block2009, B4Z-US-LYCC, NCT00486122**

- Sutton V, Kelsey D, Lewis D, Schuh K, Sumner C, Quintana H. Morning-dosed Versus evening-dosed atomoxetinefor treating ADHD in children. *158th Annual Meeting of the American Psychiatric Association; 2005 May 21-26;* *Atlanta, GA.* 2005.
- Kelsey DK, Sutton V, Lewis D, Schuh K, Sumner C, Quintana H. Morning-versus evening-dosed atomoxetine: effects on core ADHD symptoms. *158th Annual Meeting of the American Psychiatric Association; 2005 May 21-26; Atlanta, GA, 2005*
- Related: Kratochvil CJ, Faries D, Vaughan B, et al. Emotional expression during attention-deficit/hyperactivity disorders treatment: initial assessment of treatment effects. *J Child Adolesc Psychopharmacol.* 2007;17(1):51-62.
- Block SL, Kelsey D, Coury D, et al. Once-daily atomoxetine for treating pediatric attention-deficit/hyperactivity disorder: comparison of morning and evening dosing. *Clin Pediatr (Phila).* 2009;48(7):723-733.
- Pooled in: Newcorn JH, Sutton VK, Zhang S, et al. Characteristics of placebo responders in pediatric clinical trials of attention-deficit/hyperactivity disorder. *J Am Acad Child Adolesc Psychiatry.* 2009;48(12):1165-1172.
- Pooled in: Block SL, Williams D, Donnelly CL, Dunn DW, Saylor KE, Ruberg SJ. Post hoc analysis: early changes in ADHD-RS items predict longer term response to atomoxetine in pediatric patients. *Clin Pediatr (Phila).* 2010;49(8):768-776.
- https://clinicaltrials.gov/ct2/show/NCT00486122
- https://assets.contentful.com/hadumfdtzsru/33eUP3LTUcsAgCAawSQ4Yy/25d05bd80b6f4f5ba59a7c332464835b/Atomoxetine-B4Z-US-LYCC.pdf
- https://www.accessdata.fda.gov/drugsatfda_docs/nda/2002/21-411_Strattera.cfm

**10. Brams2018, NCT02466425**

- Brams M, Childress AC, Greenbaum M, Yu M, Yan B, Jaffee M, Robertson B. SHP465 Mixed Amphetamine Salts in the Treatment of Attention-Deficit/Hyperactivity Disorder in Children and Adolescents: Results of a Randomized, Double-Blind Placebo-Controlled Study. J Child Adolesc Psychopharmacol. 2018;28(1):19-28.
- Brams, M., Childress, A., Greenbaum, M. S., Yu, M., Yan, B., Jaffee, M., et al. Efficacy and safety of SHP465 mixed amphetamine salts in children and adolescents with attention-deficit/hyperactivity disorder (ADHD): A randomized, placebo-controlled study. (10), S165.

**11. Childress2009, CRIT124E2305, NCT00301236**

- Childress A, Muniz R, Miller J, Arnold V, HarperL, Gerstner O, et al. Fixed-dose titration study of dexmethylphenidate extended release in children withADHD: effects on teacher-rated scales. Ann Neurol2007;62(Suppl 11):S125.
- Greenbaum M, Muniz R, Brams M, Boellner S,Gerstner O, Borello M, et al. Cardiovascular safety of dexmethylphenidate extended release in children with ADHD. Ann Neurol 2007;62(Suppl 11):S146.
- Lopez F, Muniz R, Joyce JM, Franklin ER, Gerstner O, Borello M, et al. Fixed-dose titration study of dexmethylphenidate extended release in children with ADHD: effects on parent-rated scales. *Ann Neurol* 2007;62(Suppl 11):S122.
- Childress AC, Spencer T, Lopez F, et al. Efficacy and safety of dexmethylphenidate extended-release capsules administered once daily to children with attention-deficit/hyperactivity disorder. *J Child Adolesc Psychopharmacol.* 2009;19(4):351-361.
- Anonymous. Dose related safety and efficacy of dexmethylphenidate ER. The Brown University Child & Adolescent. Psychopharmacology Update 2009;11,11:7–8.
- https://clinicaltrials.gov/ct2/show/NCT00301236 (additional ID: CRIT124E2305)
- <https://www.novctrd.com/CtrdWeb/displaypdf.nov?trialresultid=2418>

**12. Coghill2013, SPD489-325**

- Coghill DR, Tobias B, Lecendreux ML, et al. Efficacy and safety of lisdexamfetamine dimesylate in children and adolescents with attention- deficit/hyperactivity disorder: a Phase 3, randomized, double-blind, multicenter, parallelgroup, placebo-and-active-controlled, dose- optimization study in Europe. Proceedings of the American Academy of Child and Adolescent Psychiatry and Canadian Academy of Child and Adolescent Psychiatry Joint Annual Meeting; October 18–23, 2011; Toronto, Canada
- Gasior M, Coghill D, Soutullo C, Lyne A, Johnson M. Efficacy and safety of lisdexamfetamine dimesylate in children and adolescents with ADHD: A phase 3, randomized, double-blind, multicenter, parallel-group, placeboand active-controlled, dose-optimized study in Europe. *Acta Neuropsychiatrica.* 2012;24:24.
- Coghill DR, Banaschewski T, Lecendreux M, et al. The first European study of lisdexamfetamine dimesylate in children and adolescents with ADHD: Overview. *Neuropsychiatr Enfance Adolesc*. 2012;1):S76.
- Coghill D, Banaschewski T, Lecendreux M, et al. Maintenance of efficacy of lisdexamfetamine dimesylate in children and adolescents with ADHD: Randomised withdrawal design. *Eur Neuropsychopharmacol.* 2012;22:S431-S432. (Withdrawal extension phase NCT00784654, SPD489-326 (EUCTR2008-000720-10-DE)
- Hodgkins P, Coghill DR, Soutullo CA, Bloomfield R, Gasio RM, JohnsonM. Poster 14: Effect of isdexamfetamine dimesylate on functional impairment in children and adolescents with attention-deficit/hyperactivity disorder. Acta Neuropsychiatrica. Abstracts of the Scandinavian College of Neuropsychopharmacology (SCNP) 53rd Annual Meeting; 2012 April 25-27; Copenhagen, Denmark 2012;24(Suppl s1):27–8.
- Soutullo CA, Banaschewski T, Lecendreux ML, Bloomfield R, Hodgkins P, Coghill DR. Effect of lisdexamfetamine dimesylate on functional impairment in children and adolescents with attention-deficit/hyperactivity disorder. *Eur* *Psychiatry.* 2012;27.
- Soutullo C, Banaschewski T, Lecendreux M, et al. Effect of lisdexamfetamine dimesylate on functional impairment in children and adolescents with ADHD. *Neuropsychiatr Enfance Adolesc.* 2012;1):S76.
- Zuddas A, Banaschewski T, Lecendreux M, et al. Clinical efficacy of lisdexamfetamine dimesylate in children and adolescents with ADHD: A post-hoc analysis. *Eur Neuropsychopharmacol.* 2012;22:S431.
- Zuddas A, Banaschewski T, Lecendreux M, et al. Duration of response of lisdexamfetamine dimesylate in children and adolescents with ADHD. *Neuropsychiatr Enfance Adolesc.* 2012;1):S76.
- Zuddas A, Coghill DR, Banaschewski T, et al. Weight-related safety outcomes of lisdexamfetamine dimesylate in children and adolescents with ADHD: Post-hoc analysis from a phase 3, randomized, double-blind, multicentre, parallel-group, placebo-and active-controlled, dose-optimized study in Europe. *Neuropsychiatr Enfance Adolesc.* 2012;1):S263.
- Lecendreux ML, Dittmann RW, Gasior M, et al. Psychiatric-related safety outcomes of lisdexamfetamine dimesylate in children and adolescents with attention-deficit/hyperactivity disorder: A phase 3, randomized, doubleblind, multi centre, parallel-group, placebo-and active-controlled, dose-optimized study in Europe. *Neuropsychiatr* *Enfance Adolesc.* 2012;1):S263.
- Banaschewski T, Johnson M, Zuddas A, et al. Health-related quality of life outcomes in children and adolescents with ADHD treated with lisdexamfetamine dimesylate. *Neuropsychiatr Enfance Adolesc.* 2012;1):S77.
- Setyawan J, Banaschewski T, Hodgkins P, et al. Health utility scores in children and adolescents with attentiondeficit/ hyperactivity disorder: Response to stimulant treatment. *Value Health.* 2012;15 (7):A284.
- Coghill D, Banaschewski T, Lecendreux M, Soutullo C, Johnson M, Zuddas A, et al. Post hoc comparison of the efficacy of lisdexamfetamine dimesylate and osmotic-release oral system methylphenidate in children and adolescents with ADHD. *Eur Psychiatry*. Abstracts of the 21th European Congress of Psychiatry 2013;28(Suppl 1)
- Coghill D, Banaschewski T, Lecendreux M, et al. Impact of previous ADHD medication on the efficacy of lisdexamfetamine dimesylate in the treatment of ADHD: Post hoc analyses. *Eur Neuropsychopharmacol.* 2013;23:S599-S600.
- Coghill D, Banaschewski T, Lecendreux M, et al. European, randomized, phase 3 study of lisdexamfetamine dimesylate in children and adolescents with attention-deficit/hyperactivity disorder. *Eur Neuropsychopharmacol.* 2013;23(10):1208-1218.
- Banaschewski T, Soutullo C, Lecendreux M, et al. Health-related quality of life and functional outcomes from a randomized, controlled study of lisdexamfetamine dimesylate in children and adolescents with attention deficit hyperactivity disorder. *CNS Drugs.* 2013;27(10):829-840.
- Hodgkins P, Setyawan J, Banaschewski T, et al. Health utility scores in children and adolescents with attentiondeficit/hyperactivity disorder: Response to stimulant treatment. *Eur Child Adolesc Psychiatry.* 2013;1):S127.
- Lecendreux M, Banaschewski T, Soutullo C, et al. Efficacy of lisdexamfetamine dimesylate in children and adolescents with attention-deficit/hyperactivity disorder: Effect of age, sex and baseline disease severity. *Eur* *Psychiatry.* 2013;28.
- Coghill D, Banaschewski T, Lecendreux M, et al. The first European studies of lisdexamfetamine dimesylate in children and adolescents with attention-deficit/hyperactivity disorder. *Eur Child Adolesc Psychiatry*. 2013;1):S125.
- Coghill D, Banaschewski T, Zuddas A, et al. Health-related quality of life outcomes in a long-term study of lisdexamfetamine dimesylate in children and adolescents with ADHD. *Eur Neuropsychopharmacol.* 2013;23:S598-S9. (extension phase: NCT00784654, SPD489-326 (EUCTR2008-000720-10-DE)
- Banaschewski T, Soutullo C, Lecendreux M, et al. The child health and illness profile as a measure of health-related quality of life in stimulant-treated children and adolescents with ADHD. *Eur Child Adolesc Psychiatry.*2013;1):S125-S126. (refers to both SPD489-325 and SPD489-326)
- Lecendreux M, Zuddas A, Banaschewski T, et al. Weight-related safety outcomes of lisdexamfetamine dimesylate in children and adolescents with attention-deficit/hyperactivity disorder. *Eur Child Adolesc Psychiatry.* 2013;1:S223.
- Sorooshian S, Banaschewski T, Lecendreux M, et al. Post hoc comparison of the efficacy of lisdexamfetamine dimesylate and osmotic-release oral system methylphenidate in children and adolescents with attention deficit/hyperactivity disorder. *Aust N Z J Psychiatry.* 2013;47:89-90.
- Sorooshian S, Markarian M, Ermer J, Civil R, Gasior M, Coghill D. Pharmacokinetic and clinical profiles of the longacting prodrug stimulant lisdexamfetamine dimesylate: Results from two studies in children and adolescents with attention deficit/hyperactivity disorder. *Aust N Z J Psychiatry.* 2013;47:89.
- Soutullo C, Banaschewski T, Lecendreux M, et al. A post hoc comparison of the effects of lisdexamfetamine dimesylate and osmotic-release oral system methylphenidate on symptoms of attention-deficit hyperactivity disorder in children and adolescents. *CNS Drugs.* 2013;27(9):743-751.
- Soutullo C, Banaschewki T, Lecendreux M, et al. Assessing ADHD stimulant treatment efficacy using the Weiss functional impairment rating scale: Strengths and weaknesses. *Eur Child Adolesc Psychiatry.* 2013;1):S126.
- Coghill DR, Banaschewski T, Lecendreux M, et al. Efficacy of lisdexamfetamine dimesylate throughout the day in children and adolescents with attention-deficit/hyperactivity disorder: results from a randomized, controlled trial. *Eur Child Adolesc Psychiatry.* 2014;23(2):61-68.
- Fridman M, Erder MH. Interpreting the Results of a Retrospective Comparison of Test and Reference Treatments in a Randomized Clinical Trial Setting. *Clin Drug Investig.* 2014;35(2):133-140.
- Coghill DR, Banaschewski T, Lecendreux M, et al. Post hoc analyses of the impact of previous medication on the efficacy of lisdexamfetamine dimesylate in the treatment of attention-deficit/hyperactivity disorder in a randomized, controlled trial. *Neuropsychiatr Dis Treat.* 2014;10:2039-2047.
- Doddamani L, Hodgkins P, Adeyi B, Squires LA, Civil R, Coghill DR. Functional impairment in children and adolescents with attention deficit hyperactivity disorder: Results from short-and long-term studies of lisdexamfetamine dimesylate. *Aust N Z J Psychiatry.* May 2014;48:115.
- Banaschewski T, Johnson M, Lecendreux M, et al. Health-related quality of life and functional outcomes from a randomized-withdrawal study of long-term lisdexamfetamine dimesylate treatment in children and adolescents with attention-deficit/hyperactivity disorder. *CNS Drugs.* 2014;28(12):1191-1203. (withdrawal extension phase: NCT00784654, SPD489-326 (EUCTR2008-000720-10-DE)
- Coghill DR, Banaschewski T, Lecendreux M, Johnson M, Zuddas A, Anderson CS, Civil R, Dauphin M, Higgins N, Lyne A, Gasior M, Squires LA. Maintenance of efficacy of lisdexamfetamine dimesylate in children and adolescents with attention-deficit/hyperactivity disorder: randomized-withdrawal study design. *J Am Acad Child Adolesc* *Psychiatry*. 2014;53(6):647-657.e1 (Withdrawal extension: NCT00784654, SPD489-326 (EUCTR2008-000720-10-DE)
- Doddamani L, Sikirica V, Caballero B, Adeyi B, Naser N, Coghill D. Post HOC comparison of lisdexamfetamine dimesylate and osmotic-release oral system methylphenidate: Symptoms, health-related quality of life and functional impairment in children and adolescents with attention deficit/hyperactivity disorder. *Aust N Z J* *Psychiatry.* 2015;49:108.
- Included in: Coghill DR, Nagy P, Frick G, Adeyi B, Newcorn J. Relative efficacy of lisdexamfetamine dimesylate and osmotic controlled-release methylphenidate in attention-deficit/ hyperactivity disorder patients. *Eur* *Neuropsychopharmacol*. 2015;25:S647-S8
- Grebla R, Setyawan J, Yang H, et al. Development of a risk score to guide individualized treatment selection in attention-deficit/hyperactivity disorder. *Eur Child Adolesc Psychiatry.* 2015;1):S244.
- Setyawan J, Yang H, Cheng D, et al. Developing a Risk Score to Guide Individualized Treatment Selection in Attention Deficit/Hyperactivity Disorder. *Value Health.* 2015;18(6):824-831.
- Zuddas A, Banaschewski T, Nagy P, et al. Long-term safety and efficacy of lisdexamfetamine dimesylate in children and adolescents with attention-deficit/hyperactivity disorder. *Eur Neuropsychopharmacol.* 2015;25:S648. (Extension phase: NCT00784654, SPD489-326 (EUCTR2008-000720-10-DE)
- Coghill D, Nagy P, Frick G, Naser N, Wu J, Newcorn J. Comparing the time-course of efficacy of lisdexamfetamine dimesylate and osmotic controlled-release methylphenidate in children and adolescents with ADHD. Australian and New Zealand journal of psychiatry 2016; 50, 114‐115. Retrieved from https://www.cochranelibrary.com/central/doi/10.1002/central/CN-01719602/full doi:10.1177/0004867416640967
- Post hoc analyses in: Coghill DR., Joseph A., Sikirica V, Kosinski M., Bliss C., Huss M. Correlations Between Clinical Trial Outcomes Based on Symptoms, Functional Impairments, and Quality of Life in Children and Adolescents With ADHD. J Atten Disord 2017, 1087054717723984. doi:10.1177/1087054717723984
- <https://clinicaltrials.gov/ct2/show/NCT00763971>
- Analysed in: Coghill D, Nagy P, Frick G, Naser N, Wu J, Newcorn J. Comparing the time course of efficacy of lisdexamfetamine dimesylate and osmotic controlled-release methylphenidate in children and adolescents with ADHD. *Aust N Z J Psychiatry.* 2016; 50, 114‐115. Retrieved from https://www.cochranelibrary.com/central/doi/10.1002/central/CN-01719602/full doi:10.1177/0004867416640967

**13. Connor2010, SPD503-307, NCT00367835**

- Sallee F, Spencer T, Connor D, Lopez F, Lyne A, Tremblay G. Effects of Guanfacine Extended Release on Secondary Measures in Children With Attention-Deficit/Hyperactivity Disorder and Oppositional Symptoms. *Biol* *Psychiatry.* 2009;65(8, Suppl. S):149S.
- Connor DF, Findling RL, Kollins SH, et al. Effects of guanfacine extended release on oppositional symptoms in children aged 612 years with attention-deficit hyperactivity disorder and oppositional symptoms: A randomized, double-blind, placebo-controlled trial. *CNS Drugs.* 2010;24(9):755-768.
- Pooled in: Huss, M., Newcorn, J., Connor, D., Hervas, A., Werner-Kiechle, T., & Robertson, B. (2017). Efficacy of guanfacine extended release in children and adolescents with ADHD and comorbid oppositional defiant disorder. *European Neuropsychopharmacology, 27 (Supplement 4)*, S1114.
- Connor D, Kollins S, Findling R, et al. Effects of Guanfacine Extended Release in Children Aged 6 to 12 Years with Oppositional Symptoms and a Diagnosis of Attention-Deficit/Hyperactivity Disorder. *Biol Psychiatry.* 2010;67:217S.
- Connor DF, Sallee FR, Lyne A, Stat C, Youcha S. Changes in parental stress with guanfacine extended release in children with attention-deficit/ hyperactivity disorder and oppositional symptoms. *163rd Annual Meeting of the* *American Psychiatric Association; 2010 May 22-26; New Orleans, LA 2010.*
- https://clinicaltrials.gov/ct2/show/NCT00367835 (other ID:SPD503-307**)**

**14. CRIT124DUS02**

- https://www.novctrd.com/CtrdWeb/displaypdf.nov?trialresultid=1624

**15. Daviss 2008, NCT00031395**

- Palumbo DR, Sallee FR, Pelham WE, Bukstein OG, Daviss WB, McDermott MP. Clonidine for attentiondeficit/hyperactivity disorder treatment study: I. Efficacy and tolerability outcomes. *J Am Acad Child Adolesc* *Psychiatry*. 2008;47:179-187.
- Daviss, WB, Patel NC, Robb AS et al. Clonidine for attention-deficit/hyperactivity disorder: II. ECG changes and adverse events analysis. *J Am Acad Child Adolesc Psychiatry*. 2008;47(2):189-98.
- Cannon M, Pelham WH, Sallee FR, Palumbo DR, Bukstein O, Daviss WB. Effects of clonidine and methylphenidate on family quality of life in attention-deficit/hyperactivity disorder. *J Child Adolesc Psychopharmacol*. 2009;19(5):511-517.
- <https://clinicaltrials.gov/ct2/show/NCT00031395>

**16. Dell’Agnello2009**

- Dell'Agnello G, Maschietto D, Bravaccio C, et al. Atomoxetine hydrochloride in the treatment of children and adolescents with attention-deficit/hyperactivity disorder and comorbid oppositional defiant disorder: A placebocontrolled Italian study. *Eur Neuropsychopharmacol.* 2009;19(11):822-834.
- Dell'Agnello G, Maschietto D, Pascotto A, et al. Efficacy of atomoxetine in pediatric patients with attentiondeficit/hyperactivity disorder and comorbid oppositional defiant disorder. *World Psychiatry*. 2009:252.
- Pooled in: Montoya A, Quail D, Anand E, Cardo E, Alda JA, Escobar R. Prognostic factors of improvement in health-related quality of life in atomoxetine-treated children and adolescents with attention-deficit/hyperactivity disorder, based on a pooled analysis. *Atten Defic Hyperact Disord.* 2014;6(1):25-34.
- Previous reference related to: Montoya A, Quail D, Anand E, Cardo E, Alda JA, Escobar R. Prognostic factors of improvement in health related quality of life in children and adolescents with attention deficit/hyperactivity disorder, after atomoxetine treatment. *Eur Psychiatr.* 2012;27).
- <https://clinicaltrials.gov/ct2/show/NCT00192023>

**17. Dittmann2011**

- Dittmann R.W, Schacht A, Helsberg K, Schneider-Fresenius C, Lehmann M, Lehmkuhl G, Wehmeier PM. Randomisierte, plazebokontrollierte Doppelblindstudie zur Wir- ksamkeit und Vertra ̈glichkeit von Atomoxetin (ATX) bei Kindern und Jugendlichen mit ADHS und oppositionellem Trotzverhalten bzw. Sto ̈rung des Sozialverhaltens. In Frank Schneider und Michael Gro ̈zinger (Eds.), Psychiatric disorders through lifespan. Abstract book of the DGPPN congress 2009, 25–28 November 2009 (p. 230). Germany: ICC Berlin. doi:10.3287/dgppn.2009.1
- Dittmann RW, Schacht A, Helsberg K, et al. Atomoxetine versus placebo in children and adolescents with attentiondeficit/ hyperactivity disorder and comorbid oppositional defiant disorder: a double-blind, randomized, multicenter trial in Germany. *J Child Adolesc Psychopharmacol.* 2011;21(2):97-110.
- Wehmeier PM, Schacht A, Dittmann RW, et al. Effect of atomoxetine on quality of life and family burden: results from a randomized, placebo-controlled, double-blind study in children and adolescents with ADHD and comorbid oppositional defiant or conduct disorder. *Qual Life Res.* 2011;20(5):691-702.
- https://www.clinicaltrialsregister.eu/ctr-search/search?query=eudract_number:2006-001471-37 (additional ID:B4ZSB-LYDW**)**
- https://clinicaltrials.gov/ct2/show/NCT00406354

**18. Findling2010, NCT00499863, SPD485-40**

- Findling RL, Turnbow J, Burnside J, Melmed R, Civil R, Li Y. A randomized, double-blind, multicenter, parallelgroup, placebo-controlled, dose-optimization study of the methylphenidate transdermal system for the treatment of ADHD in adolescents. *CNS Spectr.* 2010;15(7):419-430.
- Extension in: Findling RL, Katic A, Rubin R, Moon E, Civil R, Li Y. A 6-month, open-label, extension study of the tolerability and effectiveness of the methylphenidate transdermal system in adolescents diagnosed with attentiondeficit/hyperactivity disorder. *J Child Adolesc Psychopharmacol.* 2010;20(5):365-375.
- Keating GM. Methylphenidate transdermal system in attention-deficit hyperactivity disorder in adolescents: profile report. *Drugs in R&D.* 2012;12(3):171–3.
- <https://clinicaltrials.gov/ct2/show/NCT00499863>

**19. Findling2011, SPD 489-305, NCT00735371**

- Childress A, Cutler A, Gasior M, et al. Double-Blind, Placebo-Controlled Efficacy and Safety Study of Lisdexamfetamine Dimesylate in Adolescents with Attention Deficit Hyperactivity Disorder (ADHD). *J Child* *Adolesc Psychopharmacol.* 2010;20(6):533-533.
- Gasior M, Findling R, Childress A, Cutler A, Hamdani M, Ferreira-Cornwell MC. Double-blind, placebo-controlled efficacy and safety study of lisdexamfetamine dimesylate in adolescents with attention-deficit/ hyperactivity disorder. Neuropsychopharmacology*.* 2010;35:S103-S104.
- Findling RL, Childress AC, Cutler AJ, et al. Efficacy and safety of lisdexamfetamine dimesylate in adolescents with attention-deficit/hyperactivity disorder. *J Am Acad Child Adolesc Psychiatry.* 2011;50(4):395-405.
- Findling R, Childress A, Cutler A, et al. Long-term safety of lisdexamfetamine dimesylate (LDX) in adolescents with attention-deficit/hyperactivity disorder. *Eur Child Adolesc Psychiatry.* 2011;20:S117-S118.
- Included in: Coghill D, Sorooshian S, Caballero B. Safety outcomes from the clinical development programme for lisdexamfetamine dimesylate: A prodrug stimulant for the treatment of attention-deficit/hyperactivity disorder. *Eur* *Child Adolesc Psychiatry*. 2013;1):S224.
- Open phase in: Findling RL, Cutler AJ, Saylor K, et al. A long-term open-label safety and effectiveness trial of lisdexamfetamine dimesylate in adolescents with attention-deficit/hyperactivity disorder. *J Child Adolesc* *Psychopharmacol.* 2013;23(1):11-21.
- Post hoc analysis of long term phase: Childress AC, Cutler AJ, Saylor K, et al. Participant-perceived quality of life in a long-term, open-label trial of lisdexamfetamine dimesylate in adolescents with attention-deficit/hyperactivity disorder. *J Child Adolesc Psychopharmacol.* 2014;24(4):210-217.
- https://clinicaltrials.gov/ct2/show/NCT00735371 (additional ID: SPD489-305)
- https://www.accessdata.fda.gov/drugsatfda_docs/nda/2007/021977s000TOC.cfm

**20. Froehlich 2018, NCT01727414**

- Hsu S, Piedra A, Froehlich T, Brinkman W, Epstein, J. Caregiver-reported side effects of methylphenidate and comorbid oppositional defiant disorder diagnosis in children with attention-deficit/hyperactivity disorder (ADHD). *Journal of the american academy of child and adolescent psychiatry. Conference: 64th annual meeting american academy of child and adolescent psychiatry, AACAP 2017. United states, 56*(10), S282. Retrieved from http://cochranelibrary-wiley.com/o/cochrane/clcentral/articles/373/CN-01452373/frame.html doi:10.1016/j.jaac.2017.09.363
- Froehlich, T. E., Becker, S. P., Nick, T. G., Brinkman, W. B., Stein, M. A., Peugh, J., & Epstein, J. N. (2018). Sluggish Cognitive Tempo as a Possible Predictor of Methylphenidate Response in Children With ADHD: A Randomized Controlled Trial. *J Clin Psychiatry, 79*(2). doi:10.4088/JCP.17m11553
- Includes: Orban S, Karamchandani T, Tamm L, Sidol C, Peugh J, Froehlich T, Brinkman WB, Estell N, Mii A, Epstein J. Attention-deficit/hyperactivity disorder-related deficits and psychostimulant medication effects on comprehension of audiovisually presented educational material in children. J Child Adolesc Psychopharmacology 2018; 28(10): 727‐738. Retrieved from https://www.cochranelibrary.com/central/doi/10.1002/central/CN-01726809/full doi:10.1089/cap.2018.0006
- Wagner-Schuman M, Froehlich T, Kottyan, L., Brinkman, W., Ramsey, L., & Epstein, J. Pharmacogenomics of methylphenidate side effects in children with attention-deficit/hyperactivity disorder (ADHD). Journal of the american academy of child and adolescent psychiatry. Conference: 64th annual meeting american academy of child and adolescent psychiatry, AACAP 2017. United states, 56(10), S289. Retrieved from http://cochranelibrary-wiley.com/o/cochrane/clcentral/articles/297/CN-01452297/frame.html doi:10.1016/j.jaac.2017.09.383
- <https://clinicaltrials.gov/ct2/show/NCT01727414>

*Authors provided us with pre-cross over data*

Includes also participants from **Epstein2011 (NCT01238822) (**previously excluded from Cortese et al., Lancet Psychiatry 2018):

**Epstein2011 (NCT01238822)**

- Epstein JN, Brinkman WB, Froehlich T, et al. Effects of stimulant medication, incentives, and event rate on reaction time variability in children with ADHD. Neuropsychopharmacology. Apr 2011;36(5):1060-1072
- Froehlich TE, Epstein JN, Nick TG, et al. Pharmacogenetic predictors of methylphenidate dose-response in attention-deficit/hyperactivity disorder*. J Am Acad Child Adolesc Psychiatry.* 2011;50(11):1129-1139 e1122.
- Froehlich TE, Antonini TN, Brinkman WB, et al. Mediators of methylphenidate effects on math performance in children with attention-deficit hyperactivity disorder. *J Dev Behav Pediatr.* 2014;35(2):100-107.
- Post hoc analysis on sleep parameters in: Becker SP, Froehlich TE, Epstein JN. Effects of Methylphenidate on Sleep Functioning in Children with Attention-Deficit/Hyperactivity Disorder. *J Dev Behav Pediatr*. 2016;37(5):395-404.
- Orban SA, Karamchandani TA, Tamm L, Sidol CA, Peugh J, Froehlich TE, Brinkman WB, Estell N, Mii AE, Epstein JN. Attention-Deficit/HyperactivityDisorder-Related Deficits and Psychostimulant Medication Effects on Comprehensionof Audiovisually Presented Educational Material in Children. J Child Adolesc Psychopharmacol. 2018. doi: 10.1089/cap.2018.0006

**21. Gau2007 , B4Z-TW-S010, NCT00485459**

- Gau SS, Huang YS, Soong WT, et al. A randomized, double-blind, placebo-controlled clinical trial on once-daily atomoxetine in Taiwanese children and adolescents with attention-deficit/hyperactivity disorder. *J Child Adolesc* *Psychopharmacol.* 2007;17(4):447-460.
- https://clinicaltrials.gov/ct2/show/NCT00485459
- https://assets.contentful.com/hadumfdtzsru/5VQbWaMDAswCWsK2OCki2c/bf817d7446e489369c57231d112c4c80/Atomoxetine-B4Z-TW-S010.pdf
- https://www.accessdata.fda.gov/drugsatfda_docs/nda/2002/21-411_Strattera.cfm

**22. Geller2007, B4Z-US-LYBP**

- Geller D, Donnelly C, Lopez F, et al. Atomoxetine treatment for pediatric patients with attentiondeficit/ hyperactivity disorder with comorbid anxiety disorder. *J Am Acad Child Adolesc Psychiatry.* 2007;46(9):1119-1127.
- Pooled in: Newcorn JH, Sutton VK, Zhang S, et al. Characteristics of placebo responders in pediatric clinical trials of attention-deficit/hyperactivity disorder. *J Am Acad Child Adolesc Psychiatry.* 2009;48(12):1165-1172.
- https://assets.contentful.com/hadumfdtzsru/6DvfHWkAakuaUkQgM6UGwS/b3c20317fc84b267cfb138cc326e8c21/Atomoxetine-B4Z-US-LYBP_a_.pdf
- https://www.accessdata.fda.gov/drugsatfda_docs/nda/2002/21-411_Strattera.cfm

**23. Greenhill2002**

- Greenhill LL, Findling RL, Swanson JM. A double-blind, placebo-controlled study of modified-release methylphenidate in children with attention-deficit/hyperactivity disorder. *Pediatrics.* 2002;109(3):E39.

**24. Greenhill2006a, Study 309 Cephalon**

- Greenhill, L.L., Biederman, J., Boellner, S.W., Rugino, T.A., Sangal, R.B., Swanson, J.M., 2005. Modafinil film coated tablets significantly improve symptoms on ADHD Rating Scale-IV School and Home and overall clinical condition in children and adolescents with attention-deficit/hyperactivity disorder. J. Child Adolesc. Psychopharmacol. 15 (6), 849e850. NCDEU 45th Annual Meeting Boca Raton, Florida, USA Session I-88
- Greenhill LL, Wigal SB, Kratochvil C, Boellner S. ADHD symptoms and behavior improved with modafinil pediatric formulation. *158th Annual Meeting of the American Psychiatric Association; 2005 May 21-26; Atlanta,* *GA 2005.*
- Greenhill LL, Biederman J, Boellner SW, et al. A randomized, double-blind, placebo-controlled study of modafinil film-coated tablets in children and adolescents with attention-deficit/hyperactivity disorder. *J Am Acad Child* *Adolesc Psychiatry.* 2006;45(5):503-511.
- Pooled in: Wigal SB, Biederman J, Swanson JM, Yang R, Greenhill LL. Efficacy and safety of modafinil filmcoated tablets in children and adolescents with or without prior stimulant treatment for attentiondeficit/hyperactivity disorder: pooled analysis of 3 randomized, double-blind, placebo-controlled studies. *Prim Care* *Companion J Clin Psychiatry.* 2006;8(6):352-360.
- Pooled in: Biederman J, Pliszka SR. Modafinil improves symptoms of attention-deficit/hyperactivity disorder across subtypes in children and adolescents. *J Pediatr.* 2008;152(3):394-399.
- https://www.accessdata.fda.gov/drugsatfda_docs/nda/98/020717A_Provigil.cfm
- Modafinil (CEP-1538) Tablets, Supplemental NDA 20-717/S-019, ADHD Indication Briefing Document for Psychopharmacologic Drugs Advisory Committee Meeting (Document provided by Dr Eric Konofal)

**25. Greenhill2006b , CRIT124E2301**

- Greenhill LL, Muniz R, Ball RR, Levine A, Pestreich L, Jiang H. Efficacy and safety of dexmethylphenidate extended-release capsules in children with attention-deficit/hyperactivity disorder. *J Am Acad Child Adolesc* *Psychiatry.* 2006;45(7):817-823.
- https://www.novctrd.com/CtrdWeb/displaypdf.nov?trialresultid=1622 (ID: CRIT124E2301)
- <https://www.accessdata.fda.gov/drugsatfda_docs/nda/2005/021802s000TOC.cfm>

**26. Griffiths2018, ACTRN 12607000535471**

- Protcol: Tsang TW, Kohn MR, Hermens DF, et al. A randomized controlled trial investigation of a non-stimulant in attention deficit hyperactivity disorder (ACTION): rationale and design. *Trials.* 2011;12:77.
- Tsang TW, Kohn MR, Clarke SD, Williams LM. Cognition and emotion in child and adolescent ADHD. *Biol Psychiatry.* 2012;71(8):74S.
- Tsang TW, Kohn MR, Clarke SD, Williams LM. Cognitive and emotion predictors of response to atomoxetine in children and adolescents with attention deficit hyperactivity disorder, with and without comorbid anxiety. *Biol* *Psychiatry.* 2013;73(9):47S.
- Kohn MR, Griffiths KR, Clarke S, et al. Pharmacological mediation of cognition in children and adolescents presenting with cross-disorder symptoms of adhd and anxiety. *Biol Psychiatry.* 2015;77(9): 119S
- Griffiths KR, Leikauf JE, Tsang TW, Clarke S, Hermens DF, Efron D, Williams LM, Kohn MR. Response inhibition and emotional cognition improved by atomoxetine in children and adolescents with ADHD: The ACTION randomized controlled trial. J Psychiatr Res. 2018;102:57-64.

**27. Harfterkamp2012, NCT00380692**

- Harfterkamp M, van de Loo-Neus G, Minderaa RB, et al. A randomized double-blind study of atomoxetine versus placebo for attention-deficit/hyperactivity disorder symptoms in children with autism spectrum disorder. *J Am Acad* *Child Adolesc Psychiatry.* 2012;51(7):733-741.
- van der Meer JM, Harfterkamp M, van de Loo-Neus G, et al. A randomized, double-blind comparison of atomoxetine and placebo on response inhibition and interference control in children and adolescents with autism spectrum disorder and comorbid attention-deficit/hyperactivity disorder symptoms. *J Clin Psychopharmacol.* 2013;33(6):824-827.
- Follow-up in: Harfterkamp M, Buitelaar JK, Minderaa RB, van de Loo-Neus G, van der Gaag RJ, Hoekstra PJ. Long-term treatment with atomoxetine for attention-deficit/hyperactivity disorder symptoms in children and adolescents with autism spectrum disorder: an open-label extension study. *J Child Adolesc Psychopharmacol.* 2013;23(3):194-199.
- Harfterkamp M, Van Der Meer J. A Randomized double-blind study of atomoxetine vs. placebo followed by an open label extension period of treatment with atomoxetine for ADHD symptoms in children with ASD. *Eur Child* *Adolesc Psychiatry.* 2013;1):S216-S217.
- Harfterkamp M, Buitelaar JK, Minderaa RB, van de Loo-Neus G, van der Gaag RJ, Hoekstra PJ. Atomoxetine in autism spectrum disorder: no effects on social functioning; some beneficial effects on stereotyped behaviors, inappropriate speech, and fear of change. *J Child Adolesc Psychopharmacol.* 2014;24(9):481-485.
- Harfterkamp M, van der Meer D, van der Loo-Neus G, Buitelaar JK, Minderaa RB, Hoekstra PJ. No evidence for predictors of response to atomoxetine treatment of attention-deficit/hyperactivity disorder symptoms in children and adolescents with autism spectrum disorder. *J Child Adolesc Psychopharmacol.* 2015;25(4):372-375.
- <https://clinicaltrials.gov/ct2/show/NCT00380692>

**28. Hervas2014 , SPD503-316, NCT01244490, EudraCT: 2010- 018579-12**

- Hervas A, Huss M, Johnson M, et al. Efficacy and safety of extended-release guanfacine hydrochloride in children and adolescents with attention-deficit/hyperactivity disorder: a randomized, controlled, phase III trial. *Eur Neuropsychopharmacol.* 2014;24(12):1861-1872.
- Hervas A, Huss M, Johnson M, et al. Guanfacine Xr (Gxr) for Children and Adolescents with Attention-Deficit/Hyperactivity Disorder (Adhd): Phase 3, Blind, Multicenter, Placebo- and Active-Reference Study. *Eur* *Psychiatry.* 2014;29.
- Pooled in: Huss M, Hervas A, Newcorn JH, et al. Guanfacine extended release for attention-deficit/hyperactivity disorder following inadequate response to prior methylphenidate. *Eur Neuropsychopharmacol.* 2014;24:S727-S728.
- Huss M, Hervas A, Johnson M, et al. Efficacy and Safety of Extended-Release Guanfacine Hydrochloride in Children and Adolescents with Attention-Deficit**/**Hyperactivity Disorder: A Randomized, Double-Blind, Multicentre, Placebo- and Active-Reference Phase 3 Study. *Aust N Z J Psychiatry.* 2015;49:111-111.
- Hervas A, Johnson M, McNicholas F, et al. Clinical global impressions-improvement scores by visit in a European, randomized, double-blind, placebo and active-controlled clinical trial of guanfacine extended release in children and adolescents with attention-deficit/hyperactivity disorder. *Eur Child Adolesc Psychiatry.* 2015;1):S138.
- Huss M, Johnson M, McNicholas F, et al. Attention-deficit/hyperactivity disorder rating scale IV subscale analysis by visit in a European, phase 3, randomized, double-blind clinical trial of guanfacine extended release in children and adolescents with attention-deficit/hyperactivity deficit. *Eur Child Adolesc Psychiatry.* 2015;1):S133-S134.
- Pooled in: Huss M, McBurnett K, Cutler A, Hervas A, Adeyi B, Dirks B. Clinical efficacy of guanfacine extended release among children with primarily inattentive subtype of Attention-Deficit/Hyperactivity Disorder: Results from four phase 3 studies. *ADHD Attention Deficit and Hyperactivity Disorders.* 2015;7:S44.
- Pooled in: Huss M, McBurnett K, Cutler AJ, et al. Separating efficacy and sedative effects of guanfacine extended release in children and adolescents with ADHD from four randomized, controlled, phase 3 clinical trials. *Eur* *Psychiatry.* 2016;33:S76-S77.
- Used for analysis in: Huss M, Sikirica V, Hervas A, Newcorn JH, Harpin V, Robertson B. Guanfacine extended release for children and adolescents with attention-deficit/hyperactivity disorder: efficacy following prior methylphenidate treatment. *Neuropsychiatric Disease & Treatment.* 2016;112:1085-1101.
- Secondary analysis in: Huss M, Hervas A, Dirks B, Bliss C, Prochazka J, Cutler A. Guanfacine extended release for children and adolescents with attention-deficit/hyperactivity disorder: Efficacy following prior stimulant treatment. *Eur Neuropsychopharmacol.* 2016;26:S737.
- Extension (EUCTR2011-004668-31-GB)**:** Huss M, Dirks B, Gu J, Ramos-Quiroga JA. Long-term growth-related safety outcomes of guanfacine extended release in children and adolescents with ADHD. *Eur* *Neuropsychopharmacol. Conference: 29th european college of* neuropsychopharmacology *congress, ECNP 2016. Austria. Conference start: 20160917. Conference end: 20160920.* 2017;26:S735-s736
- *Pooled in:* Huss, M., Newcorn, J., Connor, D., Hervas, A., Werner-Kiechle, T., & Robertson, B. (2017). Efficacy of guanfacine extended release in children and adolescents with ADHD and comorbid oppositional defiant disorder. *European Neuropsychopharmacology, 27 (Supplement 4)*, S1114.
- Post hoc analyses in: Coghill DR, Joseph A, Sikirica V, Kosinski M, Bliss C, Huss M. Correlations Between Clinical Trial Outcomes Based on Symptoms, Functional Impairments, and Quality of Life in Children and Adolescents With ADHD. J Atten Disord. 2017:1087054717723984. doi: 10.1177/1087054717723984.
- <https://clinicaltrials.gov/ct2/show/NCT01244490>
- http://www.shiretrials.com/-/media/files/clinical%20trials/clinicaltrialsen/clinical%20study%20reports/shirespd503-316-clinical-study-report-redact.pdf
- https://www.clinicaltrialsregister.eu/ctr-search/search?query=eudract_number:2010-018579-12

**29. Ichikawa2020, Japic CTI-152770**

- Ichikawa H, Miyajima T, Yamashita Y, Fujiwara M, Fukushi A, Saito K. Phase II/III Study of Lisdexamfetamine Dimesylate in Japanese Pediatric Patients with Attention-Deficit/Hyperactivity Disorder. *J Child Adolesc Psychopharmacol*. 2020 Feb;30(1):21-31.

**30. Kahbazi2009**

- Akhondzadeh S, Amiri S, Mohammadi M-R. A clinical trial of modafinil treatment in children and adolescents with attention deficit hyperactivity disorder: a double-blind and randomized trial. *Br J Clin Pharmacol.* 2008;65(6):981.
- Kahbazi M, Ghoreishi A, Rahiminejad F, Mohammadi MR, Kamalipour A, Akhondzadeh S. A randomized, doubleblind and placebo-controlled trial of modafinil in children and adolescents with attention deficit and hyperactivity disorder. *Psychiatry Res.* 2009;168(3):234-237.

**31. Kelsey2004, B4Z-US-LYBG**

- Kelsey DK, Sumner CR, Sutton V, et al. Once-Daily Atomoxetine in Childhood ADHD: Continuous Symptom Relief. *156th Annual Meeting of the American Psychiatric Association, May 17-22, San Francisco CA2003:Nr716.*
- Kelsey DK, Sumner CR, Casat CD, et al. Once-daily atomoxetine treatment for children with attentiondeficit/hyperactivity disorder, including an assessment of evening and morning behavior: a double-blind, placebocontrolled trial. *Pediatrics.* 2004;114(1):e1-8.
- Pooled in: Perwien AR, Faries DE, Kratochvil CJ, Sumner CR, Kelsey DK, Allen AJ. Improvement in health-related quality of life in children with ADHD: an analysis of placebo controlled studies of atomoxetine. *J Dev Behav* *Pediatr.* 2004;25(4):264-271.
- Pooled in: Biederman J, Spencer TJ, Newcorn JH, et al. Effect of comorbid symptoms of oppositional defiant disorder on responses to atomoxetine in children with ADHD: a meta-analysis of controlled clinical trial data. *Psychopharmacology (Berl)*. 2007;190:31-41
- Previous reference related to: Biederman J, Spencer T, Newcorn J, Gao H, Milton D, Feldman P. Does the Presence of Comorbid ODD Affect Responses to Atomoxetine? 158th Annual Meeting of the American Psychiatric Association; 2005 May 21-26; Atlanta, GA2005.
- Pooled in: Newcorn JH, Sutton VK, Weiss MD, Sumner CR. Clinical responses to atomoxetine in attentiondeficit/hyperactivity disorder: the Integrated Data Exploratory Analysis (IDEA) study. *J Am Acad Child Adolesc* *Psychiatry.* 2009;48(5):511-518.
- Pooled in: Newcorn JH, Sutton VK, Zhang S, et al. Characteristics of placebo responders in pediatric clinical trials of attention-deficit/hyperactivity disorder. *J Am Acad Child Adolesc Psychiatry.* 2009;48(12):1165-1172.
- Pooled in: Adler LA, Wilens T, Zhang S, et al. Atomoxetine treatment outcomes in adolescents and young adults with attention-deficit/hyperactivity disorder: results from a post hoc, pooled analysis. *Clin Ther.* 2012;34(2):363-373.
- https://assets.contentful.com/hadumfdtzsru/6Paceu2aB2yO0YO4qI8gEI/946961353d42e70064bf5e491ee77748/Atomoxetine-B4Z-US-LYBG.pdf
- https://www.accessdata.fda.gov/drugsatfda_docs/nda/2002/21-411_Strattera.cfm

**32. Kollins2011, SPD503-206, NCT00150592**

- Kollins SH, Wigal T, Vince B, et al. Guanfacine extended release in ADHD: Safety and sleep effects. *Proceedings of the Meeting of the American Academy of Child and Adolescent 2007; 23-28, Boston, MA*.
- Turnbow J, Kollins S, Lopez F, Lyne A, Youcha S, Rubin J. Response to Guanfacine Extended Release in Children and Adolescents with Attention-Deficit/Hyperactivity Disorder (ADHD). *Biol Psychiatry.* 2010;67(9, Suppl. S):218S.
- Kollins SH, Lopez FA, Vince BD, et al. Psychomotor functioning and alertness with guanfacine extended release in subjects with attention-deficit/hyperactivity disorder. *J Child Adolesc Psychopharmacol.* 2011;21(2):111-120.
- Pooled in Faraone SV, Glatt SJ. Effects of extended-release guanfacine on ADHD symptoms and sedation-related adverse events in children with ADHD. J Atten Disord. 2010;13(5):532-8.
- https://clinicaltrials.gov/ct2/show/results/NCT00150592 (additional ID:SPD503-206)

**33. Martenyi2010, B4Z-MW-LYCZ, NCT00386581**

- Martenyi F, Zavadenko N, Jarkova N. Atomoxetine in the treatment of psychostimulant-naive children and adolescents with ADHD: a 6-week, randomized, placebo-controlled trial in Russia. *Eur Neuropsychopharmacol.* 2006;16(Suppl. 4):S527-S528.
- Martenyi F, Zavadenko NN, Jarkova NB, et al. Atomoxetine in children and adolescents with attentiondeficit/hyperactivity disorder: a 6-week, randomized, placebo-controlled, double-blind trial in Russia. *Eur Child* *Adolesc Psychiatry.* 2010;19(1):57-66.
- https://clinicaltrials.gov/ct2/show/NCT00386581
- https://assets.contentful.com/hadumfdtzsru/6LOREfRmGAiCwAKSsokise/35fe9f24fed03897e6d6756c85e13670/Atomoxetine-B4Z-MW-LYCZ.pdf

**34. Mattingly2020, NCT03325881**

- Mattingly G, Arnold V, Yan B, Yu M, Robertson B. A Phase 3, Randomized Double-Blind Study of the Efficacy and Safety of Low-Dose SHP465 Mixed Amphetamine Salts Extended-Release in Children with Attention-Deficit/Hyperactivity Disorder. J Child Adolesc Psychopharmacol. 2020 Nov;30(9):549-557.

**35. Michelson2001, B4Z-MC-LYAC**

- Michelson D, Faries D, Wernicke J, et al. Atomoxetine in the treatment of children and adolescents with attentiondeficit/hyperactivity disorder: a randomized, placebo-controlled, dose-response study. *Pediatrics.* Nov 2001;108(5):E83.
- Pooled in: Dunn DW, Wernicke JF, Faries D, et al. Efficacy of atomoxetine in placebo-controlled pediatric attention-deficit hyperactivity disorder trials. *Ann Neurol.* 2001;50:S96-S.
- Pooled in: Allen AJ, Michelson D, Harder DR, Trapp NJ, Kelsey DK. Efficacy of atomoxetine in children and adolescents with ADHD. 155th Annual Meeting of the American Psychiatric Association; 2002 May 18-23; Philadelphia, PA2002:Nr348.
- Donnelly C, Faries D, Swensen A, et al. The effect of atomoxetine on the social and family functioning of children and adolescents with attention-deficit/hyperactivity disorder (ADHD). *Eur Neuropsychopharmacol.* 2002:S437.
- Allen AJ, Milton DR, Michelson D, Kelsey DK. Efficacy of atomoxetine treatment for adolescents with ADHD. 156th Annual Meeting of the American Psychiatric Association; 2003 May 17-22; San Francisco, CA2003:Nr643.
- Matza LS, Rentz AM, Secnik K, et al. The link between health-related quality of life and clinical symptoms among children with attention-deficit hyperactivity disorder. *J Dev Behav Pediatr.* 2004;25(3):166-174.
- Pooled in: Perwien AR, Faries DE, Kratochvil CJ, Sumner CR, Kelsey DK, Allen AJ. Improvement in health-related quality of life in children with ADHD: an analysis of placebo controlled studies of atomoxetine. *J Dev Behav* *Pediatr.* 2004;25(4):264-271.
- Newcorn JH, Spencer TJ, Biederman J, Milton DR, Michelson D. Atomoxetine treatment in children and adolescents with attention-deficit/hyperactivity disorder and comorbid oppositional defiant disorder. *J Am Acad* *Child Adolesc Psychiatry*. 2005;44(3):240-248.
- Pooled in: Michelson D, Read HA, Ruff DD, Witcher J, Zhang S, McCracken J. CYP2D60 and Clinical Response to Atomoxetine in Children and Adolescents with ADHD. *J Am Acad Child Adolesc Psychiatry*. 2007 2012-12-14 2007;46(2):242-251.
- Pooled in: Newcorn JH, Sutton VK, Weiss MD, Sumner CR. Clinical responses to atomoxetine in attentiondeficit/ hyperactivity disorder: the Integrated Data Exploratory Analysis (IDEA) study. *J Am Acad Child Adolesc* *Psychiatry.* 2009;48(5):511-518.
- Pooled in: Newcorn JH, Sutton VK, Zhang S, et al. Characteristics of placebo responders in pediatric clinical trials of attention-deficit/hyperactivity disorder. *J Am Acad Child Adolesc Psychiatry*. 2009;48(12):1165-1172.
- Pooled in: Block SL, Williams D, Donnelly CL, Dunn DW, Saylor KE, Ruberg SJ. Post hoc analysis: early changes in ADHD-RS items predict longer term response to atomoxetine in pediatric patients. *Clin Pediatr (Phila).* 2010;49(8):768-776.
- Pooled in: Adler LA, Wilens T, Zhang S, et al. Atomoxetine treatment outcomes in adolescents and young adults with attention-deficit/hyperactivity disorder: results from a post hoc, pooled analysis. *Clin Ther*. 2012;34(2):363-373.
- https://assets.contentful.com/hadumfdtzsru/E5UgeEzzbwqGagscMqAIC/4ec70be50f8aff4b6daac82c2e67e98c/Atomoxetine-B4Z-MC-LYAC.pdf
- https://www.accessdata.fda.gov/drugsatfda_docs/nda/2002/21-411_Strattera.cfm

**36. Michelson2002, B4Z-MC-LYAT**

- Michelson D, Allen AJ, Busner J, et al. Once-daily atomoxetine treatment for children and adolescents with attention deficit hyperactivity disorder: a randomized, placebo-controlled study. *Am J Psychiatry*. 2002;159(11):1896-1901.
- Pooled in: Allen AJ, Michelson D, Harder DR, Trapp NJ, Kelsey DK. Efficacy of atomoxetine in children and adolescents with ADHD. 155th Annual Meeting of the American Psychiatric Association; 2002 May 18-23; Philadelphia, PA2002:Nr348.
- Michelson D. Once-daily administration of atomoxetine: a new treatment for adhd. *155th Annual Meeting of the American Psychiatric Association.* 2002.
- Pooled in: Allen AJ, Milton DR, Michelson D, Kelsey DK. Efficacy of atomoxetine treatment for adolescents with ADHD. 156th Annual Meeting of the American Psychiatric Association; 2003 May 17-22; San Francisco, CA2003:Nr643
- Bukstein OG Once-daily atomoxetine may reduce attention deficit hyperactivity disorder symptoms in children and adolescents. *Evid Based Ment Health*. 2003; 6, 42. (Commentary)
- Pooled in: Biederman J, Gao H, Rogers AK, Spencer TJ. Comparison of parent and teacher reports of attentiondeficit/hyperactivity disorder symptoms from two placebo-controlled studies of atomoxetine in children. *Biol* *Psychiatry.* 2006;60(10):1106-1110.
- Pooled in: Biederman J, Spencer TJ, Newcorn JH, et al. Effect of comorbid symptoms of oppositional defiant disorder on responses to atomoxetine in children with ADHD: a meta-analysis of controlled clinical trial data. *Psychopharmacology* (Berl). 2007;190:31-41.
- Previous reference related to: Biederman J, Spencer T, Newcorn J, Gao H, Milton D, Feldman P. Does the Presence of Comorbid ODD Affect Responses to Atomoxetine? 158th Annual Meeting of the American Psychiatric Association; 2005 May 21-26; Atlanta, GA2005.
- Pooled in: Michelson D, Read HA, Ruff DD, Witcher J, Zhang S, McCracken J. CYP2D60 and Clinical Response to Atomoxetine in Children and Adolescents with ADHD. *J Am Acad Child Adolesc Psychiatry.* 2007;46(2):242-251. (B4Z-MC-LYAI(4331); NCT00190684)
- Pooled in: Newcorn JH, Sutton VK, Weiss MD, Sumner CR. Clinical responses to atomoxetine in attentiondeficit/hyperactivity disorder: the Integrated Data Exploratory Analysis (IDEA) study. *J Am Acad Child Adolesc* *Psychiatry.* 2009;48(5):511-518.
- Pooled in: Newcorn JH, Sutton VK, Zhang S, et al. Characteristics of placebo responders in pediatric clinical trials of attention-deficit/hyperactivity disorder. *J Am Acad Child Adolesc Psychiatry.* 2009;48(12):1165-1172.
- Pooled in: Block SL, Williams D, Donnelly CL, Dunn DW, Saylor KE, Ruberg SJ. Post hoc analysis: early changes in ADHD-RS items predict longer term response to atomoxetine in pediatric patients. *Clin Pediatr (Phila).* 2010;49(8):768-776.
- Pooled in: Adler LA, Wilens T, Zhang S, et al. Atomoxetine treatment outcomes in adolescents and young adults with attention-deficit/hyperactivity disorder: results from a post hoc, pooled analysis. *Clin Ther.* 2012;34(2):363-373.
- https://assets.contentful.com/hadumfdtzsru/6iueDKScs8sa0SO0y8IOCA/8cdf3a662993055780f76a30fd07fd30/Atomoxetine-B4Z-MC-LYAT.pdf
- https://www.accessdata.fda.gov/drugsatfda_docs/nda/2002/21-411_Strattera.cfm

**37. Montoya2009, B4Z-XM-LYDM, NCT00191945**

- Montoya A, Hervas A, Cardo E, et al. Evaluation of atomoxetine for first-line treatment of newly diagnosed, treatment-naive children and adolescents with attention deficit/hyperactivity disorder. *Curr Med Res Opin.* 2009;25(11):2745-2754.
- Subsample analysed in: Montoya A, Escobar R, Garcia-Polavieja MJ, et al. Changes of urine dihydroxyphenylglycol to norepinephrine ratio in children with attention-deficit hyperactivity disorder (ADHD) treated with atomoxetine. *J* *Child Neurol*. 2011;26(1):31-36.
- Post hoc analysis in: Escobar R, Montoya A, Polavieja P, et al. Evaluation of patients' and parents' quality of life in a randomized placebo-controlled atomoxetine study in attention-deficit/hyperactivity disorder. *J Child Adolesc* *Psychopharmacol*. 2009;19(3):253-263.
- Study from previous reference pooled in: Montoya A, Quail D, Anand E, Cardo E, Alda JA, Escobar R. Prognostic factors of improvement in health-related quality of life in atomoxetine-treated children and adolescents with attention-deficit/hyperactivity disorder, based on a pooled analysis. *Atten Defic Hyperact Disord*. 2014;6(1):25-34).
- Previous reference related to Montoya A, Quail D, Anand E, Cardo E, Alda JA, Escobar R. Prognostic factors of improvement in health related quality of life in children and adolescents with attention deficit/hyperactivity disorder, after atomoxetine treatment. *Eur Psychiatry*. 2012;27.
- https://clinicaltrials.gov/ct2/show/NCT00191945 (other ID: B4Z-XM-LYDM**)**
- https://www.clinicaltrialsregister.eu/ctr-search/search?query=eudract_number:2004-004088-31

**38. NCT00936299**

- https://clinicaltrials.gov/ct2/show/NCT00936299

**39. Newcorn2008, B4Z-MC-LYBI**

- Michelson D. A double-blind placebo-controlled comparison of atomoxetine, OROS methylphenidate and placebo. Presented at: American Academy of Child and Adolescent Psychiatry Meeting, Washington, DC, October19-23,2004.
- From Gibson AP, Bettinger TL, Patel NC, Crismon ML. Atomoxetine versus stimulants for treatment of attention deficit/hyperactivity disorder. *Ann Pharmacother.* 2006;40(6):1134-1142.
- Newcorn JH, Michelson D, Kratochvil CJ, Allen AJ, Ruff DD, Moore RJ. Low-dose atomoxetine for maintenance treatment of attention-deficit/hyperactivity disorder. *Pediatrics.* 2006;118(6):e1701-1706.
- Study from previous reference pooled in: Adler LA, Wilens T, Zhang S, et al. Atomoxetine treatment outcomes in adolescents and young adults with attention-deficit/hyperactivity disorder: results from a post hoc, pooled analysis. *Clin Ther*. 2012;34(2):363-373.
- Newcorn JH, Kratochvil CJ, Allen AJ, et al. Atomoxetine and osmotically released methylphenidate for the treatment of attention deficit hyperactivity disorder: acute comparison and differential response. *Am J Psychiatry.* 2008;165(6):721-730.
- Newcorn JH, Kratochvil CJ, Allen AJ, et al. Osmotically released methylphenidate compared to atomoxetine for ADHD. The Brown University Child & Adolescent Psychopharmacology Update 2008; 10: 8:1.
- Pooled in: Newcorn JH, Sutton VK, Zhang S, et al. Characteristics of placebo responders in pediatric clinical trials of attention-deficit/hyperactivity disorder. *J Am Acad Child Adolesc Psychiatry.* 2009;48(12):1165-1172.
- Toplak ME. Osmotically released methylphenidate is more effective than atomoxetine in children and adolescents with ADHD. *Evid Based Ment Health.* 2009;12(1):19.
- https://assets.contentful.com/hadumfdtzsru/1kdmkwXhF0Qi4GoGcqwOEK/73114fca375740ef3010383870b7762e/Atomoxetine-B4Z-MC-LYBI.pdf

**40. Newcorn2013, SPD503-314, NCT00997984**

- Newcorn JH, Stein MA, Childress AC, et al. Randomized, double-blind trial of guanfacine extended release in children with attention-deficit/hyperactivity disorder: morning or evening administration*. J Am Acad Child Adolesc* *Psychiatry*. 2013;52(9):921-930.
- Pooled in: Huss M, Newcorn J, Connor D, Hervas A, Werner-Kiechle T., Robertson B. (2017). Efficacy of guanfacine extended release in children and adolescents with ADHD and comorbid oppositional defiant disorder. *European Neuropsychopharmacology 2017: 27 (Supplement 4)*, S1114.
- Young J, Rugino T, Dammerman R, Lyne A, Newcorn JH. Efficacy of guanfacine extended release assessed during the morning, afternoon, and evening using a modified Conners' Parent Rating Scale-revised: Short Form. *J Child* *Adolesc Psychopharmacol*. 2014;24(8):435-441.
- Stein MA, Sikirica V, Weiss MD, Robertson B, Lyne A, Newcorn JH. Does Guanfacine Extended Release Impact Functional Impairment in Children with Attention-Deficit/Hyperactivity Disorder? Results from a Randomized Controlled Trial. *CNS Drugs*. 2015;29(11):953-962.
- https://clinicaltrials.gov/ct2/show/NCT00997984 (other ID: SPD503-314)

**41. Pliszka2000**

- Pliszka SR, Wynne SK, Olvera RL, Browne RG. Comparing adderall methylphenidate in ADHD. *152nd Annual Meeting of the American Psychiatric Association; 1999 May 15-20; Washington, DC.* 1999:Nr595.
- Pliszka SR, Browne RG, Olvera RL, Wynne SK. A double-blind, placebo-controlled study of Adderall and methylphenidate in the treatment of attention-deficit/hyperactivity disorder. *J Am Acad Child Adolesc Psychiatry*.

2000;39(5):619-626.

- Faraone SV, Pliszka SR, Olvera RL, Skolnik R, Biederman J. Efficacy of Adderall and methylphenidate in attention deficit hyperactivity disorder: a reanalysis using drug-placebo and drug-drug response curve methodology*. J Child* *Adolesc Psychopharmacol*. 2001;11(2):171-180.
- Faraone SV, Pliszka SR, Olvera RL, Biederman J. Adderall and methylphenidate in ADHD*. Annual Meeting of the American Psychiatric Association, 2001.*

**42. Pliszka2017, NCT02520388**

- Pliszka SR. A phase 3 registration trial of delayed-release and extended-release methylphenidate (HLD200) in the treatment of early morning functioning impairments in children with attention deficit/ hyperactivity disorder. *J Am Acad Child Adolesc Psychiatry.* 2016;55 (10 Supplement 1):S315.
- Arnold VK, DeSousa NJ, Incledon B, et al. Pivotal phase 3 trial evaluating the efficacy and safety of HLD200, a novel delayed-release and extended-release formulation of methylphenidate, in children with attention-deficit/hyperactivity disorder. *J Am Acad Child Adolesc Psychiatry.* 2016;55 (10 Supplement 1):S170.
- Wilens T, Faraone S, Hammerness P, Pliszka S, Sousa N, Sallee F, Incledon B, Newcorn J. Clinically meaningful improvements with DR/ER-MPH in at-home functional impairment during the early morning, late afternoon, and evening in children with attention-deficit/hyperactivity disorder (ADHD). Journal of the american academy of child and adolescent psychiatry. Conference: 64th annual meeting american academy of child and adolescent psychiatry, AACAP 2017. United states, 56(10), S170. Retrieved from http://cochranelibrary-wiley.com/o/cochrane/clcentral/articles/309/CN-01452309/frame.html doi:10.1016/j.jaac.2017.09.067
- Pliszka SR, Wilens TE, Bostrom S, Arnold VK, Marraffino A, Cutler AJ, López FA, DeSousa NJ, Sallee FR, Incledon B, Newcorn JH. Efficacy and Safety of HLD200, Delayed-Release and Extended-Release Methylphenidate, in Children with Attention-Deficit/Hyperactivity Disorder. *J Child Adolesc Psychopharmacol*. 2017;27(6):474-482.
- Pliszka S, Arnold V, Marraffino A, Desousa N, Incledon B, Sallee F, Wilens T Newcorn J. Efficacy and safety of HLD200, a novel delayed-release and extended-release methylphenidate formulation, in children with Attention-Deficit/Hyperactivity Disorder: Results from a pivotal phase 3 trial. *Atten Defic Hyperact Disord* 2017*; 9 (1 Supplement)*: S39. doi:http://dx.doi.org/10.1007/s12402-017-0224-y
- Pliszka S, Arnold V, Marraffino A, DeSousa N, Incledon, B., Sallee, F., Wilens TE, Newcorn J. Effect of Delayed-Release and Extended-Release Methylphenidate (DR/ER-MPH) on All-Day and Early Morning ADHD-Related Symptoms: analysis of ADHD-Rating Scale-IV and ADHD-AM-Rating Scale Items From a Phase 3 Trial. Journal of the American Academy of Child and Adolescent Psychiatry 2018, 57(10), S169‐. Retrieved from https://www.cochranelibrary.com/central/doi/10.1002/central/CN-01654958/full

https://www.sciencedirect.com/science/article/abs/pii/S0890856718314667?via%3Dihub doi:10.1016/j.jaac.2018.09.119

- Wilens T, Pliszka S, Arnold V, Marraffino A, DeSousa N, Incledon B, Randy, S, Newcorn J. Consistent efficacy of DR/ER-MPH on early morning functioning in children with ADHD: analysis of BSFQ item ratings from a pivotal phase 3 trial. CNS spectrums 2018, 23(1), 82‐. Retrieved from <https://www.cochranelibrary.com/central/doi/10.1002/central/CN-01915482/full>, https://www.cambridge.org/core/services/aop-cambridge-core/content/view/81641E30E65EED0A8094C7E12E1A962F/S1092852918000275a.pdf/div-class-title-130-consistent-efficacy-of-dr-er-mph-on-early-morning-functioning-in-children-with-adhd-analysis-of-bsfq-item-ratings-from-a-pivotal-phase-3-trial-div.pdf doi:10.1017/S1092852918000275
- <https://clinicaltrials.gov/ct2/show/record/NCT02520388>

*Note: this study was considered as excluded in Cortese et al. 2018 as that participants who entered the study were deemed to be responders to MPH; however, in the process of the present update, it appered clear that they were (at least) partially reponders. As such, consistently with other studies in cluded in Cortes et al., 2018, this study was included*

**43. Riggs2011, NCT00264797**

- Riggs PD, Winhusen T, Davies R, Leimberger J, Mikulich-Gilbertson SK. Los Angeles, CA: American Academy of Addiction Psychiatry Annual Meeting; 2009. A randomized controlled trial of OROS-MPH for ADHD in adolescents with substance use disorders. Los Angeles, CA: American Academy of Addiction Psychiatry Annual Meeting; 2009.
- Riggs PD, Winhusen T, Davies RD, et al. Randomized controlled trial of osmotic-release methylphenidate with cognitive-behavioral therapy in adolescents with attention-deficit/hyperactivity disorder and substance use disorders. *J Am Acad Child Adolesc Psychiatry.* 2011;50(9):903-914.
- Gray KM, Riggs PD, Min SJ, Mikulich-Gilbertson SK, Bandyopadhyay D, Winhusen T. Cigarette and cannabis use trajectories among adolescents in treatment for attention-deficit/hyperactivity disorder and substance use disorders. *Drug Alcohol Depend.* 2011;117(2-3):242-247.
- Pooled in Inhusen TM, Lewis DF, Riggs PD, et al. Subjective effects, misuse, and adverse effects of osmotic-release methylphenidate treatment in adolescent substance abusers with attention-deficit/hyperactivity disorder. *J Child* *Adolesc Psychopharmacol.* 2011;21(5):455-463.
- Tamm L, Adinoff B, Nakonezny PA, Winhusen T, Riggs P. Attention-deficit/hyperactivity disorder subtypes in adolescents with comorbid substance-use disorder. *Am J Drug Alcohol Abuse.* 2012;38(1):93-100.
- Warden D, Riggs PD, Min SJ, et al. Major depression and treatment response in adolescents with ADHD and substance use disorder. *Drug Alcohol Depend.* 1 2012;120(1-3):214-219.
- Tamm L, Trello-Rishel K, Riggs P, et al. Predictors of treatment response in adolescents with comorbid substance use disorder and attention-deficit/hyperactivity disorder. *J Subst Abuse Treat.* 2013;44(2):224-230.
- McPherson S, Mamey MR, Barbosa-Leiker C, Murphy SM, Roll J. Osmotic-release methylphenidate randomized controlled trial for adolescents with attention-deficit/hyperactivity disorders and substance use disorders: A missing data sensitivity analysis. *Drug Alcohol Depend*. 2015;146:e36.

**44. Rugino2003**

- Rugino TA, Samsock TC, Adkins L. Modafinil in children with ADHD: a double-blind, placebo-controlled study. *155th Annual Meeting of the American Psychiatric Association; 2002 May 18-23; Philadelphia, PA.* 2002:No. 77.
- Rugino TA, Samsock TC. Modafinil in children with attention-deficit hyperactivity disorder. *Pediatr Neurol.* 2003;29(2):136-142.

**45. Rugino2018, NCT01156051**

- Rugino TA. Effect on Primary Sleep Disorders When Children With ADHD Are Administered Guanfacine Extended Release. *J Atten Disord.* 2018. DOI: 10.1177/1087054714554932.
- <https://clinicaltrials.gov/ct2/show/NCT01156051>

**46. Sallee2009, SPD503-304, NCT00150618**

- Biederman J, Lyne A, Sallee FR, et al. Efficacy and Safety of Guanfacine Extended Release in Attention-Deficit/ Hyperactivity Disorder. *Presented at the 54th Annual Meeting of the American Academy of Child & Adolescent* *Psychiatry; 23 - 28 October 2007 Boston, Massachusetts*
- Sallee FR, McGough J, Wigal T, Donahue J, Lyne A, Biederman J. Guanfacine extended release in children and adolescents with attention-deficit/hyperactivity disorder: a placebo-controlled trial. *J Am Acad Child Adolesc* *Psychiatry.* 2009;48(2):155-165.
- Pooled in: Sallee FR, Lyne A, Wigal T, McGough JJ. Long-term safety and efficacy of guanfacine extended release in children and adolescents with attention-deficit/hyperactivity disorder. *J Child Adolesc Psychopharmacol.* 2009;19(3):215-226. (open label extension plus another open label study)
- Related to previous reference: Sallee F, McGough JJ, Wigall T, Farrand K, Lyne A, Biederman J. Long-term safety and efficacy of guanfacine extended release in children and adolescents with Attention-Deficit/Hyperactivity disorder. *Biol Psychiatry.* 2008;63:247S.
- Pooled in: Faraone SV, Glatt SJ. Effects of extended-release guanfacine on ADHD symptoms and sedation-related adverse events in children with ADHD. *J Atten Disord.* 2010;13(5):532-538.
- Pooled in: Sallee FR, Kollins SH, Wigal TL. Efficacy of guanfacine extended release in the treatment of combined and inattentive only subtypes of attention-deficit/hyperactivity disorder. *J Child Adolesc Psychopharmacol.* 2012;22(3):206-214.
- Pooled in: Huss M, McBurnett K, Cutler AJ, Hervás A, Bliss C, Gao J, Dirks B, Newcorn JH. Distinguishing the efficacy and sedative effects of guanfacine extended release in children and adolescents with attention-deficit/hyperactivity disorder. Eur Neuropsychopharmacol. 2019;29(3):432-443.
- https://www.clinicaltrials.gov/ct2/show/NCT00150618 (other ID: SPD503-304)
- https://www.accessdata.fda.gov/drugsatfda_docs/nda/2009/022037_intuniv_toc.cfm
- http://www.shirecanada.com/-/media/shire/shireglobal/shirecanada/pdffiles/product%20information/intuniv-xr-pmen.pdf

**47. SPD489-405, NCT01552915**

- Newcorn JH, Nagy P, Childress AC, Frick G, Yan B, Pliszka S. Randomized, Double-Blind, Placebo-Controlled Acute Comparator Trials of Lisdexamfetamine and Extended-Release Methylphenidate in Adolescents With Attention-Deficit/Hyperactivity Disorder. CNS Drugs. 2017;31(11):999-1014.
- Newcorn J, Nagy P, Childress A, et al. Randomized, double-blind, active- and placebocontrolled trials of lisdexamfetamine dimesylate in adolescents with Attention-Deficit/Hyperactivity Disorder. *ADHD. Atten Defic Hyperact Disord.* 2015;7:S47.
- Analysed in: Coghill D, Nagy P, Frick G, Naser N, Wu J, Newcorn J. Comparing the time course of efficacy of lisdexamfetamine dimesylate and osmotic controlled-release methylphenidate in children and adolescents with ADHD. *Aust N Z J Psychiatry.* 2016; 50, 114‐115. Retrieved from https://www.cochranelibrary.com/central/doi/10.1002/central/CN-01719602/full doi:10.1177/0004867416640967
- <https://clinicaltrials.gov/ct2/show/NCT01552915>

**48. SPD489-406, NCT01552902**

- <https://clinicaltrials.gov/ct2/show/NCT01552902>
- Analysed in: Coghill D, Nagy P, Frick G, Naser N, Wu J, Newcorn J. Comparing the time course of efficacy of lisdexamfetamine dimesylate and osmotic controlled-release methylphenidate in children and adolescents with ADHD. *Aust N Z J Psychiatry.* 2016; 50, 114‐115. Retrieved from https://www.cochranelibrary.com/central/doi/10.1002/central/CN-01719602/full doi:10.1177/0004867416640967

**49, 50. Spencer2002 , B4Z-MC-HFBD, B4Z-MC-HFBK**

- Pooled in: Dunn DW, Wernicke JF, Faries D, et al. Efficacy of atomoxetine in placebo-controlled pediatric attention-deficit hyperactivity disorder trials. *Ann Neurol*. 2001;50:S96-S.
- Stein MA, Lewis J, Conlon C, et al. A comparison of tomoxetine hydrochloride and placebo in pediatric patients with Attention-Deficit/Hyperactivity Disorder. *Pediatr Res.* 2001;49(4 Part 2):451A.
- Spencer T, Heiligenstein JH, Biederman J, et al. Results from 2 proof-of-concept, placebo-controlled studies of atomoxetine in children with attention-deficit/hyperactivity disorder. *J Clin Psychiatry.* 2002;63(12):1140-1147.
- Data in girls only in: Biederman J, Heiligenstein JH, Faries DE, et al. Efficacy of atomoxetine versus placebo in school-age girls with attention-deficit/hyperactivity disorder. *Pediatrics.* 2002;110(6):e75.
- Subset analysis in: Kaplan S, Heiligenstein J, West S, et al. Efficacy and safety of atomoxetine in childhood attention-deficit/hyperactivity disorder with comorbid oppositional defiant disorder. *J Atten Disord.* 2004;8(2):45-52.
- Included in: Wernicke JF, Adler L, Spencer T, et al. Changes in symptoms and adverse events after discontinuation of atomoxetine in children and adults with attention deficit/hyperactivity disorder: a prospective, placebo-controlled assessment. *J Clin Psychopharmacol.* 2004;24(1):30-35.
- Pooled in: Michelson D, Read HA, Ruff DD, Witcher J, Zhang S, McCracken J. CYP2D60 and Clinical Response to Atomoxetine in Children and Adolescents with ADHD. *J Am Acad Child Adolesc Psychiatry.* 2007;46(2):242-251.
- Pooled in: Newcorn JH, Sutton VK, Weiss MD, Sumner CR. Clinical responses to atomoxetine in attentiondeficit/hyperactivity disorder: the Integrated Data Exploratory Analysis (IDEA) study. *J Am Acad Child Adolesc* *Psychiatry.* 2009;48(5):511-518.
- Pooled in: Newcorn JH, Sutton VK, Zhang S, et al. Characteristics of placebo responders in pediatric clinical trials of attention-deficit/hyperactivity disorder. *J Am Acad Child Adolesc Psychiatry.* 2009;48(12):1165-1172.
- Pooled in: Block SL, Williams D, Donnelly CL, Dunn DW, Saylor KE, Ruberg SJ. Post hoc analysis: early changes in ADHD-RS items predict longer term response to atomoxetine in pediatric patients. *Clin Pediatr (Phila).* 2010;49(8):768-776.
- Pooled in: Adler LA, Wilens T, Zhang S, et al. Atomoxetine treatment outcomes in adolescents and young adults with attention-deficit/hyperactivity disorder: results from a post hoc, pooled analysis. *Clin Ther.* 2012;34(2):363-373.
- https://assets.contentful.com/hadumfdtzsru/mPlwKp0FRQawsC8CU4KmW/5abf313ad3718e1aebc62b09682712fc/Atomoxetine-B4Z-MC-HFBD.pdf ;
- https://assets.contentful.com/hadumfdtzsru/665NUmKWsgCEmMAoKaEGgy/ce8a8ad38895ab7a3bd7ce5f0db7423b/Atomoxetine-B4Z-MC-HFBK.pdf
- https://www.accessdata.fda.gov/drugsatfda_docs/nda/2002/21-411_Strattera.cfm

**51. Spencer2006, SLI381-314, NCT00507065**

- Wilens TE, Spencer TJ, Biederman J, Weisler RH, Read SC, Partiot A. Mixed amphetamine salts XR: cardiovascular safety in adolescents with ADHD. *158th Annual Meeting of the American Psychiatric Association;* *2005 May 21-26; Atlanta, GA2005*
- Wilens TE, Spencer TJ, Biederman J. Short- and long-term cardiovascular effects of mixed amphetamine salts extended-release in adolescents with ADHD. *CNS Spectr.* 2005;10(10 Suppl 15):22-30.
- Spencer TJ, Wilens TE, Biederman J, Weisler RH, Read SC, Pratt R. Efficacy and safety of mixed amphetamine salts extended release (Adderall XR) in the management of attention-deficit/hyperactivity disorder in adolescent patients: a 4-week, randomized, double-blind, placebo-controlled, parallel-group study. *Clin Ther.* 2006;28(2):266-279.
- https://clinicaltrials.gov/ct2/show/NCT00507065 (additional ID: SLI381-314**)**

**52. Svanborg2009, B4Z-SO-LY15, EUCTR2004-003941-42-SE, NCT00191542**

- Svanborg P, Thernlund G, Gustafsson PA, Hagglof B, Poole L, Kadesjo B. Efficacy and safety of atomoxetine as add-on to psychoeducation in the treatment of attention deficit/hyperactivity disorder: A randomized, double-blind, placebo-controlled study in stimulant-naive Swedish children and adolescents. *Eur Child Adolesc Psychiatry.* 2009;18(4):240-249.
- Svanborg P, Thernlund G, Gustafsson PA, Hagglof B, Schacht A, Kadesjo B. Atomoxetine improves patient and family coping in attention deficit/hyperactivity disorder: a randomized, double-blind, placebo-controlled study in Swedish children and adolescents. *Eur Child Adolesc Psychiatry.* 2009;18(12):725-735.
- Myren KJ, Thernlund G, Nylen A, Schacht A, Svanborg P. Atomoxetine's effect on societal costs in Sweden. *J Atten Disord.* 2010;13(6):618-628.
- Pooled in: Montoya A, Quail D, Anand E, Cardo E, Alda JA, Escobar R. Prognostic factors of improvement in health-related quality of life in atomoxetine-treated children and adolescents with attention-deficit/hyperactivity disorder, based on a pooled analysis. *Atten Defic Hyperact Disord.* 2014;6(1):25-34.
- Previous reference Related to: Montoya A, Quail D, Anand E, Cardo E, Alda JA, Escobar R. Prognostic factors of improvement in health related quality of life in children and adolescents with attention deficit/hyperactivity disorder, after atomoxetine treatment. *Eur Psychiatry.* 2012;27
- https://www.clinicaltrialsregister.eu/ctr-search/search?query=eudract_number:2004-003941-42
- https://clinicaltrials.gov/ct2/show/NCT00191542 (other ID: B4Z-SO-LY15. 6671**)**

**53. Swanson2006**

- Biederman J, Wilens TE, Lopez FA. Modafinil pediatric formulation has early and sustained effect in ADHD. *158^th^ Annual Meeting of the American Psychiatric Association; 2005 May 21-26; Atlanta, GA2005.*
- Swanson JM, Greenhill LL, Lopez FA, et al. Modafinil film-coated tablets in children and adolescents with attention-deficit/hyperactivity disorder: results of a randomized, double-blind, placebo-controlled, fixed-dose study followed by abrupt discontinuation. *J Clin Psychiatry.* 2006;67(1):137-147.
- Pooled in: Wigal SB, Biederman J, Swanson JM, Yang R, Greenhill LL. Efficacy and safety of modafinil filmcoated tablets in children and adolescents with or without prior stimulant treatment for attentiondeficit/hyperactivity disorder: pooled analysis of 3 randomized, double-blind, placebo-controlled studies. *Prim Care* *Companion J Clin Psychiatry.* 2006;8(6):352-360.
- Pooled in: Biederman J, Pliszka SR. Modafinil improves symptoms of attention-deficit/hyperactivity disorder across subtypes in children and adolescents. *J Pediatr.* 2008;152(3):394-399.
- https://www.accessdata.fda.gov/drugsatfda_docs/nda/98/020717A_Provigil.cfm
- Modafinil (CEP-1538) Tablets, Supplemental NDA 20-717/S-019, ADHD Indication Briefing Document for Psychopharmacologic Drugs Advisory Committee Meeting (Document provided by Dr Eric Konofal)

**54. Takahashi2009, B4Z-JE-LYBC, NCT00191295**

- Takahashi M, Takita Y, Yamazaki K, et al. A randomized, double-blind, placebo-controlled study of atomoxetine in Japanese children and adolescents with attention-deficit/hyperactivity disorder. *J Child Adolesc Psychopharmacol.* 2009;19(4):341-350.
- https://assets.contentful.com/hadumfdtzsru/4Twp3uVNc4a6eIMiGmAWKI/93fcf81d15873e185bd5eeba6f32aeb5/Atomoxetine-B4Z-JE-LYBC.pdf
- https://www.accessdata.fda.gov/drugsatfda_docs/nda/2002/21-411_Strattera.cfm
- <https://clinicaltrials.gov/ct2/show/NCT00191295>

**55. Wehmeier2012, B4Z-SB-LYDV, NCT00546910**

- Wehmeier PM, Schacht A, Wolff C, Otto WR, Dittmann RW, Banaschewski T. Neuropsychological outcomes across the day in children with attention-deficit/hyperactivity disorder treated with atomoxetine: results from a placebo-controlled study using a computer-based continuous performance test combined with an infra-red motiontracking device. *J Child Adolesc Psychopharmacol*. 2011;21(5):433-444.
- Wehmeier PM, Schacht A, Ulberstad F, et al. Does atomoxetine improve executive function, inhibitory control, and hyperactivity? Results from a placebo-controlled trial using quantitative measurement technology. *J Clin* *Psychopharmacol.* 2012;32(5):653-660.
- Post hoc analysis in: Wehmeier PM, Kipp L, Banaschewski T, Dittmann RW, Schacht A. Does Comorbid Disruptive Behavior Modify the Effects of Atomoxetine on ADHD Symptoms as Measured by a Continuous Performance Test and a Motion Tracking Device? *J Atten Disord.* 2015;19(7):591-602
- Post hoc analysis in: Wehmeier PM, Dittmann RW, Banaschewski T, Schacht A. Does stimulant pretreatment modify atomoxetine effects on core symptoms of ADHD in children assessed by quantitative measurement technology? *J Atten Disord.* 2014;18(2):105-116.
- https://clinicaltrials.gov/ct2/show/NCT00546910 (additional ID: B4Z-SB-LYDV(11148)

**56. Wigal2004**

- Connors K, Casant C, Elia J, et al. Randomized Trial of d-MPH Focalin and d,1-MPH in Children with ADHD [abstract]. 155th Annual Meeting of the American Psychiatric Association; 2002 May 18-23; Philadelphia, PA2002
- Wigal S, Swanson JM, Feifel D, et al. A double-blind, placebo-controlled trial of dexmethylphenidate hydrochloride and d,l-threo-methylphenidate hydrochloride in children with attention-deficit/hyperactivity disorder. *J Am Acad* *Child Adolesc Psychiatry.* 2004;43(11):1406-1414.
- Post hoc analysis in: Weiss M, Wasdell M, Patin J. A post hoc analysis of d-threo-methylphenidate hydrochloride (focalin) versus d,l-threo-methylphenidate hydrochloride (ritalin). *J Am Acad Child Adolesc Psychiatry.* 2004;43(11):1415-1421.
- <https://www.accessdata.fda.gov/drugsatfda_docs/nda/2001/21-278_Focalin_medr_P2.pdf>

**57. Wigal2015, NCT01239030**

- Wigal SB, Nordbrock E, Adjei AL, Childress A, Kupper RJ, Greenhill L. Efficacy of Methylphenidate Hydrochloride Extended-Release Capsules (Aptensio XR™) in Children and Adolescents with Attention-Deficit/Hyperactivity Disorder: A Phase III, Randomized, Double-Blind Study. *CNS Drugs.* 2015;29(4):331-40. doi: 10.1007/s40263-015-0241-3.
- Wigal SB, Adjei AL, Childress A, Chang WW, Kupper RJ. A study of methylphenidate extended-release capsules in a randomized, double-blind, placebo-controlled protocol in children and adolescents with ADHD. *CNS* *spectrums. Conference: 2014 NEI psychopharmacology congress. United states. Conference start: 20141113.* *Conference end: 20141116.* 2017;20(1):67-68.
- Pooled in: Owens J, Weiss M, Nordbrock E, et al. Effect of Aptensio XR (methylphenidate HCl extended-release) capsules on sleep in children with attention-deficit/hyperactivity disorder. *J Child Adolesc Psychopharmacol.* 2016;26:873-881
- https://clinicaltrials.gov/ct2/show/NCT01239030 (other ID: RP-BP-EF002)
- Weiss M, Childress A, Mattingly G, Nordbrock E, Kupper RJ, Adjei AL. Relationship Between Symptomatic and Functional Improvement and Remission in a Treatment Response to Stimulant Trial. J Child Adolesc Psychopharmacol. 2018;28(8):521-529.
- Weiss M, Childress A, Nordbrock E, Adjei AL, Kupper RJ, Mattingly G. Characteristics of ADHD Symptom Response/Remission in a Clinical Trial of Methylphenidate Extended Release. J Clin Med. 2019;8(4). pii: E461. doi:10.3390/jcm8040461.

**58. Wilens2015, SPD503-312, EUCTR2011-002221-21, NCT01081132**

- Wilens TE, Robertson B, Sikirica V, et al. A Randomized, Placebo-Controlled Trial of Guanfacine Extended Release in Adolescents with Attention-Deficit/Hyperactivity Disorder. *J Am Acad Child Adolesc Psychiatry.* 2015;54(11):916-925.e912.
- Wilens TE, Harper L, Young JL, et al. Clinical response and symptomatic remission in adolescents with Attention-Deficit/Hyperactivity Disorder receiving guanfacine extended release in a phase 3 study. *ADHD Attention Deficit* *and Hyperactivity Disorders.* 2015;7:S98.
- Cutler A, Harper L, Young J, Adeyi B, Dirks B, Wilens T. Guanfacine extended release: Daytime sleepiness outcomes from a phase 3 clinical study in adolescents with attention-deficit/hyperactivity disorder. *Eur* *Neuropsychopharmacol.* 2015;25:S648-S649.
- Pooled in: Huss M, McBurnett K, Cutler A, Hervas A, Adeyi B, Dirks B. Clinical efficacy of guanfacine extended release among children with primarily inattentive subtype of Attention-Deficit/Hyperactivity Disorder: Results from four phase 3 studies. *Atten Defic Hyperact Disord.* 2015;7:S44.
- Pooled in: Huss M, McBurnett K, Cutler AJ, et al. Separating efficacy and sedative effects of guanfacine extended release in children and adolescents with ADHD from four randomized, controlled, phase 3 clinical trials. *Eur* *Psychiatry.* 2016;33:S76-S77.
- Secondary analysis in: Huss M, Hervas A, Dirks B, Bliss C, Prochazka J, Cutler A. Guanfacine extended release for children and adolescents with attention-deficit/hyperactivity disorder: Efficacy following prior stimulant treatment. *Eur Neuropsychopharmacol.* 2016;26:S737.
- Pooled in: Huss, M., Newcorn, J., Connor, D., Hervas, A., Werner-Kiechle, T., & Robertson, B. (2017). Efficacy of guanfacine extended release in children and adolescents with ADHD and comorbid oppositional defiant disorder. *European Neuropsychopharmacology, 27 (Supplement 4)*, S1114.
- https://clinicaltrials.gov/ct2/show/NCT01081132 **(**other ID: SPD503-312)
- https://www.clinicaltrialsregister.eu/ctr-search/search?query=eudract_number:2011-002221-21

**eExclusion reasons 2.** Studies/citations discarded after assessing their full text for review question 2, with reasons for exclusions.

**Review question 2: Is the use of common medications for ADHD associated with headache in children and adolescents?**

***This list includes also the international trial registries references that were excluded* *after assessing study inclusion/exclusion criteria.***

**A. From the list of eligible trials in the updated search (*n* = 90)**

**B. From the list of discarded trials in the updated search (*n* = 58)**

**A. From the list of eligible trials in the updated search**

**1. Abikoff2009**

- Abikoff H, Nissley-Tsiopinis J, Gallagher R, et al. Effects of MPH-OROS on the organizational, time management, and planning behaviors of children with ADHD. *J Am Acad Child Adolesc Psychiatry.* 2009;48(2):166-175.
- Anonymous. Concerta for organizational deficits in ADHD. *The Brown University Child and Adolescent Behavior Letter* 2009;25;3:2.

*Reason for exclusion: No usable safety data*

**2. Adler2008a, B4Z-MC-LYBV, NCT00190931**

- Levine L, Tamura RN, Kelsey DK, Schoepp DD, Allen AJ. Functional outcomes in adults with attentiondeficit/ hyperactivity disorder following treatment with atomoxetine vs. placebo. *Neuropsychopharmacology.*2005;30(Suppl. 1):S137.
- Post hoc analysis (with a subsample of subjects) in: Matza LS, Johnston JA, Faries DE, Malley KG, Brod M. Responsiveness of the Adult Attention-Deficit/Hyperactivity Disorder Quality of Life Scale (AAQoL). *Qual Life* *Res.* 2007;16(9):1511-1520.
- Adler LA, Spencer TJ, Levine LR, et al. Functional outcomes in the treatment of adults with ADHD. *J Atten Disord.* 2008;11(6):720-727.
- Pooled in: Adler L, Wilens T, Zhang S, et al. Retrospective safety analysis of atomoxetine in adult ADHD patients with or without comorbid alcohol abuse and dependence. *Am J Addict.* 2009;18(5):393-401.
- Previous reference related to Adler L, Wilens T, Zhang S, et al. Safety of atomoxetine in ADHD patients with or without comorbid alcohol abuse and dependence. Proceedings of the 70th Annual Scientific Meeting of the College on Problems of Drug Dependence; 2008 June 16-21; San Juan, Puerto Rico, USA2008:2.
- https://clinicaltrials.gov/ct2/show/NCT00190931
- https://assets.contentful.com/hadumfdtzsru/6JMvAj7xM4Q6McgwKiYSwg/062c958c27aa48897f2b098839213bd3/Atomoxetine-B4Z-MC-LYBV.pdf
- https://www.accessdata.fda.gov/drugsatfda_docs/nda/2002/21-411_Strattera.cfm
- Full CSR provided by manufacturer

*Reason for exclusion: Adult participants*

**3. Adler2008b, NRP104.303, NCT00334880**

- Adler LA, Goodman DW, Kollins SH, et al. Double-blind, placebo-controlled study of the efficacy and safety of lisdexamfetamine dimesylate in adults with attention-deficit/hyperactivity disorder. *J Clin Psychiatry.* 2008;69(9):1364-1373.
- Goodman D, Adler L, Kollins SH, et al. Efficacy and safety of lisdexamfetamine dimesylate in adults with attentiondeficit/ hyperactivity disorder. *Int J Neuropsychopharmacol.* 2008;11(Suppl. 1):292-293.
- Post hoc analysis with additional data pertinent for the present meta-analysis: Adler LA, Weisler RH, Goodman DW, Hamdani M, Niebler GE. Short-term effects of lisdexamfetamine demesylate on cardiovascular parameters in 4-week clinical trial in adults with attention-deficits/hyperactivity disorder. *J Clin Psychiatry.* 2009;70(12):1652-1661.
- Open label follow-up in: Weisler R, Young J, Mattingly G, Gao J, Squires L, Adler L. Long-term safety and effectiveness of lisdexamfetamine dimesylate in adults with attention-deficit/ hyperactivity disorder. *CNS Spectr.* 2009;14(10):573-585.
- Sleep outcomes in: Adler LA, Goodman D, Weisler R, Hamdani M, Roth T. Effect of lisdexamfetamine dimesylate on sleep in adults with attention-deficit/hyperactivity disorder. *Behav Brain Funct.* 2009;5:34.
- Pooled in: Goodman D, Faraone SV, Adler LA, Dirks B, Hamdani M, Weisler R. Interpreting ADHD rating scale scores: Linking ADHD rating scale scores and CGI levels in two randomized controlled trials of lisdexamfetamine dimesylate in ADHD. *Prim Psychiatry.* 2010;17(3):44-52.
- Pooled in: Lasser R, Dirks B, Adeyi B, Babcock T. Comparative efficacy and safety of lisdexamfetamine dimesylate and mixed amphetamine salts extended release in adults with attention-deficit/hyperactivity disorder. *Prim* *Psychiatry.* 2010;17(9):44-54.
- Pooled in: Surman CB, Roth T. Impact of stimulant pharmacotherapy on sleep quality: post hoc analyses of 2 large, double-blind, randomized, placebo-controlled trials. *J Clin Psychiatry.* 2011;72(7):903-908.
- Pooled in: Ginsberg L, Katic A, Adeyi B, et al. Long-term treatment outcomes with lisdexamfetamine dimesylate for adults with attention-deficit/hyperactivity disorder stratified by baseline severity. *Curr Med Res Opin.* 2011;27(6):1097-1107.
- Post hoc analyses in: Kollins SH, Youcha S, Lasser R, Thase ME. Lisdexamfetamine dimesylate for the treatment of attention deficit hyperactivity disorder in adults with a history of depression or history of substance use disorder. *Innov Clin Neurosci.* 2011;8(2):28-32.
- Pooled in: Waxmonsky JG, Waschbusch DA, Glatt SJ, Faraone SV. Prediction of placebo response in 2 clinical trials of lisdexamfetamine dimeslylate for the treatment of ADHD. *J Clin Psychiatry.* 2011;72(10):1366-1375.
- Analysis in subpopulation in: Babcock T, Dirks B, Adeyi B, Scheckner B. Efficacy of lisdexamfetamine dimesylate in adults with attention-deficit/hyperactivity disorder previously treated with amphetamines: analyses from a randomized, double-blind, multicenter, placebo-controlled titration study. *BMC Pharmacol Toxicol.* 2012;13:18.
- Post hoc analysis in: Faraone SV, Spencer TJ, Kollins SH, Glatt SJ, Goodman D. Dose response effects of lisdexamfetamine dimesylate treatment in adults with ADHD: an exploratory study. *J Atten Disord.* 2012;16(2):118-127.
- Pooled in: Mattingly GW, Weisler RH, Young J, et al. Clinical response and symptomatic remission in short- and long-term trials of lisdexamfetamine dimesylate in adults with attention-deficit/hyperactivity disorder. *BMC* *Psychiatry.* 2013;13:39.
- Included in: Coghill D, Sorooshian S, Caballero B. Safety outcomes from the clinical development programme for lisdexamfetamine dimesylate: A prodrug stimulant for the treatment of attention-deficit/hyperactivity disorder. *Eur* *Child Adolesc Psychiatry.* 2013;1):S224
- Pooled in: Adler LA, Dirks B, Adeyi B, Squires LA. Efficacy Outcomes in Age and Sex Subgroups from Two Clinical Trials of Lisdexamfetamine Dimesylate in the Treatment of Adults with Attention-Deficit/Hyperactivity Disorder. *Eur Psychiatry.* 2014;29, S1:1
- Pooled in: Weisler RH, Adler LA, Kollins SH, et al. Analysis of individual items on the attention deficit/hyperactivity disorder symptom rating scale in children and adults: The effects of age and sex in pivotal trials of lisdexamfetamine dimesylate. *Neuropsychiatr Dis Treat.* 2014;10:1-12.
- https://clinicaltrials.gov/ct2/show/NCT00334880
- <https://www.shireaustralia.com.au/-/media/shire/shireglobal/shireaustralia/pdffiles/product%20information/vyvansepi>. pdf
- Additional data/information provided by Shire from full CSR

*Reason for exclusion: Adult participants*

**4. Adler2009a, B4Z-US-LYDQ, NCT00190879**

- Adler LA, Liebowitz M, Kronenberger W, et al. Atomoxetine treatment in adults with attention-deficit/hyperactivity disorder and comorbid social anxiety disorder. *Depress Anxiety.* 2009;26(3):212-221.
- https://clinicaltrials.gov/ct2/show/NCT00190879
- https://assets.contentful.com/hadumfdtzsru/5zxD0FZNfiQ6SSqmoqwwge/8b34012d75dbb9544834e1b4b57a03ab/Atomoxetine-B4Z-US-LYDQ.pdf
- https://www.accessdata.fda.gov/drugsatfda_docs/nda/2002/21-411_Strattera.cfm
- Additional data/information provided by Lilly

*Reason for exclusion: Adult participants*

**5. Alder2009b, B4Z-US-LYCU, NCT00190736**

- Adler LA, Spencer T, Brown TE, et al. Once-daily atomoxetine for adult attention-deficit/hyperactivity disorder: a 6-month, double-blind trial. *J Clin Psychopharmacol.* 2009;29(1):44-50.
- Spencer TJ, Adler LA, Meihua Q, et al. Validation of the adult ADHD investigator symptom rating scale (AISRS). *J Atten Disord.* 2010;14(1):57-68.
- Summary and critical analysis in: Lall AS, Averbuch R. Once-daily atomoxetine in the treatment of attentiondeficit/hyperactivity disorder in adults. *Curr Psychiatry Rep.* 2010;12(5):363-365.
- Additional analysis in: Brown TE, Holdnack J, Saylor K, et al. Effect of atomoxetine on executive function impairments in adults with ADHD. *J Atten Disord.* 2011;15(2):130-138.
- Pooled in: Wietecha LA, Clemow DB, Buchanan AS, Young JL, Sarkis EH, Findling RL. Atomoxetine Increased Effect over Time in Adults with Attention-Deficit/Hyperactivity Disorder Treated for up to 6 Months: Pooled Analysis of Two Double-Blind, Placebo-Controlled, Randomized Trials. *CNS Neurosci Ther.* 2016;22(7):546-557.
- https://clinicaltrials.gov/ct2/show/NCT00190736
- https://assets.contentful.com/hadumfdtzsru/4NjwYiwKeckyyuA0IIcSK8/45a6c712ee38a7f228914fa31b6cf6df/Atomoxetine-B4Z-US-LYCU.pdf
- https://www.accessdata.fda.gov/drugsatfda_docs/nda/2002/21-411_Strattera.cfm
- Full CSR provided by Lilly

*Reason for exclusion: Adult participants*

**6. Adler2009c, CR011560, NCT00326391**

- Adler LA, Zimmerman B, Starr HL, et al. Efficacy and safety of OROS methylphenidate in adults with attentiondeficit/hyperactivity disorder: a randomized, placebo-controlled, double-blind, parallel group, dose-escalation study. *J Clin Psychopharmacol.* 2009;29(3):239-247.
- Part of subjects participated in: Adler LA, Orman C, Starr HL, et al. Long-term safety of OROS methylphenidate in adults with attention-deficit/hyperactivity disorder: an open-label, dose-titration, 1-year study. *J Clin Psychopharmacol.* 2011;31(1):108-114. (NCT00326300).
- https://clinicaltrials.gov/ct2/show/NCT00326391
- https://www.accessdata.fda.gov/drugsatfda_docs/nda/2000/21-121_Concerta.cfm
- Additional data/information provided by Janssen

*Reason for exclusion: Adult participants*

**7. Adler2013, SPD489-403, NCT01101022**

- Adler LA, Dirks B, Deas PF, et al. Lisdexamfetamine dimesylate in adults with attention-deficit/ hyperactivity disorder who report clinically significant impairment in executive function: results from a randomized, double-blind, placebo-controlled study. *J Clin Psychiatry.* 2013;74(7):694-702.
- Adler LA, Dirks B, Deas P, et al. Self-Reported quality of life in adults with attention-deficit/hyperactivity disorder and executive function impairment treated with lisdexamfetamine dimesylate: a randomized, double-blind, multicenter, placebo-controlled, parallel-group study. *BMC Psychiatry.* 2013;13:253
- Weisler R, Ginsberg L, Dirks B, Deas P, Adeyi B, Adler LA. Treatment With Lisdexamfetamine Dimesylate Improves Self- and Informant-Rated Executive Function Behaviors and Clinician- and Informant-Rated ADHD Symptoms in Adults: Data From a Randomized, Double-Blind, Placebo-Controlled Study. *J Atten Disord.* 2014, in press, 10.1177/1087054713518242
- Post hoc analyses: Adler L, Wu J, Madhoo M, Caballero B. Post hoc analyses of the efficacy of lisdexamfetamine dimesylate in adults previously treated with attention deficit/hyperactivity disorder medication. *Eur Psychiatry.* 2015;30, S1:566.
- https://clinicaltrials.gov/ct2/show/NCT01101022
- http://www.shiretrials.com/-/media/files/clinical%20trials/clinicaltrialsen/clinical%20study%20reports/shirespd489-403-clinical-study-report-redact.pdf
- Additional data/information provided by Shire

*Reason for exclusion: Adult participants*

**8. Amiri2008**

- Amiri S, Mohammadi MR, Mohammadi M, Nouroozinejad GH, Kahbazi M, Akhondzadeh S. Modafinil as a treatment for Attention-Deficit/Hyperactivity Disorder in children and adolescents: a double blind, randomized clinical trial. *Prog Neuropsychopharmacol Biol Psychiatry.* 2008;32(1):145-149.

*Reason for exclusion: Head to head study*

**9. Arnold2006**

- Arnold LE, Aman MG, Cook AM, et al. Atomoxetine for hyperactivity in autism spectrum disorders: placebocontrolled crossover pilot trial. *J Am Acad Child Adolesc Psychiatry.* 2006;45(10):1196-1205.

*Reason for exclusion: Concomitant psychotropic medications allowed*

**10. Arnold2014, C1538/2027/AD/US, NCT00315276**

- Arnold VK, Feifel D, Earl CQ, Yang R, Adler LA. A 9-week, randomized, double-blind, placebo-controlled, parallel-group, dose-finding study to evaluate the efficacy and safety of modafinil as treatment for adults with ADHD. *J Atten Disord.* 2014;18(2):133-144.
- https://clinicaltrials.gov/ct2/show/NCT00315276 (additional ID: C1538/2027/AD/US)

*Reason for exclusion: Adult participants*

**11. Bain2013, NCT00429091**

- Bain E, Robieson W, Garimella T, Abi-Saab W, Apostol G, Saltarelli MD. A randomized, double-blind, placebocontrolled Phase 2 study of alpha4beta2 agonist ABT-894 in adults with ADHD. *Biochem Pharmacol.* 2011;82(8):1043.
- Bain EE, Robieson W, Pritchett Y, et al. A randomized, double-blind, placebo-controlled phase 2 study of 42 agonist ABT-894 in adults with ADHD. *Neuropsychopharmacology*. 2013;38(3):405-413
- <https://clinicaltrials.gov/ct2/show/NCT00429091>

*Reason for exclusion: Adult participants*

**12. Bedard2015, NCT00183391**

- Bedard AC, Stein MA, Halperin JM, Krone B, Rajwan E, Newcorn JH. Differential impact of methylphenidate and atomoxetine on sustained attention in youth with attention-deficit/hyperactivity disorder. *J Child Psychol* *Psychiatry.* 2015;56(1):40-48.
- Newcorn J. ADHD and disruptive behavior disorders: Neurobiology and response to stimulant and non-stimulant treatment. *Neuropsychopharmacology.* 2016;41:S56.
- https://www.clinicaltrials.gov/ct2/show/NCT00183391
- http://www.shirecanada.com/-/media/shire/shireglobal/shirecanada/pdffiles/product%20information/adderall-xr-pmen.pdf
- <https://www.accessdata.fda.gov/drugsatfda_docs/nda/2001/21303_Adderall.cfm>

*Reason for exclusion: Head to head study*

**13. Biederman2006a, (subsample of NCT00181571)**

- Biederman J, Spencer T, Surman C, et al. A double-blind placebo-controlled randomized study of OROS methylphenidate in the treatment of adults with attention deficit hyperactivity disorder (ADHD): An interim analysis. *Biol Psychiatry.* 2005;57(8):106S-106S
- Biederman J, Mick E, Surman C, et al. A randomized, double-blind, placebo-controlled study of OROS methylphenidate in the treatment of adults with attention-deficit/hyperactivity disorder. *Eur* *Neuropsychopharmacol.*2005;15(Suppl. 3):S631
- Biederman J, Mick E, Surman C, et al. A randomized, placebo-controlled trial of OROS methylphenidate in adults with attention-deficit/hyperactivity disorder. *Biol Psychiatry.* 2006;59(9):829-835.
- Post hoc analysis in: Mick E, Biederman J, Spencer T, Faraone SV, Sklar P. Absence of association with DAT1 polymorphism and response to methylphenidate in a sample of adults with ADHD. *Am J Med Genet B* *Neuropsychiatr Genet.* 2006;141B(8):890-894
- Pooled in: Biederman J, Mick EO, Surman C, et al. Comparative acute efficacy and tolerability of OROS and immediate release formulations of methylphenidate in the treatment of adults with attention-deficit/hyperactivity disorder. *BMC Psychiatry.* 2007;7:49.
- Post hoc analysis in: Mick E, Faraone SV, Spencer T, Zhang HF, Biederman J. Assessing the validity of the Quality of Life Enjoyment and Satisfaction Questionnaire Short Form in adults with ADHD. *J Atten Disord*. 2008;11(4):504-509
- Related to previous: Biederman J, Spencer TJ, Mick E, Suppl. Comparative acute efficacy and tolerability of OROS and immediate release formulations of methylphenidate in the treatment of adults with ADHD. Eur Neuropsychopharmacol. 2006; 16: 8-21
- Related to previous: Biederman J. Comparative and acute efficacy and tolerability of OROS and immediate release formulations of methylphenidate in the treatment of adults with attention deficit hyperactivity disorder. *Biol* *Psychiatry*. 2006; 59(8, Suppl. S): 115.
- Part of subjects from this study participated in: Bush G, Spencer TJ, Holmes J, et al. Functional magnetic resonance imaging of methylphenidate and placebo in attention-deficit/hyperactivity disorder during the multi-source interference task. *Arch Gen Psychiatry.* 2008;65(1):102-114

Note: Biederman2006a is a subsample of Biederman2010 (NCT00181571) (see list of excluded studies)

*Reason for exclusion: Adult participants*

**14. Biederman2012, 2008P000971, NCT00801229**

- Biederman J, Fried R, Hammerness P, et al. The effects of lisdexamfetamine dimesylate on the driving performance of young adults with ADHD: a randomized, double-blind, placebo-controlled study using a validated driving simulator paradigm. *J Psychiatr Res.* 2012;46(4):484-491.
- Biederman J, Fried R, Hammerness P, et al. The effects of lisdexamfetamine dimesylate on driving behaviors in young adults with ADHD assessed with the Manchester driving behavior questionnaire. *J Adolesc Health.* 2012;51(6):601-607.
- Biederman J, Fried R. The effects of lisdexamfetamine dimesylate on the driving performance of young adults with ADHD. *Eur Neuropsychopharmacol.* 2012;22:S436.
- https://clinicaltrials.gov/ct2/show/NCT00801229 (other ID: 2008P000971)

*Reason for exclusion: Adult participants*

**15. Biehl2016**

- Biehl SC, Merz CJ, Dresler T, et al. Increase or Decrease of fMRI Activity in Adult Attention Deficit/ Hyperactivity Disorder: Does It Depend on Task Difficulty? *Int J Neuropsychopharmacol.* 2016; doi: 10.1093/ijnp/pyw049
- https://clinicaltrials.gov/ct2/show/NCT01351272
- https://www.clinicaltrialsregister.eu/ctr-search/search?query=eudract_number:2008-006242-26 (EU 2008-006242-26)
- Additional information provided by the first author

*Reason for exclusion: Adult participants*

**16. Bron2014**

- Bron TI, Bijlenga D, Marije Boonstra A, et al. OROS-methylphenidate efficacy on specific executive functioning deficits in adults with ADHD: A randomized, placebo-controlled cross-over study. *Eur Neuropsychopharmacol.* 2014;24(4):519-28
- http://isrctn.org/ISRCTN52392534 (ID: ISRCTN52392534)
- First author provided additional information

*Reason for exclusion: Adult participants*

**17. Buitelaar1996**

- Additional analyses in: Buitelaar JK, Van der Gaag RJ, Swaab-Barneveld H, Kuiper M. Prediction of clinical response to methylphenidate in children with attention-deficit hyperactivity disorder. *J Am Acad Child Adolesc* *Psychiatry.* 1995;34(8):1025-1032.
- Buitelaar JK, van der Gaag RJ, Swaab-Barneveld H, Kuiper M. Pindolol and methylphenidate in children with attention-deficit hyperactivity disorder. Clinical efficacy and side-effects. *J Child Psychol Psychiatry.* 1996;37(5):587-595.
- Buitelaar JK, Swaab Barneveld H, Gaag RJ. Prediction of Clinical Response to Methylphenidate in Children with ADHD. *Xth World Congress of Psychiatry.1996*

*Reason for exclusion: No number of participants with headaches (n) provided, only frequency of headaches in the article*

**18. Casas2013, EudraCT #: 2007-002111-82**

- Ramos-Quiroga JA, Kooij S, Trott GE, et al. Predictors of treatment outcome with long-acting methylphenidate in attention deficit hyperactivity disorder in adults. *Eur Neuropsychopharmacol*. 2009;19:S352-S3.
- Pooled in: Buitelaar JK, Sobanski E, Stieglitz RD, Dejonckheere J, Waechter S, Schauble B. Predictors of placebo response in adults with attention-deficit/hyperactivity disorder: data from 2 randomized trials of osmotic-release oral system methylphenidate. *J Clin Psychiatry.* 2012;73(8):1097-1102.
- Casas M, Rosler M, Sandra Kooij JJ, et al. Efficacy and safety of prolonged-release OROS methylphenidate in adults with attention deficit/hyperactivity disorder: a 13-week, randomized, double-blind, placebo-controlled, fixeddose study. *World J Biol Psychiatry.* 2013;14(4):268-281.
- Post hoc analysis in: Kooij JJ, Rosler M, Philipsen A, et al. Predictors and impact of non-adherence in adults with attention-deficit/hyperactivity disorder receiving OROS methylphenidate: results from a randomized, placebocontrolled trial. *BMC Psychiatry.* 2013;13:36.
- https://clinicaltrials.gov/ct2/show/NCT00714688
- https://www.clinicaltrialsregister.eu/ctr-search/search?query=eudract_number:2007-002111-82
- <https://yoda.yale.edu/sites/default/files/nct00714688.pdf>
- NCT00714688. (2008). A Study to Evaluate Effectiveness and Safety of Prolonged Release OROS Methylphenidate in Adults With Attention Deficit Hyperactivity Disorder. Https://clinicaltrials.gov/show/nct00714688. Retrieved from http://cochranelibrary-wiley.com/o/cochrane/clcentral/articles/740/CN-01519740/frame.html
- Additional information from study authors

*Reason for exclusion: Adult participants*

**19. Casat1989**

- Casat CD, Pleasants DZ, Fleet JV. A double-blind trial of bupropion in children with attention-deficit disorder. *Psychopharmacol Bull.* 1987;23(1):120-122.
- Casat CD, Pleasants DZ, Schroeder DH, Parler DW. Bupropion in children with attention deficit disorder. *Psychopharmacol Bull.* 1989;25(2):198-201.

*Reason for exclusion: No usable safety data*

**20. Connor2000**

- Connor DF, Barkley RA, Davis HT. A pilot study of methylphenidate, clonidine, or the combination in ADHD comorbid with aggressive oppositional defiant or conduct disorder. *Clin Pediatr (Phila).* 2000;39(1):15-25.

*Reason for exclusion: Head to head study*

**21. Cook1993**

- Cook JR. The Effects of Methylphenidate on Resource Allocation in the Mental Processing of ADD Children [PhD thesis]. University of North Dakota, 1989.
- Cook JR, Mausbach T, Burd L, et al. A preliminary study of the relationship between central auditory processing disorder and attention deficit disorder. *J Psychiatry Neurosci.* 1993;18(3):130-137.

*Reason for exclusion: No usable safety data*

**22. Döpfner2003**

- Lehmkuhl G. Placebo-controlled, double-blind multicenter trial on the efficacy of sustained-release methylphenidate in children suffering from attention deficit hyperactivity disorder (ADHD), Phase III. Integrated Final Report. Institut für medizinische Informatik, *Biometrie und Epidemiologie, Universitätsklinikum Essen*, Trial no 6520-9979- 02, 1-9 2002
- Dopfner M, Banaschewski T, Schmidt J, et al. Langzeitwirksames Methylphenidat bei Kinderen mit Aufmerksamkeitsdefizit-Hyperaktivitatsstorungen-eine multizentrische Studie [Long-acting methylphenidate preparation in children with ADHD-a multicenter study]. Nervenheilkunde. 2003(2):85-92.
- Sinzig JK, Döpfner M, Plück J, Banaschewski T, Stephani U, Lehmkuhl G, et al. Does a morning dose of methylphenidate retard reduce hyperkinetic symptoms in the afternoon? [Lassen sich hyperkinetische auffälligkeiten am nachmittag durch eine morgengabe von methylphenidat retard vermindern?]. *Z Kinder Jugendpsychiatr* *Psychother*. 2004;32 (4):225–33.
- Sinzig J, Döpfner M, Lehmkuhl G, Uebel H, Schmeck K, Poustka F, et al. Long-acting methylphenidate has an effect on aggressive behavior in children with attention-deficit/ hyperactivity disorder. *J Child Adolesc* *Psychopharmacol.* 2007;17(4):421–32.

*Reason for exclusion: No usable safety data*

**23. Durell2013, B4Z-US-LYDZ, NCT00510276**

- This study was presented in poster form at the Society of Biological Psychiatry 65th Annual Convention & Scientific Program, May 2010
- Altin M, Alev L, Durell TM, et al. Atomoxetine for the treatment of ADHD in young adults with an assessment of associated functional outcomes. *Klinik Psikofarmakoloji Bulteni.* 2011;21:S135-S136.
- Durell TM, Adler LA, Williams DW, et al. Atomoxetine treatment of attention-deficit/hyperactivity disorder in young adults with assessment of functional outcomes: a randomized, double-blind, placebo-controlled clinical trial. *J Clin Psychopharmacol.* 2013;33(1):45-54.
- Adler LA, Clemow DB, Williams DW, Durell TM. Atomoxetine effects on executive function as measured by the BRIEF--a in young adults with ADHD: a randomized, double-blind, placebo-controlled study. *PLoS ONE* *[Electronic Resource].* 2014;9(8):e104175.
- Addendum neurophysiological study (not pertinent for the present meta-analysis): Leuchter AF, McGough JJ, Korb AS, et al. Neurophysiologic predictors of response to atomoxetine in young adults with attention deficit hyperactivity disorder: a pilot project. *J Psychiatr Res.* 2014;54:11-18.
- https://clinicaltrials.gov/ct2/show/NCT00510276 (additional ID: B4Z-US-LYDZ)
- Lilly provided full CSR

*Reason for exclusion: Adult participants*

**24. Efron1997**

- Efron D, Jarman F, Barker M. Methylphenidate versus dexamphetamine in children with attention deficit hyperactivity disorder: A double-blind, crossover trial. *Pediatrics.* 1997;100(6):E6.
- Efron D, Jarman F, Barker M. Side effects of methylphenidate and dexamphetamine in children with attention deficit hyperactivity disorder: a double-blind, crossover trial. *Pediatrics.* 1997;100(4):662-666.
- Efron D, Jarman FC, Barker MJ. Child and parent perceptions of stimulant medication treatment in attention deficit hyperactivity disorder. *J Paediatr Child Health.* 1998;34(3):288-292.
- Efron DJ. Methylphenidate vs dexamphetamine in children with attention deficit hyperactivity disorder: a doubleblind cross-over trial. *J Paediatr Child Health.* 1997:A21.
- Follow-up in: Efron D, Jarman FC, Barker MJ. Medium-term outcomes are comparable with short-term outcomes in children with attention deficit hyperactivity disorder treated with stimulant medication. *J Paediatr Child Health.* 2000;36(5):457-461.

*Reason for exclusion: Head to head study*

**25. Findling2008 , NCT00444574**

- Swanson JM. Transdermal methylphenidate more effective than placebo for treating ADHD. *Evidence-based mental health.* 2008;11(4):118.
- Findling RL et al. A randomized, double-blind, placebo-controlled, parallel-group study of methylphenidate transdermal system in pediatric patients with attention-deficit/hyperactivity disorder. *J Clin Psychiatry*. 2008;69(1):149-59.
- Erratum in: Findling RL, Bukstein OG, Melmed RD, Lopez FA, Sallee FR, Arnold LE, et al. “A randomized, double-blind, placebo-controlled, parallel-group study of methylphenidate transdermal system in pediatric patients with attention deficit/hyperactivity disorder”: correction. *J Clin Psychiatry.* 2008;69(2):329.
- Faraone SV, Glatt SJ, Bukstein OG, Lopez FA, Arnold LE, Findling RL. Effects of once-daily oral and transdermal methylphenidate on sleep behavior of children with ADHD. *J Atten Disord.* 2009;12(4):308-315.
- Extension study: Findling RL, Katic A, Rubin R, Moon E, Civil R, Li Y. A 6-month, open-label, extension study of the tolerability and effectiveness of the methylphenidate transdermal system in adolescents diagnosed with attention-deficit/hyperactivity disorder. *J Child Adolesc Psychopharmacol.* 2010;20(5):365-375. (NCT00501293)
- <https://clinicaltrials.gov/ct2/show/NCT00444574>

*Reason for exclusion: No number of participants with headaches (n) provided*

**26. Frick2017, SPD465-303, NCT00152022**

- Spencer TJ, Adler LA, Weisler RH, Youcha SH, Anderson CS, Silverberg A. Efficacy and safety of a novel enhanced extended-release amphetamine formulation (triple-bead mixed amphetamine salts, SPD465) in adults with ADHD. Presented at: 160th Annual Meeting of the American Psychiatric Association; May 19–24, 2007; San Diego, CA.
- Pooled in: Brown TE, Landgraf JM. Improvements in executive function correlate with enhanced performance and functioning and health-related quality of life: evidence from 2 large, double-blind, randomized, placebo-controlled trials in ADHD. *Postgrad Med.* 2010;122(5):42-51.
- Frick G, Yan B, Adler LA. Triple-bead mixed amphetamine salts (SHP465) in adults with ADHD: results of a phase 3, doubel-blind, randomized, forced-dose trial. *J Att Dis*. 2017 Apr 1:1087054717696771. doi: 10.1177/108705471769677
- Spencer TJ, Anderson CS, Silverberg A, Youcha SH. Triple-bead mixed amphetamine salts (SPD465) improves hyperactivity/impulsivity and inattentiveness in adults with ADHD. *Biol Psychiatry*. 2007;61:171S-2S.
- Spencer TJ, Anderson CS, Silverberg A, Youcha S. Improvement in hyperactivity/impulsivity and inattentiveness associated with adult ADHD after triple-bead mixed amphetamine salts (SPD465) treatment. *Biol Psychiatry.* 2007;61(8, Suppl. S):172S.
- https://clinicaltrials.gov/ct2/show/NCT00152022 (additional ID: SPD463-303)

*Reason for exclusion: Adult participants*

**27. Ginsberg2012, EUCTR2006-002553-80-SE**

- Ginsberg Y, Lindefors N. Methylphenidate treatment of adult male prison inmates with attention-deficit hyperactivity disorder: randomised double-blind placebo-controlled trial with open-label extension. *Br J Psychiatry.* 2012;200(1):68-73.
- Long term non controlled phase in: Ginsberg Y, Hirvikoski T, Grann M, Lindefors N. Long-term functional outcome in adult prison inmates with ADHD receiving OROS-methylphenidate. *Eur Arch Psychiatry Clin Neurosci*. Dec 2012;262(8):705-724.
- Grann M, Ginsberg Y, Hirvikoski T, Lindefors N. Methylphenidate treatment of adult prison inmates with ADHD: A randomised double-blind placebo-controlled trial with open-label extension. *Acta Neuropsychiatr*. 2013;1):13.
- Long term non controlled phase in: Ginsberg Y, Hirvikoski T, Grann M, Lindefors N. Osmotic-release oral system methylphenidate (OROS-MPH) treatment of adult prison inmates with ADHD: A randomised controlled trial with open-label extension. *Eur Psychiatry.* 2013;28.
- Ginsberg Y. Pharmacological treatment of offenders with ADHD. *ADHD Attention Deficit and Hyperactivity Disorders.* 2015;7:S4.
- Follow-up in: Ginsberg Y, Langstrom N, Larsson H, Lindefors N. Long-term treatment outcome in adult male prisoners with attention- deficit/hyperactivity disorder: Three-year naturalistic follow-up of a 52-week methylphenidate trial. *J Clin Psychopharmacol.* 2015;35(5):535-543.
- https://clinicaltrials.gov/ct2/show/NCT00482313
- https://www.clinicaltrialsregister.eu/ctr-search/search?query=eudract_number:2006-002553-80
- Additional information from study author

*Reason for exclusion: Adult participants*

**28. Goodman2016, NCT00937040**

- Goodman DW, Starr HL, Ma YW, Rostain AL, Ascher S, Armstrong RB. Randomized, 6-Week, Placebo-Controlled Study of Treatment for Adult Attention-Deficit/Hyperactivity Disorder: Individualized Dosing of Osmotic-Release Oral System (OROS) Methylphenidate With a Goal of Symptom Remission. *J Clin* *Psychiatry.* 2017;78(1):105-114
- https://clinicaltrials.gov/ct2/show/NCT00937040
- Additional information form Janssen

*Reason for exclusion: Adult participants*

**29. Goto2017, B4Z-JE-LYEE, NCT00962104**

- Hirata Y, Goto T, Takita Y, et al. Efficacy and safety of atomoxetine in Asian adults with ADHD: A multinational 10-week randomized, double-blind placebo-controlled Asian study. *Int J Neuropsychopharmacol.* 2012;15:221-222.
- Goto T, Hirata Y, Takita Y, et al. Efficacy and Safety of Atomoxetine Hydrochloride in Asian Adults With ADHD. *J Atten Disord. 2017;212): 100-109.*(Former reference: Goto T, Hirata Y, Takita Y, et al. Efficacy and Safety of Atomoxetine Hydrochloride in Asian Adults With ADHD: A Multinational 10-Week Randomized Double-Blind Placebo-Controlled Asian Study. *J Atten Disord.* 2013.)
- Post hoc analysis in Korean sample in: Lee SI, Song DH, Shin DW, et al. Efficacy and safety of atomoxetine hydrochloride in Korean adults with attention-deficit hyperactivity disorder. *Asia Pac Psychiat.* 2014;6(4): 386-396*.*
- Follow-up, open label study in: Hirata Y, Goto T, Takita Y, et al. Long-term safety and tolerability of atomoxetine in Japanese adults with attention deficit hyperactivity disorder. *Asia Pac Psychiatry*. 2014;6(3):292-301
- Conference proceeding related to previous reference: Hirata Y, Goto T, Takita Y, et al. Long-term safety and efficacy of atomoxetine in adult ADHD Japanese patients. *Eur Psychiatry*. 2013;28.
- https://clinicaltrials.gov/ct2/show/NCT00962104 (other ID:B4Z-JE-LYEE**)**
- Full CSR provided by Lilly

*Reason for exclusion: Adult participants*

**30. Grizenko2012**

- Ben AL, Grizenko N, Mbekou V, Lageix P, Baron C, Schwartz G, et al. Dopamine transporter gene and behavioral response to methylphenidate (MPH) in children with attention deficit hyperactivity disorder (ADHD). *Am J Med* *Genet A*. Abstracts of the Xth World Congress of Psychiatric Genetics; 2002; Brussels, Belgium. 2002; Vol. 114(7):724–5.
- Grizenko N, Lachance M, Collard V, et al. Sensitivity of tests to assess improvement in ADHD symptomatology. *Can J Psychiatry.* 2004;13(2):36-39.
- Schwartz G, Amor LB, Grizenko N, Lageix P, Baron C, Boivin DB, et al. Actigraphic monitoring during sleep of children with ADHD on methylphenidate and placebo. *J Am Acad Child Adolesc Psychiatry.* 2004;43(10):1276–82.
- Gruber R, Grizenko N, Schwartz G, Benamor L, Terstepanian M, Joober R. The associations between sleep and attention in children with attention deficit hyperactivity disorder while on placebo and while on methylpheniclate. *Sleep (Rochester).* 2005;28(Suppl. S):A93-A94.
- Overlap with subjects from: Grizenko N, Bhat M, Schwartz G, Ter-Stepanian M, Joober R. Efficacy of methylphenidate in children with attention-deficit hyperactivity disorder and learning disabilities: a randomized crossover trial. *J Psychiatry Neurosci.* 2006;31(1):46-51.
- Grizenko N, Kovacina B, Amor LB, Schwartz G, Ter-Stepanian M, Joober R. Relationship between response to methylphenidate treatment in children with ADHD and psychopathology in their families. *J Am Acad Child Adolesc* *Psychiatry.* 2006;45(1):47-53.
- Gruber R, Grizenko N, Schwartz G, Ben Amor L, Gauthier J, De Guzman R, et al. Sleep and COMT polymorphismin ADHD children: preliminary actigraphic data. *J Am Acad Child Adolesc Psychiatry.* 2006;45(8):982–9.
- Gruber R, Grizenko N, Schwartz G, Bellingham J, Guzman R, Joober R. Performance on the continuous performance test in children with ADHD is associated with sleep efficiency. *Sleep.* 2007;30(8):1003-1009.
- Joober R, Grizenko N, Sengupta S, et al. Dopamine transporter 3'-UTR VNTR genotype and ADHD: a pharmacobehavioural genetic study with methylphenidate. Neuropsychopharmacology*.* 2007;32(6):1370-1376.
- Harvey WJ, Reid G, Grizenko N, Mbekou V, Ter-Stepanian M, Joober R. Fundamental movement skills and children with attention-deficit hyperactivity disorder: peer comparisons and stimulant effects. *J Abnorm Child* *Psychol.* 2007;35(5):871-882.
- Thesis related to the previous reference: Harvey WJ. Fundamental movement skills and associated physical activity experiences of children with ADHD. *Dissertation Abstracts International Section A: Humanities and Social* *Sciences.* 2007;68(3-A):927.
- Grizenko N, Shayan YR, Polotskaia A, Ter-Stepanian M, Joober R. Relation of maternal stress during pregnancy to symptom severity and response to treatment in children with ADHD. *J Psychiatry Neurosci.* 2008;33(1):10-16.
- Sengupta S, Grizenko N, Schmitz N, Schwartz G, Bellingham J, Polotskaia A, et al. COMT Val108/158Met polymorphism and the modulation of task-oriented behavior in children with ADHD. Neuropsychopharmacology*.* 2008;33(13):3069–77.
- Data from this cohort pooled in: Gruber R, Joober R, Grizenko N, Leventhal BL, Cook EH, Jr., Stein MA. Dopamine transporter genotype and stimulant side effect factors in youth diagnosed with attentiondeficit/ hyperactivity disorder. *J Child Adolesc Psychopharmacol.* 2009;19(3):233-239.
- Grizenko N, Paci M, Joober R. Is the inattentive subtype of ADHD different from the combined/hyperactive subtype? *J Atten Disord.* 2010;13(6):649-657.
- Ter-Stepanian M, Grizenko N, Zappitelli M, Joober R. Clinical response to methylphenidate in children diagnosed with attention-deficit hyperactivity disorder and comorbid psychiatric disorders. *Can J Psychiatry.* 2010;55(5):305-312.
- Thakur GA, Grizenko N, Sengupta SM, Schmitz N, Joober R. The 5-HTTLPR polymorphism of the serotonin transporter gene and short term behavioral response to methylphenidate in children with ADHD. *BMC Psychiatry.* 2010;10:50.
- Lee J, Grizenko N, Bhat V, Sengupta S, Polotskaia A, Joober R. Relation between therapeutic response and side effects induced by methylphenidate as observed by parents and teachers of children with ADHD. *BMC Psychiatry.* 2011;11:70.
- Grizenko N, Zhang DDQ, Polotskaia A, Joober R. Effcacy of methylphenidate in ADHD children across the normal and the gifted intellectual spectrum. *J Can Acad Child Adolesc Psychiatry.* 2012;21(4):282-288.
- Thakur GA, Sengupta SM, Grizenko N, Choudhry Z, Joober R. Comprehensive phenotype/genotype analyses of the norepinephrine transporter gene (SLC6A2) in ADHD: relation to maternal smoking during pregnancy. *PLoS One.* 2012;7(11):e49616.
- Labbe A, Liu A, Atherton J, et al. Refining psychiatric phenotypes for response to treatment: contribution of LPHN3 in ADHD. *Am J Med Genet B Neuropsychiatr Genet.* 2012;159B(7):776-785.
- Grizenko N, Cai E, Jolicoeur C, Ter-Stepanian M, Joober R. Effects of methylphenidate on acute math performance in children with attention-deficit hyperactivity disorder. *Can J Psychiatry.* 2013;58(11):632-639.
- Grizenko N, Rodrigues Pereira RM, Joober R. Sensitivity of scales to evaluate change in symptomatology with psychostimulants in different ADHD subtypes. *J Can Acad Child Adolesc Psychiatry.* 2013;22(2):153-158.
- Fortier ME, Sengupta SM, Grizenko N, Choudhry Z, Thakur G, Joober R. Genetic evidence for the association of the hypothalamic-pituitary-adrenal (HPA) axis with ADHD and methylphenidate treatment response. *Neuromolecular medicine.* 2013;15(1):122-132.
- <https://clinicaltrials.gov/ct2/show/NCT00483106>
- Fageera W, Grizenko N, Sengupta S, Joober R. The association between COMT (VAL158MET) and DRD3 (SER-9-GLY) genotypes and methylphenidate side effects. *Genetic epidemiology. Conference: 25th annual meeting of the international genetic epidemiology society, IGES 2016. Canada, 40*(7), 634. Retrieved from http://cochranelibrary-wiley.com/o/cochrane/clcentral/articles/343/CN-01423343/frame.html https://onlinelibrary.wiley.com/doi/pdf/10.1002/gepi.22001 doi:10.1002/gepi.22001
- Fageera W, Grizenko N, Sengupta SM., Joober R. Using behavioral dynamic approaches to test for gene-by-gene interaction in modulating adhd behaviors. *Genetic Epidemiology 2016; 40 (7)*: 634-635. doi:http://dx.doi.org/10.1002/gepi.22001
- Gruber R, Grizenko N, Schwartz G, Ben Amor L, Gauthier J, de Guzman R, Joober R. Sleep and COMT polymorphism in ADHD children: preliminary actigraphic data. J Am Acad Child Adolesc Psychiatry. 2006;45(8):982-989.
- Overlap with subjects in Bhat V, Sengupta SJ, Grizenko N et al. Therapeutic response in children with ADHD: role of observers and settings. World Journal of Pediatrics. 2020; 16:314–321.
- Bhat V, Sengupta SM, Grizenko N. Therapeutic Response to Methylphenidate in ADHD: Role of Child and Observer Gender. J Can Acad Child Adolesc Psychiatry. 2020; 29(1): 44–52.

*Reason for exclusion: Cross-over without wash out*

**31. Herring2012, NCT00475735**

- Herring WJ, Adler LA, Baranak CC, Liu K, Snavely D, Michelson D. Effects of the histamine inverse agonist MK- 0249 in adult attention deficit disorder: A randomized, controlled, crossover study. *Biol Psychiatry.* 2010;1):217S.
- Herring WJ, Wilens TE, Adler LA, et al. Randomized controlled study of the histamine H3 inverse agonist MK-0249 in adult attention-deficit/hyperactivity disorder. *J Clin Psychiatry.* 2012;73(7):e891-898.
- <https://clinicaltrials.gov/ct2/show/NCT00475735>

*Reason for exclusion: Adult participants*

**32. Huss2014 , CRIT124D2302, EUCTR2010-021533-31-DE, NCT01259492**

- Huss M, Ginsberg Y, Philipsen A, et al. 40-Week, randomized, double-blind, placebo-controlled, multicenter, efficacy and safety study of methylphenidate hydrochloride modified release (MPH-LA) in adults with attention deficit hyperactivity disorder (ADHD). *Eur Psychiatry.* 2013;28.
- Huss M, Ginsberg Y, Tvedten T, et al. Methylphenidate hydrochloride modified release improved inattention and hyperactivity/impulsivity scores in adult ADHD patients. *Eur Neuropsychopharmacol.* 2013;23:S604-S605.
- Open label extension (NCT01338818) in: Ginsberg Y, Arngrim T, Philipsen A, et al. Long-term (1 year) safety and efficacy of methylphenidate modified-release long-acting formulation (MPH-LA) in adults with attention-deficit hyperactivity disorder: a 26-week, flexible-dose, open-label extension to a 40-week, double-blind, randomised, placebo-controlled core study. *CNS Drugs.* 2014;28(10):951-962.
- Post hoc analysis in: Huss M, Ginsberg Y, Arngrim T, et al. Open-label dose optimization of methylphenidate modified release long acting (MPH-LA): a post hoc analysis of real-life titration from a 40-week randomized trial. *Clin Drug Investig.* 2014;34(9):639-649.
- Huss M, Ginsberg Y, Tvedten T, et al. Methylphenidate hydrochloride modified-release in adults with attention deficit hyperactivity disorder: a randomized double-blind placebo-controlled trial. *Adv Ther.* 2014;31(1):44-65.
- Additional analyses in: Huss M, Ginsberg Y, Arngrim T, et al. Added benefits of long-term treatment of methylphenidate modified-release long-acting formulation in adults with ADHD. *Eur Neuropsychopharmacol.* 2014;24:S210.
- Huss M, Ginsberg Y, Arngrim T, et al. Efficacy and safety of methylphenidate modified release long-acting after reinstatement of therapy in placebo treated patients. *Eur Neuropsychopharmacol.* 2014;24:S209-S210.
- Huss M, Ginsberg Y, Arngrim T, et al. Adult ADHD treated with methylphenidate modified-release (MPH-LA) maintained functional improvement over a period of 1 year. *ADHD Attention Deficit and Hyperactivity Disorders.* 2015;7:S50.
- Huss M, Ginsberg Y, Arngrim T, et al. Long-term safety of methylphenidate modified-release in adult ADHD upon continuous exposure up to 66 weeks. *ADHD Attention Deficit and Hyperactivity Disorders.* 2015;7:S50-S51.
- https://clinicaltrials.gov/ct2/show/NCT01259492
- https://www.clinicaltrialsregister.eu/ctr-search/search?query=eudract_number:2010-021533-31
- https://www.novctrd.com/CtrdWeb/displaypdf.nov?trialresultid=9544(study ID: CRIT124D2302)
- Additional information provided by Medice

*Reason for exclusion: Adult participants*

**33. Ivanov2020 (Conference proceedings. Includes change in ADHD ratings)**

- Ivanov I,Newcorn J, Krone B, et al. Neurobiological Basis of Reinforcement-Based Decision Making in Adults With ADHD Treated With Lisdexamfetamine Dimesylate. *Biological Psychiatry Supplement*. 2020: 87(9) S422-S423.

*Reason for exclusion: Adult participants*

**34. Iwanami2020**

- Iwanami, A., et al., Efficacy and Safety of Guanfacine Extended-Release in the Treatment of Attention-Deficit/Hyperactivity Disorder in Adults: Results of a Randomized, Double-Blind, Placebo-Controlled Study. *J Clin Psychiatry*, 2020. 81(3).

*Reasons for excludion: Adult participants*

**35. Jafarinia2012**

- Jafarinia M, Mohammadi MR, Modabbernia A, et al. Bupropion versus methylphenidate in the treatment of children with attention-deficit/hyperactivity disorder: randomized double-blind study. *Hum Psychopharmacol.* 2012;27(4):411-418.(ID: IRCT201012021556N18)

*Reason for exclusion: Head to head study*

**36. Jain2011, NCT00556959**

- Jain R, Segal S, Kollins SH, Khayrallah M. Clonidine extended-release tablets for pediatric patients with attentiondeficit/hyperactivity disorder. *J Am Acad Child Adolesc Psychiatry.* 2011;50(2):171-179.
- Summary in: Nguyen M, White KA, Bussing R. Clonidine extended release in the treatment of attention-deficit/hyperactivity disorder. *Curr Psychiatry Rep*. 2011;13(5):313-315.
- https://clinicaltrials.gov/ct2/show/NCT00556959

*Reason for exclusion: No number of participants with headaches (n) provided*

**37. Johnson2020**

- Johnson JK, Liranso T, Saylor K, et al. A Phase II Double-Blind, Placebo-Controlled, Efficacy and Safety Study of SPN-812 (Extended-Release Viloxazine) in Children With ADHD. J Atten Disord. 2020; 24(2): 348-358

*Reasons for excludion: Not the medication of our interest*

**38, 39. Kay2009a,b**

- Kay GG, Michaels MA, Pakull B. Simulated driving changes in young adults with ADHD receiving mixed amphetamine salts extended release and atomoxetine. *J Atten Disord.* 2009;12(4):316-329. (These data were presented in part at the 158th Annual Meeting of the American Psychiatric Association in Atlanta, GA, on May 24, 2005
- https://clinicaltrials.gov/ct2/show/NCT00557960 (additional ID: SLI381-316)

*Note: cross-over with no wash out; only usable data: drop outs in pre cross-over phase*

*Reason for exclusion: Adult participants*

**40. Kooij2004**

- Kooij JJ, Burger H, Boonstra AM, Van der Linden PD, Kalma LE, Buitelaar JK. Efficacy and safety of methylphenidate in 45 adults with attention-deficit/hyperactivity disorder. A randomized placebo-controlled doubleblind cross-over trial. *Psychol Med.* 2004;34(6):973-982.
- Boonstra AM, Kooij JJ, Oosterlaan J, Sergeant JA, Buitelaar JK. Does methylphenidate improve inhibition and other cognitive abilities in adults with childhood-onset ADHD? *J Clin Exp Neuropsychol.* 2005;27(3):278-298.
- Boonstra AM, Kooij JJ, Oosterlaan J, Sergeant JA, Buitelaar JK, Van Someren EJ. Hyperactive night and day? Actigraphy studies in adult ADHD: a baseline comparison and the effect of methylphenidate. *Sleep.* 2007;30(4):433-442.
- Kooij JS, Boonstra AM, Vermeulen SH, et al. Response to methylphenidate in adults with ADHD is associated with a polymorphism in SLC6A3 (DAT1). *Am J Med Genet B Neuropsychiatr Genet.* 2008;147B(2):201-208.
- Additional data provided by study author

*Reason for exclusion: Adult participants*

**41. Kurlan2002**

- Kurlan R, Tourette Syndrome Study Group. Treatment of attention-deficit-hyperactivity disorder in children with Tourette’s syndrome (TACT Trial). Ann Neurol.Abstracts from the American Neurological Association’s 125^th^ Annual Meeting; 2000 October 15-18; Boston, Massachusetts 2000;48(6):953.
- Kurlan R, Goetz CG, McDermott MP, et al. Treatment of ADHD in children with tics - A randomized controlled trial. *Neurology.* 2002;58(4):527-536.
- Kurlan R, Goldberg J. Clonidine and methylphenidate were effective for attention deficit hyperactivity disorder in children with comorbid tics. *Evidence-Based Medicine.* 2002;7(5):157.
- Deputy SR. Treatment of ADHD in children with tics: a randomized controlled trial. *Clin Pediatr (Phila).* 2002;41(9):736.
- Anon. (2002). Treatment of ADHD in children with tics. Hopital Extra, *Am J Nurs*. 102, 9, 24D.
- Goldberg J. Clonidine and methylphenidate were effective for attention deficit hyperactivity disorder in children with comorbid tics. *Evid Based Ment Health.* 2002, 5, 122
- Goldberg, J. (2002). Clonidine and methylphenidate were effective for attention deficit hyperactivity disorder in children with comorbid tics. *ACP J Club.* 137, 70.
- Additional data provided by study author

*Reason for exclusion: No usable safety data*

**42. Lin2014, NCT00922636**

- Lin DY, Kratochvil CJ, Xu W, et al. A randomized trial of edivoxetine in pediatric patients with attentiondeficit/hyperactivity disorder. *J Child Adolesc Psychopharmacol.* 2014;24(4):190-200.
- <https://clinicaltrials.gov/ct2/show/NCT00922636>

*Reason for exclusion: No pertinent ADHD medication in the present systematic review*

**43. Lin2016, NCT00917371**

- Lin HY, Gau SS. Atomoxetine Treatment Strengthens an Anti-Correlated Relationship between Functional Brain Networks in Medication-Naive Adults with Attention-Deficit Hyperactivity Disorder: A Randomized Double-Blind Placebo-Controlled Clinical Trial. *Int J Neuropsychopharmacol.* 2015;19(3).
- <https://clinicaltrials.gov/ct2/show/NCT00917371>
- Fan LY, Chou TL, Gau SS. Neural correlates of atomoxetine improving inhibitory control and visual processing in Drug-naïve adults with attention-deficit/hyperactivity disorder. Hum Brain Mapp. 2017;38(10):4850-4864.

*Reason for exclusion: Adult participants*

**44. McCracken2016**

- Nurmi EL, Mallya A, Mallya KS, et al. Pharmacogenetics of growth effects complicating ADHDtreatment. Neuropsychopharmacology*.* 2013;38:S492-S493.
- Nurmi EL, Mallya KS, McGough J, et al. Pharmacogenetic moderators of methylphenidate and guanfacine response in children and adolescents with adhd. Neuropsychopharmacology*.* 2012;38:S219.
- McCracken JT, McGough JJ, Loo SK, et al. Combined Stimulant and Guanfacine Administration in Attention-Deficit/Hyperactivity Disorder: A Controlled, Comparative Study. *J Am Acad Child Adolesc Psychiatry.* 2016;55(8):657-666.
- Sayer GR, McGough JJ, Levitt J, et al. Acute and Long-Term Cardiovascular Effects of Stimulant, Guanfacine, and Combination Therapy for Attention-Deficit/Hyperactivity Disorder. *Journal of child and adolescent* *psychopharmacology.* 2016;26(10):882-888.
- Bilder RM, Loo S, McGough JJ, et al. Cognitive effects of stimulant, guanfacine, and combined treatment in child and adolescent attention-deficit/hyperactivity disorder. *Biol Psychiatry.* 2016;1):158S.
- Loo SK, Bilder RM, Cho AL, et al. Effects of d-Methylphenidate, Guanfacine, and Their Combination on Electroencephalogram Resting State Spectral Power in Attention-Deficit/Hyperactivity Disorder. *J Am Acad Child* *Adolesc Psychiatry.* 2016;55(8):674-682.
- McCracken J. AACAP elaine schlosser lewis award for research on attention-deficit disorder can we improve treatments for attention-deficit/hyperactivity disorder? Journal of the american academy of child and adolescent psychiatry. Conference: 64th annual meeting american academy of child and adolescent psychiatry, AACAP 2017. United states, 56(10): S130. Retrieved from http://cochranelibrary-wiley.com/o/cochrane/clcentral/articles/314/CN-01452314/frame.html doi:10.1016/j.jaac.2017.09.005
- https://clinicaltrials.gov/ct2/show/NCT00429273

*Reason for exclusion: Head to head study*

**45. McRae-Clark2010, R21DA018221, NCT00360269**

- McRae-Clark AL, Carter RE, Killeen TK, Carpenter MJ, White KG, Brady KT. A placebo-controlled trial of atomoxetine in marijuana-dependent individuals with attention deficit hyperactivity disorder. *Am J Addict.* 2010;19(6):481-489. (ID: R21DA018221).
- https://clinicaltrials.gov/ct2/show/NCT00360269
- Additional data from study author

*Reason for exclusion: Adult participants*

**46. Medori2008 , LAMDA-I EUCTR2004-000730-37, NCT00246220**

- Medori R, Kooij S, Ramos-Quiroga JA, Buitelaar J, Lee E, Casas M. Efficacy and safety of OROS methylphenidate in adults with ADHD the long-acting methylphenidate in adult ADHD (LAMDA) trial. *J Neural Transm (Vienna).* 2007; 114(7).
- Medori R, Kooij S, Buitelaar J, et al. Double-blind study of the efficacy and safety of prolonged-release methylphenidate in adults with ADHD - the LAMDA trial. *Eur Neuropsychopharmacol.* 2007;17(Suppl. 4):S589.
- Sandner F. First results of the European LAMDA study on ADHD: Long-acting methylphenidate is effective and well tolerated in adults. [German]Retardiertes methylphenidat ist auch bei erwachsenen effektiv und vertraglich. *Journal fur Pharmakologie und Therapie.* 2007;16(6):181-182.
- Gerve M, Philipsen A, Roesler M, Sobanski E, Schauble B, Medori R, Trott GE. Effectiveness and compatibility of OROS (R)-methylphenidate for adult with ADHS - The long-acting methylphenidate in adult ADHD (LAMDA) trial*. Nervenarzt* 2007; 78(S2): 208-209; (46): 1-4.
- Roesler M, Trott GE, Philipsen A, Gerwe M, Lee E, Medori R, Schauble B, No. Efficacy and safety of OROS((R)) methylpheniclate in adults with ADHD: the long-acting methylphenidate in adult ADHD (LAMDA) trial. *Pharmacopsychiatry*. 2007; 40: 205.
- No authors listed. Erste Ergebnisse der europäischen LAMDA-Studie zu ADHS: Retardiertes Methylphenidat ist auch bei Erwachsenen effektiv und verträglich. *Journal für Pharmakologie und Therapie*. 2007; 16(6): 181.
- Kooij S, Medori R, Buitelaar J, Ramos-Quiroga JA, Lee E, Casas M. Open-label extension trial of the safety and tolerability of OROSO methylphenidate in adults with ADHD – the long-acting methylphenidate in adult ADHD (lamda) trial. *J Neural Transm (Vienna ).* 2007; 114(7).
- Kooij J, Medori R, Buitelaar J, Ramos-Quiroga J, Lee E, Casas M. Open-label extension trial of the safety and tolerability of OROS methylphenidate in adults with adhd - the long-acting methylphenidate in adult ADHD (LAMDA) trial. *Program and abstracts of the American Psychiatric Association 2007 Annual Meeting; May 19-24,* *2007; San Diego, California. Poster NR 647.*
- Medori R, Ramos-Quiroga JA, Casas M, et al. A randomized, placebo-controlled trial of three fixed dosages of prolonged-release OROS methylphenidate in adults with attention-deficit/hyperactivity disorder. *Biol Psychiatry.* 2008;63(10):981-989.
- Van Oene JC, Imhof L, Nyerod HJ, et al. Efficacy outcomes during longterm prolonged-release methylphenidate treatment of adults with ADHD. *Eur Neuropsychopharmacol.* 2009;19:S353-S4.
- Schaeuble B, Orman C, Palumbo JM. Efficacy of prolonged-release methylphenidate in a randomized controlled trial in adults with ADHD: Secondary endpoints. *Eur Neuropsychopharmacol.* 2009;19:S679-S680 (link confirmed by Dr Buitelaar)
- Schaeuble B, Hofecker M, Buitelaar JK, et al. Long-term treatment outcomes in adults with ADHD treated with OROS MPH. *Eur J Neurol.* 2010;17(Suppl. 3, Sp. Iss. SI):470.
- Post hoc analysis: Buitelaar JK, Kooij JJS, Ramos-Quiroga JA, et al. Predictors of treatment outcome in adults with ADHD treated with OROS methylphenidate. *Prog Neuropsychopharmacol Biol Psychiatry.* 2011(2):554-560.
- Open label extension in : Buitelaar JK, Casas M, Philipsen A, et al. Functional improvement and correlations with symptomatic improvement in adults with attention deficit hyperactivity disorder receiving long-acting methylphenidate. *Psychol Med.* 2012;42(1):195-204.
- Open label extension in : Buitelaar JK, Trott GE, Hofecker M, et al. Long-term efficacy and safety outcomes with OROS-MPH in adults with ADHD. *Int J Neuropsychopharmacol.* 2012(1):1-13. (NCT00307684)
- Pooled analysis in: Buitelaar JK, Sobanski E, Stieglitz RD, Dejonckheere J, Waechter S, Schauble B. Predictors of placebo response in adults with attention-deficit/hyperactivity disorder: data from 2 randomized trials of osmoticrelease oral system methylphenidate. *J Clin Psychiatry.* 2012;73(8):1097-1102.
- Rosler M, Ginsberg Y, Arngrim T, et al. Correlation of symptomatic improvements with functional improvements and patient-reported outcomes in adults with attention-deficit/hyperactivity disorder treated with OROS methylphenidate. *World J Biol Psychiatry.* 2013;14(4):282-290.
- https://clinicaltrials.gov/ct2/show/NCT00246220
- Additional data from Janssen

*Reason for exclusion: Adult participants*

**47, 48. Michelson2003a,b**

- Michelson D, Adler L, Spencer T, et al. Atomoxetine in adults with ADHD: two randomized, placebo-controlled studies. *Biol Psychiatry.* 2003;53(2):112-120.
- Spencer TJ. Efficacy and safety of atomoxetine in adults with ADHD. *156th Annual Meeting of the American Psychiatric Association, May 17-22, San Francisco CA.* 2003:No. 32.
- Biederman J, Faraone S, Spencer T, Michelson D, Jones D, Adler L. Effects of atomoxetine treatment on executive functioning in adults with attention-deficit/hyperactivity disorder (ADHD). *Eur Neuropsychopharmacol.* 2003;13(Supplement 4):S458.
- Michelson D, Marchant B. Emotional Dysregulation in ADHD and Response to Atomoxetine. *156th Annual Meeting of the American Psychiatric Association, May 17-22, San Francisco CA.* 2003:Nr640
- Included in: Wernicke JF, Adler L, Spencer T, et al. Changes in symptoms and adverse events after discontinuation of atomoxetine in children and adults with attention deficit/hyperactivity disorder: a prospective, placebo-controlled assessment. *J Clin Psychopharmacol.* 2004;24(1):30-35.
- Faraone SV, Biederman J, Spencer T, et al. Efficacy of atomoxetine in adult attention-deficit/hyperactivity disorder: a drug-placebo response curve analysis. *Behav Brain Funct.* 2005;1:16.
- Faraone SV, Biederman J, Spencer T, et al. Atomoxetine and stroop task performance in adult attentiondeficit/hyperactivity disorder. *J Child Adolesc Psychopharmacol.* 2005;15(4):664-670.
- Reimherr FW, Faraone SV, Marchant BK, et al. Gender differences in adults with ADHD, pretreatment and following treatment with atomoxetine under double-blind conditions. *Eur Neuropsychopharmacol.* 2005;15(Suppl.3):S604,S601.
- Reimherr FW, Marchant BK, Strong RE, et al. Emotional dysregulation in adult ADHD and response to atomoxetine. *Biol Psychiatry.* 2005;58(2):125-131.
- Previous reference related to: Reimherr FRE, Hedges DW, West SA, Adler LA, Michelson D, Marchant B. Emotional Dysregulation in ADHD and Response to Atomoxetine. *156th Annual Meeting of the American* *Psychiatric Association, May 17-22, San Francisco CA.* 2003:Nr640
- Post hoc analysis in: Spencer TJ, Faraone SV, Michelson D, et al. Atomoxetine and adult attentiondeficit/hyperactivity disorder: the effects of comorbidity. *J Clin Psychiatry.* 2006;67(3):415-420.
- Robison RJ, Reimherr FW, Marchant BK, Faraone SV, Adler LA, West SA. Gender differences in 2 clinical trials of adults with attention-deficit/hyperactivity disorder: A retrospective data analysis. *J Clin Psychiatry.* 2008;69(2):213-221.
- Pooled in: Adler LA, Faraone SV, Spencer TJ, et al. The reliability and validity of self- and investigator ratings of ADHD in adults. *J Atten Disord.* 2008;11(6):711-719.
- Pooled in: Reimherr FW, Olsen J, Marchant BK, et al. ADHD Symptoms Associated with Comorbid Alcohol Dependence Or Abuse: Comparison of Subject Attributes within ADHD Clinical Trials. *Biol Psychiatry*. 2009;65:116S.
- Pooled in: Adler L, Wilens T, Zhang S, et al. Retrospective safety analysis of atomoxetine in adult ADHD patients with or without comorbid alcohol abuse and dependence. *Am J Addict*. 2009;18(5):393-401.
- Post hoc analysis in: Durell T, Adler L, Wilens T, Paczkowski M, Schuh K. Atomoxetine treatment for ADHD: younger adults compared with older adults. *J Atten Disord.* 2010;13(4):401-406.
- Follow-up in: Marchant BK, Reimherr FW, Halls C, et al. Long-term open-label response to atomoxetine in adult ADHD: influence of sex, emotional dysregulation, and double-blind response to atomoxetine. *Atten Defic Hyperact* *Disord.* 2011;3(3):237-244.
- Pooled in: Adler LA, Wilens T, Zhang S, et al. Atomoxetine treatment outcomes in adolescents and young adults with attention-deficit/hyperactivity disorder: results from a post hoc, pooled analysis. *Clin Ther.* 2012;34(2):363-373.
- Dittmann RW, Adler L, Michelson D, Wernicke J. Efficacy and safety of atomoxetine in adults with attention deficit/hyperactivity disorder. *Pharmacopsychiatry.* 2003;36:221.
- https://assets.contentful.com/hadumfdtzsru/5xQa80jOzm4qQE4GUYO8YQ/68a9810927a09ee745f4819bd9098dde/Atomoxetine-B4Z-MC-LYAA.pdf
- https://assets.contentful.com/hadumfdtzsru/6fLop0oa0oYGcu8M0mWWwA/17754d66b6dbcecf14f2e93aa21afb6b/Atomoxetine-B4Z-MC-LYAO.pdf
- https://www.accessdata.fda.gov/drugsatfda_docs/nda/2002/21-411_Strattera.cfm
- Full CSR for both studies provided by Lilly

*Reason for exclusion: Adult participants*

**49. Moharari2012, IRCT201012295500N1**

- Moharari F, Soltanifar A, Mokhber N, Pasandideh M, Samadi R, Soltanifar A. A double-blind, randomized comparison of efficacy and side effects of bupropion versus methylphenidate in children with ADHD. *Quarterly* *Journal of Sabzevar University of Medical Sciences*, 2012; 19,3.
- Moharreri F, Mokhber N, Samadi R, Soltanifar A. Double-blind randomized comparison of efficacy and side effects of bupropion versus methylphenidate for children with ADHD. *Eur Psychiatry*. 2013;28.
- <http://www.irct.ir/searchresult.php?keyword=&id=5500&number=1&prt=1558&total=10&m=1>

*Reason for exclusion: Head to head study*

**50. NCT01069523**

- https://clinicaltrials.gov/ct2/show/NCT01069523

*Reason for exclusion: No number of participants with headaches (n) provided*

**51. Paterson1999**

- Paterson R, Douglas C, Hallmayer J, Hagan M, Krupenia Z. A randomised, double-blind, placebo-controlled trial of dexamphetamine in adults with attention deficit hyperactivity disorder. *The Aust N Z J Psychiatry*. 1999;33(4):494-502.
- Additional information provided by first author

*Reason for exclusion: Adult participants*

**52. Philipsen2015, EUCTR2006-000222-31-DE, ISRCTN54096201**

- Philipsen A, Graf E, Tebartz van Elst L, et al. Evaluation of the efficacy and effectiveness of a structured disorder tailored psychotherapy in ADHD in adults: study protocol of a randomized controlled multicentre trial. *Atten Defic* *Hyperact Disord.* 2010;2(4):203-212.
- Schlander M, Philipsen A, Schwarz O. The cost effectiveness of clinically proven treatment strategies for attentiondeficit/hyperactivity disorder (ADHD) in adult patients. *Value Health*. 2011;14 (7):A403.
- Philipsen A, Colla M, Jacob C, et al. Efficacy of psychotherapy in the treatment of adult ADHD-a randomized controlled multicentre trial. *Neuropsychiatr Enfance Adolesc.* 2012;1):S50-S51.
- Philipsen A, Graf E, Jans T, et al. A randomized controlled multicenter trial on the multimodal treatment of adult attention-deficit hyperactivity disorder: enrollment and characteristics of the study sample. *Atten Defic Hyperact* *Disord.* 2014;6(1):35-47.
- Philipsen A, Jans T, Matthies S, et al. Multimodal treatment of adult ADHD: A randomized controlled multicentretrial (COMPAS). *Atten Defic Hyperact Disord* . 2015;7:S16.
- Philipsen A, Jans T, Graf E, et al. Effects of Group Psychotherapy, Individual Counseling, Methylphenidate, and Placebo in the Treatment of Adult Attention-Deficit/Hyperactivity Disorder: A Randomized Clinical Trial. *JAMA* *Psychiatry*. 2015;72(12):1199-1210. [Erratum appears in *JAMA Psychiatry*. 2016;73(1):90; PMID: 26747290].
- van Elst LT, Maier S, Kloppel S, et al. The effect of methylphenidate intake on brain structure in adults with ADHD in a placebo-controlled randomized trial. *J Psychiatry Neurosci*. 2016;41(6):422-430.
- https://www.clinicaltrialsregister.eu/ctr-search/search?query=eudract_number:2006-000222-31
- http://isrctn.org/ISRCTN54096201
- Additional information provided by study author

*Reason for exclusion: Adult participants*

**53. Reimherr2005**

- Reimherr Frederick W. Six-week, double-blind, placebo-controlled trial of bupropion sustained release in the treatment of adults with adhd. *155^th^ Annual Meeting of the American Psychiatric Association.* 2002. http://onlinelibrary.wiley.com/o/cochrane/clcentral/articles/109/CN-00430109/frame.html.
- Reimherr FW, Hedges DW, Strong RE, Marchant BK, Williams ED. Bupropion SR in adults with ADHD: a shortterm, placebo-controlled trial. *Neuropsychiatr Dis Treat.* 2005;1(3):245-251.
- Additional information from study author

*Reason for exclusion: Adult participants*

**54. Reimherr2007**

- Reimherr FW, Williams ED, Strong RE, Mestas R, Soni P, Marchant BK. A double-blind, placebo-controlled, crossover study of osmotic release oral system methylphenidate in adults with ADHD with assessment of oppositional and emotional dimensions of the disorder. *J Clin Psychiatry.* 2007;68(1):93-101.
- Related to previous: Reimherr FW, Marchant BK, Olsen JL, Kondo D, Strong RE, Robison RJ. Serotonin Receptor Genes are Associated with Treatment Response and Symptom Severity in Adults with ADHD. *Biol* *Psychiatry.* 2010;67(9, Suppl. S):220S.
- Gale P, Reimherr FW, Marchant BK, et al. The incidence of personality disorder in adults with ADHD, and the effects on response to treatment with OROS methylphenidate (OROS MPH). *Biol Psychiatry*. 2008;63:245S.
- Post hoc analysis in: Marchant BK, Reimherr FW, Williams ED, Strong RE, Halls C, Soni P. Assessment of personality disorder in adult ADHD using data from a clinical trial of OROS methylphenidate (OROS MPH). *Biol* *Psychiatry.* 2008;63(7, Suppl. S):246S.
- Previous reference related to: Williams E, Reimherr FW, Marchant BK, Strong RE, Halls C, Soni P. Personality Disorder Assessment in Adult ADHD Utilizing Subjects Enrolled in a Clinical Trial of OROS (R) Methylphenidate (OROS (R) MPH). *J Child Adolesc Psychopharmacol*. 2008;18(6):635-636.
- Reimherr FW, Marchant BK, Olsen JL, Kondo D, Strong RE, Robison RJ. Serotonin Receptor Genes are Associated with Treatment Response and Symptom Severity in Adults with ADHD. *Biol Psychiatry.* 2010;67:220S.
- Follow-up in: Marchant BK, Reimherr FW, Halls C, Williams ED, Strong RE. OROS methylphenidate in the treatment of adults with ADHD: a 6-month, open-label, follow-up study. *Ann Clin Psychiatry.* 2010;22(3):196-204.
- Post hoc analysis in: Williams ED, Reimherr FW, Marchant BK, et al. Personality disorders in ADHD Part 1: Assessment of personality disorder in adult ADHD using data from a clinical trial of OROS methylphenidate. *Ann* *Clin Psychiatry.* 2010;22(3):84-93.
- Robison RJ, Reimherr FW, Gale PD, et al. Personality disorders in ADHD Part 2: The effect of symptoms of personality disorder on response to treatment with OROS methylphenidate in adults with ADHD. *Ann Clin* *Psychiatry.* 2010;22(2):94-102.
- Reimherr FW, Marchant BK, Williams ED, Strong RE, Halls C, Soni P. Personality disorders in ADHD Part 3: Personality disorder, social adjustment, and their relation to dimensions of adult ADHD. *Ann Clin Psychiatry.* 2010;22(2):103-112.
- Olsen JL, Reimherr FW, Marchant BK, Strong RE, Robison RJ. BDNF and TPH2 are Associated with Personality Disorder in Adults with Attention-Deficit Hyperactivity Disorder. *Biol Psychiatry.* 2010;67(9, Suppl. S):245S.
- Reimherr FW, Marchant BK, Gift TE, Steans TA, Wender PH. Types of adult attention-deficit hyperactivity disorder (ADHD): baseline characteristics, initial response, and long-term response to treatment with methylphenidate. *Atten Defic Hyperact Disord.* 2015;7(2):115-128.
- https://clinicaltrials.gov/ct2/show/NCT02215538
- Additional information from study author

*Reason for exclusion: Adult participants*

**55. Rosler2009**

- Rosler M, Fischer R, Ammer R, Ose C, Retz W. A randomised, placebo-controlled, 24-week, study of low-dose extended-release methylphenidate in adults with attention-deficit/hyperactivity disorder. *Eur Arch Psychiatry Clin* *Neurosci.* 2009;259(2):120-129.
- Post hoc analysis in: Rosler M, Retz W, Fischer R, et al. Twenty-four-week treatment with extended release methylphenidate improves emotional symptoms in adult ADHD. *World J Biol Psychiatry.* 2010;11(5):709-718.
- Post hoc analysis in: Sobanski E, Retz W, Fischer R, et al. Treatment adherence and persistence in adult ADHD: results from a twenty-four week controlled clinical trial with extended release methylphenidate. *Eur Psychiatry: the* *Journal of the Association of European Psychiatrists.* 2014;29(5):324-330.
- https://clinicaltrials.gov/ct2/show/NCT00619840
- Additional data provided by Medice

*Reason for exclusion: Adult participants*

**56. Sadramely2011**

- Sadramely MR, Karahmadi M, Azhar M, Koleini N, Farshidfar F. The effect of bupropion in treating attention deficit hyperactivity disorder in 6-17 year old children and adolescents in Isfahan. *Asian J Psychiatr.* 2011;4:S46.
- Sadramely MR, Karahmadi M, Azhar M, Koleini N, Farshidfar F. The effect of bupropion in treating attention deficit hyperactivity disorder in 6-17 year old children and adolescents in Isfahan The effect of bupropion on treating of attention deficit hyperactivity disorder in 6-17 years children and adolescents in Isfahan. Journal of Isfahan medical school 2009; 27(94). Retrieved from <https://www.cochranelibrary.com/central/doi/10.1002/central/CN-01603547/full>

*Reason for exclusion: Not written in English*

**57. Sangal2006, B4Z-US-LYAV**

- Sangal RB, Owens J, AlleRn AJ, Sutton V, Schuh K, Kelsey D. Effects of atomoxetine and methylphenidate on sleep in children with ADHD. *Sleep.* 2006;29(12):1573-1585.
- Sangal R, Owens, Allen AI, et aI. Effects of atomoxetine and methylphenidate on sleep in children with ADHD. Presented at: Associated Professional Sleep Societies Meeting, Philadelphia, June5-10, 2004

*Reason for exclusion: Head to head study*

**58. Scahill2001, NCT00004376**

- Scahill L, Chappell P, Kim YS, et al. Guanfacine in the treatment of children with tic disorders and attention deficit hyperactivity disorder: a placebo-controlled study. *39th Annual Meeting of the American College of* *Neuropsychopharmacology 2000; Dec 10-14; San Juan, Puerto Rico 2000*.
- Scahill L, Chappell PB, Kim YS, et al. A placebo-controlled study of guanfacine in the treatment of children with tic disorders and attention deficit hyperactivity disorder. *Am J Psychiatry.* 2001;158(7):1067-1074.
- <https://clinicaltrials.gov/ct2/show/NCT00004376>

*Reason for exclusion: No usable safety data*

**59. Schrantee2016, NTR3103, EUCTR2010-023654-37-NL**

- Bottelier MA, Schouw M, Klomp A, et al. The effects of Psychotropic drugs On Developing brain (ePOD) study: methods and design. *BMC Psychiatry.* 2014;14(1):48. (Protocol).
- Ferguson B, Schrantee AGM, De Ruiter MB, Bottelier MA, Reneman L. Opposite effects of acute methylphenidate administration in children versus adult ADHD patients during emotional processing. *Eur Neuropsychopharmacol.* 2014;24:S728-S729.
- Schrantee A, Tamminga HGH, Bouziane C, et al. Age-dependent effects of methylphenidate on the human dopaminergic system in young vs adult patients with attention-deficit/hyperactivity disorder: A randomized clinical trial. *JAMA Psychiatry.* 2016;73:955-962.
- Schrantee A, Tamminga HGH, Bouziane C, et al. A randomized clinical trial on the age-dependent effects of methylphenidate on the human dopaminergic system. *Biol Psychiatry.* 2016;(1):157S.
- Solleveld MM, Schrantee A, Baek HK, et al. Effects of 16 Weeks of Methylphenidate Treatment on Actigraph-Assessed Sleep Measures in Medication-Naive Children With ADHD. Front Psychiatry. 2020;(11): 82. Published online 2020 Feb 28. doi: 10.3389/fpsyt.2020.00082
- Note: Full study protocol in supplemental material of Schrantee et al. JAMA Psychiatry 2016
- http://www.trialregister.nl/trialreg/admin/rctview.asp?TC=3103 (additional ID: NL34509.000.10)
- https://www.clinicaltrialsregister.eu/ctr-search/search?query=eudract_number:2010-023654-37
- Additional information provided by first author

*Reason for exclusion: No usable satety data*

**60. Schulz2012**

- Schulz KP, Fan J, Bedard AC, et al. Common and unique therapeutic mechanisms of stimulant and nonstimulant treatments for attention-deficit/hyperactivity disorder. *Arch Gen Psychiatry.* 2012;69(9):952-961.

*Reason for exclusion: Head to head study*

**61. Simonoff2013 , ISRCTN68384912**

- Simonoff E, Taylor E, Baird G, et al. Randomized controlled double-blind trial of optimal dose methylphenidate in children and adolescents with severe attention deficit hyperactivity disorder and intellectual disability. *J Child* *Psychol Psychiatry.* 2013;54(5):527-535.
- *Commentary*: Lipkin PH. Methylphenidate reduces ADHD symptoms in children with severe ADHD and intellectual disability. *Evid Based Ment Health.* 2013;16(4):104.
- http://isrctn.org/ISRCTN68384912

*Reason for exclusion: No number of participants with headaches (n) provided*

**62. Singer1995**

- Singer HS, Brown J, Quaskey S, Rosenberg LA, Mellits ED, Denckla MB. The treatment of attention-deficit hyperactivity disorder in Tourette's syndrome: a double-blind placebo-controlled study with clonidine and desipramine. *Pediatrics*. 1995;95(1):74-81.
- Singer HS, Brown J, Quaskey S, Mellits ED, Denckla MB, Rosenberg LA. The treatment of attention-deficit hyperactivity disorder in Tourette syndrome - a double-blind placebo-controlled study with clonidine and desipramine. *Ann Neurol.* 1991;30:485-.

*Reason for exclusion: No number of participants with headaches (n) provided*

**63. Spencer1995**

- Spencer T, Wilens T, Biederman J, Faraone SV, Ablon JS, Lapey K. A double-blind, crossover comparison of methylphenidate and placebo in adults with childhood-onset attention-deficit hyperactivity disorder. *Arch Gen* *Psychiatry.* 1995;52(6):434-443.
- Wilens TE, Hammerness PG, Biederman J, et al. Blood pressure changes associated with medication treatment of adults with attention-deficit/hyperactivity disorder. *J Clin Psychiatry.* 2005;66(2):253-259.

*Reason for exclusion: Adult participants*

**64. Spencer1998**

- Spencer T, Biederman J, Wilens T, Prince J, Hacth M, Jones J, Harding M, Faraone SV, Seidman L. Effectiveness and tolerability of tomoxetine in adults with attention deficit hyperactivity disorder. *Am J Psychiatry*. 1998;155(5):693-695.

*Reason for exclusion: Adult participants*

**65. Spencer2001**

- Spencer TJ, Biederman J, Bostic JQ, Prince JB, Gerard K. Efficacy and tolerability of a mixed amphetamine salts compound in adults with adhd. 153rd *Annual Meeting of the American Psychiatric Association 2000*.
- Spencer T, Biederman J, Wilens T, et al. Efficacy of a mixed amphetamine salts compound in adults with attentiondeficit/hyperactivity disorder. *Arch Gen Psychiatry.* 2001;58(8):775-782.
- Commentary in: Newcorn JH. Amphetamine salt compound treatment for adults with attention deficit hyperactivity disorder. *Curr Psychiatry. Rep.* 2002;4(2):85-86.
- Spencer TJ. Efficacy and tolerability of a mixed amphetamine salts compound in adults with adhd. *155th Annual Meeting of the American Psychiatric Association.* 2002.
- Faraone SV, Biederman J, Spencer TJ, et al. Dose-Response Efficacy of Mixed Amphetamine Salts Extended Release in Adults With ADHD. *157th Annual Meeting of the American Psychiatric Association; 2004 May 1-6;* *New York, NY 2004:Nr440.*
- Post hoc analysis in: Wilens TE, Hammerness PG, Biederman J, et al. Blood pressure changes associated with medication treatment of adults with attention-deficit/hyperactivity disorder. *J Clin Psychiatry.* 2005;66(2):253-259.

*Reason for exclusion: Adult participants*

**66. Spencer2005**

- Spencer TJ. Preliminary results of a six-month trial of methylphenidate in adults with adhd. 156th Annual Meeting of the American Psychiatric Association, May 17-22, San Francisco CA2003:No. 54B.
- Spencer T, Biederman J, Eric M, Stephen F. Efficacy in a 6-month trial of methylphenidate inadults with attentiondeficit/hyperactivity disorder. *Int J Neuropsychopharmacol.* 2004;7:S442-S3.
- Spencer T, Biederman J, Wilens T, et al. A large, double-blind, randomized clinical trial of methylphenidate in the treatment of adults with attention-deficit/hyperactivity disorder. *Biol Psychiatry.* 2005;57(5):456-463.
- Pooled in: Mick E, Biederman J, Spencer T, Faraone SV, Sklar P. Absence of association with DAT1 polymorphism and response to methylphenidate in a sample of adults with ADHD. *Am J Med Genet B Neuropsychiatr Genet.* 2006;141B(8):890-894.
- Pooled in: Biederman J, Mick EO, Surman C, et al. Comparative acute efficacy and tolerability of OROS and immediate release formulations of methylphenidate in the treatment of adults with attention-deficit/hyperactivity disorder. *BMC Psychiatry.* 2007;7:49.
- Pooled in: Mick E, Faraone SV, Spencer T, Zhang HF, Biederman J. Assessing the validity of the Quality of Life Enjoyment and Satisfaction Questionnaire Short Form in adults with ADHD. *J Atten Disord*. Jan 2008;11(4):504-509
- Surman CBH, Monuteaux MC, Petty CR, et al. Representativeness of participants in a clinical trial for attentiondeficit/hyperactivity disorder? Comparison with adults from a large observational study. *J Clin Psychiatry.* 2010;71(12):1612-1616.

*Reason for exclusion: Adult participants*

**67. Spencer2007, CRIT124E2302**

- Spencer TJ, Kim S, Jiang H. Efficacy of Dexmethylphenidate Extended Release Capsules in Adults With ADHD. *157th Annual Meeting of the American Psychiatric Association; 2004 May 1-6; New York, NY.* 2004:Nr455.
- Adler LA, Spencer T, Wang J, Pestreich L, Muniz R. Efficacy and safety of once-daily extended-release dexmethylphenidate 30 and 40 mg in adults with ADHD: A double-blind, fixed-dose, placebo-controlled, randomized study. *Int J Neuropsychopharmacol.* 2006;9(Suppl. 1):S229.
- Spencer TJ, Adler LA, McGough JJ, Muniz R, Jiang H, Pestreich L. Efficacy and safety of dexmethylphenidate extended-release capsules in adults with attention-deficit/hyperactivity disorder. *Biol Psychiatry.* 2007;61(12):1380-1387.
- Follow-up in: Adler LA, Spencer T, McGough JJ, Jiang H, Muniz R. Long-term effectiveness and safety of dexmethylphenidate extended-release capsules in adult ADHD. *J Atten Disord.* 2009;12(5):449-459.
- https://www.novctrd.com/CtrdWeb/displaypdf.nov?trialresultid=1623 (other iD: CRIT124E2302)
- <https://www.accessdata.fda.gov/drugsatfda_docs/nda/2005/021802s000TOC.cfm>

*Reason for exclusion: Adult participants*

**68. Spencer2008, SPD465-301, NCT00150579**

- Spencer TJ, Adler LA, Weisler RH, Youcha SH. Triple-bead mixed amphetamine salts (SPD465), a novel, enhanced extended-release amphetamine formulation for the treatment of adults with ADHD: a randomized, double-blind, multicenter, placebo-controlled study. *J Clin Psychiatry.* 2008;69(9):1437-1448.
- Spencer TJ, Landgraf JM, Adler LA, Weisler RH, Anderson CS, Youcha SH. Attention-deficit/hyperactivity disorder-specific quality of life with triple-bead mixed amphetamine salts (SPD465) in adults: results of a randomized, double-blind, placebo-controlled study. *J Clin Psychiatry.* 2008;69(11):1766-1775.
- Pooled in: Brown TE, Landgraf JM. Improvements in executive function correlate with enhanced performance and functioning and health-related quality of life: evidence from 2 large, double-blind, randomized, placebo-controlled trials in ADHD. *Postgrad Med.* 2010;122(5):42-51.
- Secondary analysis in: Surman CB, Roth T. Impact of stimulant pharmacotherapy on sleep quality: post hoc analyses of 2 large, double-blind, randomized, placebo-controlled trials. *J Clin Psychiatry.* 2011;72(7):903-908.
- https://clinicaltrials.gov/ct2/show/NCT00150579 (other ID: SPD465-301) Surman, C. B. H., Robertson, B., Chen, J., & Cortese, S. (2019). Post-Hoc Analyses of the Effects of Baseline Sleep Quality on SHP465 Mixed Amphetamine Salts Extended-Release Treatment Response in Adults with Attention-Deficit/Hyperactivity Disorder. CNS Drugs. doi:10.1007/s40263-019-00645-z
- Additional data provided by Shire

*Reason for exclusion: Adult participants*

**69. Stein2011, NCT00393042**

- Stein MA, Waldman ID, Sarampote C, Seymour K, Cook EH. Dopamine transporter genotype (DAT1) predicts stimulant response in children with attention deficit hyperactivity disorder. *Pediatr Res*. 2004;55:1A.
- Wiebe S, Gruber R, Charney E, Aryal S, Waldman I, Newcorn J, et al. Sleep and emotional reactivity to extended release dexmethylphenidate versus mixed amphetamine salts: a double-blind, placebo controlled study. European Child & Adolescent Psychiatry. Proceedings of the Eunethydis 1st International ADHD Conference: From Data to Best Clinical Practice; 2010 May 26-28; Amsterdam, Netherlands 2010;19(Suppl 1):S82
- Stein MA, Waldman ID, Charney E, et al. Dose effects and comparative effectiveness of extended release dexmethylphenidate and mixed amphetamine salts. *J Child Adolesc Psychopharmacol.* 2011;21(6):581-588.
- Santisteban JA, Stein MA, Bergmame L, Gruber R. Effect of extended-release dexmethylphenidate and mixed amphetamine salts on sleep: a double-blind, randomized, crossover study in youth with attention-deficit hyperactivity disorder. *CNS Drugs.* 2014;28(9):825-833. (NCT00393042)
- Santisteban JA, Stein MA, Gruber R. Effects of dosage on sleep duration during stimulant treatment of ADHD in youth. *Paediatr Child Health (Canada).* 2014;19 (6):e54-e55.
- Stein MA, Waldman I, Newcorn J, Bishop J, Kittles R, Cook EH, Jr. Dopamine transporter genotype and stimulant dose-response in youth with attention-deficit/hyperactivity disorder. *J Child Adolesc Psychopharmacol.* 2014;24(5):238-244.
- Conference proceeding related to previous reference: Newcorn J, Stein M. Dopamine transporter genotype and dose-response following treatment with psychostimulants: Implications for optimization of acute and longer-term treatment. *Atten Defic Hyperact Disord.* 2015;7:S67.
- https://clinicaltrials.gov/ct2/show/NCT00393042

*Reason for exclusion: Cross-over without wash out*

**70. Sutherland2012, NCT00174226**

- Sutherland SM, Adler LA, Chen C, Smith MD, Feltner DE. An 8-week, randomized controlled trial of atomoxetine, atomoxetine plus buspirone, or placebo in adults with ADHD. *J Clin Psychiatry.* 2012;73(4):445-450.
- <https://clinicaltrials.gov/ct2/show/NCT00174226>

*Reason for exclusion: Adult participants*

**71. Takahashi2014, NCT01323192**

- Takahashi N, Koh T, Tominaga Y, Saito Y, Kashimoto Y, Matsumura T. A randomized, double-blind, placebocontrolled, parallel-group study to evaluate the efficacy and safety of osmotic-controlled release oral delivery system methylphenidate HCl in adults with attention-deficit/hyperactivity disorder in Japan. *World J Biol* *Psychiatry.* 2014.
- https://clinicaltrials.gov/ct2/show/NCT01323192
- <https://yoda.yale.edu/sites/default/files/nct01323192.pdf>

*Reason for exclusion: Adult participants*

**72. Taylor1987**

- Taylor E, Schachar R, Thorley G, Wieselberg HM, Everitt B, Rutter M. Which boys respond to stimulant medication? A controlled trial of methylphenidate in boys with disruptive behaviour. *Psychol Med.* 1987;17(1):121-143.
- Schachar R, Taylor E, Wieselberg M, Thorley G, Rutter M. Changes in family function and relationships in children who respond to methylphenidate. *J Am Acad Child Adolesc Psychiatry.* 1987;26(5):728-732.

*Reason for exclusion: No number of participants with headaches (n) provided*

**73. Taylor2000**

- Taylor FB, Russo J. Efficacy of modafinil compared to dextroamphetamine for the treatment of attention deficit hyperactivity disorder in adults. *J Child Adolesc Psychopharmacol.* 2000;10(4):311-320.
- Taylor IF. Comparing modafinil to dextroamphetamine in the treatment of adult adhd. *153rd Annual Meeting of the American Psychiatric Association.* 2000.
- Taylor FB. Comparing modafinil to dextroamphetamine in the treatment of adult adhd. *155th Annual Meeting of the American Psychiatric Association.* 2002.

*Reason for exclusion: Adult participants*

**74. Taylor2001**

- Taylor FB, Russo J. Comparing guanfacine and dextroamphetamine for the treatment of adult attentiondeficit/hyperactivity disorder. *J Clin Psychopharmacol.* 2001;21(2):223-228. Taylor IF. Comparing guanfacine and dextroamphetamine for adult adhd: efficacy and implications. *153rd Annual Meeting of the American Psychiatric Association.* 2000.
- Taylor FB. Comparing guanfacine and dextroamphetamine for adult adhd: efficacy and implications. 155th Annual Meeting of the American Psychiatric Association 2002

*Reason for exclusion: Adult participants*

**75. van der Meere1999**

- van der Meere J, Gunning B, Stemerdink N. The effect of methylphenidate and clonidine on response inhibition and state regulation in children with ADHD. *J Child Psychol Psychiatry.* 1999;40(2):291-298.
- Gunning WB. A Controlled Trial of Clonidine in Hyperkinetic Children. Erasmus Universiteit Rotterdam, 1992. <http://repub.eur.nl/res/pub/8339/920311_Gunning,%20Willem%20Boudewijn.pdf>

*Reason for exclusion: No number of participants with headaches (n) provided*

**76. Wang2007, NCT00486083, B4Z-MC-LYBR (6934)**

- Levine L, Wang Y, Zheng Y, Du Y, Cho SC, Gao H, Marquez-Caraveo ME, Rogers AK, Williams DW. Atomoxetine vs. methylphenidate in ADHD treatment: an international pediatric trials. *Eur Neuropsychopharamacol* 2005, 15, Suppl 3, S602.
- Wang Y, Zheng Y, Du Y, et al. Atomoxetine versus methylphenidate in paediatric outpatients with attention deficit hyperactivity disorder: a randomized, double-blind comparison trial. *The Aust N Z J Psychiatry.* 2007;41(3):222-230.
- https://clinicaltrials.gov/ct2/show/NCT00486083
- https://assets.contentful.com/hadumfdtzsru/4RGQpHv0BOWMMcCcAyii2s/a91d17111b5aa1326bef55e28cf7cc06/Atomoxetine-B4Z-MC-LYBR.pdf
- https://www.accessdata.fda.gov/drugsatfda_docs/nda/2002/21-411_Strattera.cfm

*Reason for exclusion: Head to head study*

**77. Weisler2006, SLI381-303**

- Weisler RH, Biederman J, Chrisman AK, Timothy TP, Frazer N, Tulloch SJ. Long-Term Safety and Efficacy of Once-Daily Adderall Extended Release in Adults With ADHD. 156th Annual Meeting of the American Psychiatric Association, May 17-22, San Francisco CA2003:Nr647.
- Weisler RH, Chrisman AK, Wilens TE. Adderall xr dosed once daily in adult patients with adhd. 156th *Annual Meeting of the American Psychiatric Association, May 17-22, San Francisco CA. 2003:No. 33*. http://onlinelibrary.wiley.com/o/cochrane/clcentral/articles/838/CN-00592838/frame.html.
- Long-term follow-up in: Biederman J, Spencer TJ, Wilens TE, Weisler RH, Read SC, Tulloch SJ. Long-term safety and effectiveness of mixed amphetamine salts extended release in adults with ADHD. *CNS Spectr.* 2005;10(12 Suppl 20):16-25.
- Weisler RH, Biederman J, Spencer TJ, Wilens TE. Long-term cardiovascular effects of mixed amphetamine salts extended release in adults with ADHD. *CNS Spectr.* 2005;10(12 Suppl 20):35-43.
- Weisler RH, Biederman J, Spencer TJ, et al. Mixed amphetamine salts extended-release in the treatment of adult ADHD: a randomized, controlled trial. *CNS Spectr.* 2006;11(8):625-639.
- Pooled in: Lasser R, Dirks B, Adeyi B, Babcock T. Comparative efficacy and safety of lisdexamfetamine dimesylate and mixed amphetamine salts extended release in adults with attention-deficit/hyperactivity disorder. *Prim* *psychiatry.* 2010;17(9):44-54.
- Additional data provided by Shire (ID: SLI381-303)

*Reason for exclusion: Adult participants*

**78. Weisler2012, NCT00880217**

- Weisler RH, Pandina GJ, Daly EJ, Cooper K, Gassmann-Mayer C, Investigato ATTS. Randomized Clinical Study of a Histamine H-3 Receptor Antagonist for the Treatment of Adults with Attention-Deficit Hyperactivity Disorder. *CNS* *Drugs*. 2012;26(5):421-434.
- https://clinicaltrials.gov/ct2/show/NCT00880217 (additional ID: 31001074-ATT2001**)**
- Additional information from manufacturer

*Reason for exclusion: Adult participants*

**79. Weisler2017, NCT02604407**

- Weisler RH, Greenbaum M, Arnold V, Yu M, Yan B, Jaffee M, Robertson B. Efficacy and Safety of SHP465 Mixed Amphetamine Salts in the Treatment of Attention-Deficit/Hyperactivity Disorder in Adults: Results of a Randomized, Double-Blind, Placebo-Controlled, Forced-Dose Clinical Study. CNS Drugs. 2017;31(8):685-697.
- <https://clinicaltrials.gov/ct2/show/NCT02604407>

*Reason for exclusion: Adult participants*

**80. Weiss2005, B4Z-MC-LYAW**

- Weiss M, Tannock R, Kratochvil C, et al. Placebo-controlled study of once-daily atomoxetine in the school setting. *Eur Neuropsychopharmacol.* 2003;13(Supplement 4):S456-S457.
- Pooled in: Perwien AR, Faries DE, Kratochvil CJ, Sumner CR, Kelsey DK, Allen AJ. Improvement in health-related quality of life in children with ADHD: an analysis of placebo controlled studies of atomoxetine. *J Dev Behav* *Pediatr.* 2004;25(4):264-271.
- Weiss M, Tannock R, Kratochvil C, et al. A randomized, placebo-controlled study of once-daily atomoxetine in the school setting in children with ADHD. *J Am Acad Child Adolesc Psychiatry.* 2005;44(7):647-655.
- Bohnstedt BN, Kronenberger WG, Dunn DW, et al. Investigator ratings of ADHD symptoms during a randomized, placebo-controlled trial of atomoxetine: a comparison of parents and teachers as informants. *J Atten Disord.* 2005;8(4):153-159.
- Brown RT, Perwien A, Faries DE, Kratochvil CJ, Vaughan BS. Atomoxetine in the management of children with ADHD: effects on quality of life and school functioning. *Clin Pediatr (Phila).* 2006;45(9):819-827.
- Commentary: Barton J. Atomoxetine improves teacher rated symptoms in children with ADHD more than placebo. *Evid Based Ment Health.* 2006;9(1):7.
- Pooled in: Biederman J, Gao H, Rogers AK, Spencer TJ. Comparison of parent and teacher reports of attentiondeficit/hyperactivity disorder symptoms from two placebo‐controlled studies of atomoxetine in children. *Biol* *Psychiatry.* 2006;60(10):1106‐1110.
- Pooled in: Biederman J, Spencer TJ, Newcorn JH, et al. Effect of comorbid symptoms of oppositional defiant disorder on responses to atomoxetine in children with ADHD: a meta-analysis of controlled clinical trial data. *Psychopharmacology (Berl).* 2007;190:31-41
- Related to previous reference: Biederman J, Spencer T, Newcorn J, Gao H, Milton D, Feldman P. Does the Presence of Comorbid ODD Affect Responses to Atomoxetine? 158th Annual Meeting of the American Psychiatric Association; 2005 May 21-26; Atlanta, GA2005.
- https://assets.contentful.com/hadumfdtzsru/1PT69wZkT2UosACGuAImuw/f80b9521e50d1cb2dce4fcd31c96d845/Atomoxetine-B4Z-MC-LYAW_a_.pdf
- https://assets.contentful.com/hadumfdtzsru/28G1mHvyIkSqww8w4AueWU/e99d85ebd3c15c789c20791f3d52410f/Atomoxetine-B4Z-MC-LYAW_b_.pdf
- https://www.accessdata.fda.gov/drugsatfda_docs/nda/2002/21-411_Strattera.cfm

*Reason for exclusion: No number of participants with headaches (n) provided*

**81. Weiss 2020**

- Weiss M, Childress AC, Donnelly GAE. Efficacy and safety of PRC-063, extended release multilayer methylphenidate in adults with ADHD including 6-month open-label extension. *J Attention Disorders*. 2020:

*Reasons for excludion: Adult participants*

**82. Wender2011**

- Wender PH, Reimherr FW, Marchant B, Czajkowski L, Sanford ME. A placebo-controlled, long-term trial of methylphenidate in the treatment of adults with ADHD. *2001 Annual Meeting of the American Psychiatric* *Association.* 2001.
- Wender Paul H. A placebo-controlled, long-term trial of methylphenidate in the treatment of adults with adhd. *155^th^ Annual Meeting of the American Psychiatric Association 2002.*
- Wender PH, Szajkowski L, Marchant B, Reimherr FW, Sanford E, Eden J. A Long-Term Study of Methylphenidate in the Treatment of ADHD in Adults. *156th Annual Meeting of the American Psychiatric Association, May 17-22,* *San Francisco CA2003:Nr708.*
- Wender PH, Reimherr FW, Marchant B, Sanford E, Czajkowski L. A Long-Term Trial of Methylphenidate in the Treatment of ADHD in Adults. 156th Annual Meeting of the American Psychiatric Association, May 17-22, San Francisco CA. 2003:Nr709.
- Wender PH, Reimherr FW, Marchant BK, Sanford ME, Czajkowski LA, Tomb DA. A one year trial of methylphenidate in the treatment of ADHD. *J Atten Disord.* 2011;15(1):36-45.
- https://clinicaltrials.gov/ct2/show/NCT00693212
- Additional data from study author

*Reason for exclusion: Adult participants*

**83. Wilens2001**

- Wilens TE, Spencer TJ, Biederman J, et al. A controlled clinical trial of bupropion for attention deficit hyperactivity disorder in adults. *Am J Psychiatry.* 2001;158(2):282-288.
- Commentary in: Ferre, J.C. & Nutt, D. (2001). Bupropion improved symptoms in adults with attention deficit hyperactivity disorder. Evidence Based Mental Health, 158, 282-288
- Commentary in: Newcorn JH. Bupropion for adults with attention deficit hyperactivity disorder. *Curr Psychiatry Rep.* 2002;4(2):86-87.
- Post hoc analysis in: Wilens TE, Hammerness PG, Biederman J, et al. Blood pressure changes associated with medication treatment of adults with attention-deficit/hyperactivity disorder. *J Clin Psychiatry.* 2005;66(2):253-259.

*Reason for exclusion: Adult participants*

**84. Wilens2005, NCT00048360**

- Wilens TE, Hudziak JJ, Connor DF, et al. A Controlled Trial of Extended-Release Bupropion in Adult ADHD. *157th Annual Meeting of the American Psychiatric Association; 2004 May 1-6; New York, NY2004:Nr576*.
- Wilens TE, Haight BR, Horrigan JP, et al. Bupropion XL in adults with attention-deficit/hyperactivity disorder: a randomized, placebo-controlled study. *Biol Psychiatry.* 2005;57(7):793-801.
- https://clinicaltrials.gov/ct2/show/NCT00048360 (other ID: AK130934)

*Reason for exclusion: Adult participants*

**85. Wilens2008, B4Z-MC-LYBY, NCT00190957**

- Wilens TE, Adler LA, Weiss MD, Ramsey JL, Moore RF, Renard D, Trzepacz PT, Schuh LM, Dittmann RW, Levine LR, No. Atomoxetine treatment of adults with ADHD and comorbid alcohol abuse disorder. *Pharmacopsychiatry*. 2007; 40: 150
- Wilens TE, Adler LA, Weiss MD, et al. Atomoxetine treatment of adults with ADHD and comorbid alcohol abuse*. Proceedings of the 69th Annual Scientific Meeting of the College on Problems of Drug Dependence; 2007 June 16-21; Quebec City, Canada. 2007*.
- Wilens TE, Adler LA, Weiss MD, et al. Atomoxetine treatment of adults with ADHD and comorbid alcohol use disorders. *Drug Alcohol Depend.* 2008;96(1-2):145-154.
- Pooled in: Adler L, Wilens T, Zhang S, et al. Retrospective safety analysis of atomoxetine in adult ADHD patients with or without comorbid alcohol abuse and dependence. *Am J Addict.* 2009;18(5):393-401
- Pooled in: Reimherr FW, Olsen J, Marchant BK, et al. ADHD Symptoms Associated with Comorbid Alcohol Dependence Or Abuse: Comparison of Subject Attributes within ADHD Clinical Trials*. Biol Psychiatry*. 2009;65:116S.
- Post hoc analysis in: Wilens TE, Adler LA, Tanaka Y, et al. Correlates of alcohol use in adults with ADHD and comorbid alcohol use disorders: exploratory analysis of a placebo-controlled trial of atomoxetine. *Curr Med Res* *Opin.* 2011;27(12):2309-2320.
- https://clinicaltrials.gov/ct2/show/NCT00190957
- https://assets.contentful.com/hadumfdtzsru/1PXcFgjDCAW6EUOYMg4I80/da1e7c4d91490b68c9fd23385bdc3920/Atomoxetine-B4Z-MC-LYBY.pdf
- Full CSR provided by Lilly

*Reason for exclusion: Adult participants*

**86. Wietecha2013, NCT00607919**

- Shaywitz SE, Shaywitz BA, Wietecha LA, et al. Effects of atomoxetine on reading abilities in children with dyslexia and children with attention deficit/hyperactivity disorder and comorbid dyslexia. *Ann Neurol.* 2012;72:S204.
- Wietecha L, Williams D, Shaywitz S, et al. Atomoxetine improved attention in children and adolescents with attention-deficit/hyperactivity disorder and dyslexia in a 16 week, acute, randomized, double-blind trial. *J Child* *Adolesc Psychopharmacol.* 2013;23(9):605-613.
- Post-hoc analysis in: McBurnett K, Clemow D, Williams D, Villodas M, Wietecha L, Barkley R. Atomoxetine- Related Change in Sluggish Cognitive Tempo Is Partially Independent of Change in Attention-Deficit/Hyperactivity Disorder Inattentive Symptoms. *J Child Adolesc Psychopharmacol.* 2016;27:38-42.
- Shaywitz S, Shaywitz B, Wietecha L, et al. Effect of Atomoxetine Treatment on Reading and Phonological Skills in Children with Dyslexia or Attention-Deficit/Hyperactivity Disorder and Comorbid Dyslexia in a Randomized, Placebo-Controlled Trial. *J Child Adolesc Psychopharmacol.* 2016;27(1):19-28.
- https://clinicaltrials.gov/ct2/show/NCT00607919

*Reason for exclusion: Safety data including participants without ADHD diagnosis*

**87. Wigal 2005, SLI381-404, NCT00506727**

- Wigal S, McGough JJ, Posner K, Kollins SH, Michaels A, Tulloch SJ. Analog classroom study of amphetamine extended release and atomoxetine in youth with ADHD. *157th Annual Meeting of the American Psychiatric* *Association; 2004 May 1-6; New York, NY.* 2004.
- Wigal SB, McGough JJ, McCracken JT, et al. A laboratory school comparison of mixed amphetamine salts extended release (Adderall XR) and atomoxetine (Strattera) in school-aged children with attention deficit/hyperactivity disorder. *J Atten Disord.* 2005;9(1):275-289.
- Data on girls only in: Biederman J, Wigal SB, Spencer TJ, McGough JJ, Mays DA. A post hoc subgroup analysis of an 18-day randomized controlled trial comparing the tolerability and efficacy of mixed amphetamine salts extended release and atomoxetine in school-age girls with attention-deficit/hyperactivity disorder. Clin Ther. Feb 2006;28(2):280-293.
- Post hoc analysis in: Faraone SV, Wigal SB, Hodgkins P. Forecasting three-month outcomes in a laboratory school comparison of mixed amphetamine salts extended release (Adderall XR) and atomoxetine (Strattera) in school-aged children with ADHD. *J Atten Disord.* 2007;11(1):74-82.
- https://clinicaltrials.gov/ct2/show/NCT00506727 (other ID: SLI381-404**)**

*Reason for exclusion: Head to head study*

**88. Wilens2011, NCT00528697**

- Wilens TE, Gault LM, Childress A, et al. Safety and efficacy of ABT-089 in pediatric attention-deficit/hyperactivity disorder: results from two randomized placebo-controlled clinical trials. *J Am Acad Child Adolesc Psychiatry.* 2011;50(1):73-84 e71
- <https://clinicaltrials.gov/ct2/show/NCT00528697>

*Reason for exclusion: No pertinent ADHD medication in the present systematic review*

**89. Winhusen2010, NCT00253747**

- Winhusen TM, Somoza EC, Brigham GS, et al. Impact of attention-deficit/hyperactivity disorder (ADHD) treatment on smoking cessation intervention in ADHD smokers: a randomized, double-blind, placebo-controlled trial. *J Clin* *Psychiatry.* 2010;71(12):1680-1688.
- Covey LS, Hu MC, Winhusen T, Weissman J, Berlin I, Nunes EV. OROS-methylphenidate or placebo for adult smokers with attention deficit hyperactivity disorder: racial/ethnic differences. *Drug Alcohol Depend.* 2010;110(1-2):156-159.
- Covey LS, Hu MC, Weissman J, Croghan I, Adler L, Winhusen T. Divergence by ADHD subtype in smoking cessation response to OROS-methylphenidate. *Nicotine Tob Res.* 2011;13(10):1003-1008.
- Luo SX, Wall M, Covey L, Hu MC, Scodes JM, Levin FR, Nunes EV, Winhusen T. Exploring longitudinal course and treatment-baseline severity interactions in secondary outcomes of smoking cessation treatment in individuals with attention-deficit hyperactivity disorder. Am J Drug Alcohol Abuse. 2018;44(6):653-659.
- Pooled in: Winhusen TM, Lewis DF, Riggs PD, et al. Subjective effects, misuse, and adverse effects of osmoticrelease methylphenidate treatment in adolescent substance abusers with attention-deficit/hyperactivity disorder. *J* *Child Adolesc Psychopharmacol.* 2011;21(5):455-463.
- Berlin I, Hu MC, Covey LS, Winhusen T. Attention-deficit/hyperactivity disorder (ADHD) symptoms, craving to smoke, and tobacco withdrawal symptoms in adult smokers with ADHD. *Drug Alcohol Depend.* 2012;124(3):268-273.
- Westover AN, Nakonezny PA, Winhusen T, Adinoff B, Vongpatanasin W. Risk of methylphenidate-induced prehypertension in normotensive adult smokers with attention deficit hyperactivity disorder. *J Clin Hypertens* *(Greenwich, Conn.).* 2013;15(2):124-132.
- Post hoc analysis in: Nunes EV, Covey LS, Brigham G, et al. Treating nicotine dependence by targeting attentiondeficit/hyperactivity disorder (ADHD) with OROS methylphenidate: the role of baseline ADHD severity and treatment response. *J Clin Psychiatry.* 2013;74(10):983-990.
- Post hoc analysis in: Heffner JL, Lewis DF, Winhusen TM. Osmotic release oral system methylphenidate prevents weight gain during a smoking-cessation attempt in adults with ADHD. *Nicotine Tob Res: official journal of the* *Society for Research on Nicotine and Tobacco.* 2013;15(2):583-587.
- Post hoc analysis in: Heffner JL, Lewis DF, Winhusen TM. Preliminary evidence that adherence to counseling mediates the effects of pretreatment self-efficacy and motivation on outcome of a cessation attempt in smokers with ADHD. *Nicotine Tob Res: official journal of the Society for Research on Nicotine and Tobacco.* 2013;15(2):393-400.
- Secondary analysis in: Luo, S.X., Covey, L., Hu, M.C., Levin, F.R., & Nunes, E.V. (2013). Predictive modeling and nonlinear treatment effects in a multicenter, randomized controlled trial of methylphenidate in smoke cessation intervention. *Am J Addict*. 22 (3), 305.
- Secondary analysis in: Luo SX, Covey LS, Hu MC, Levin FR, Nunes EV, Winhusen TM. Toward personalized smoking-cessation treatment: Using a predictive modeling approach to guide decisions regarding stimulant medication treatment of attention-deficit/hyperactivity disorder (ADHD) in smokers. *Am J Addict.* 2015;24(4):348-356.
- Luo SX, Wall MM, Covey LS, et al. Modeling potential mechanisms of differential treatment effects in osmoticrelease methylphenidate for smoking cessation. *Drug Alcohol Depend.* 2015;146:e188.
- https://clinicaltrials.gov/ct2/show/NCT00253747
- Additional information from study authors

*Reason for exclusion: Adult participants*

**90. Young2011, B4Z-US-LYCW, NCT00190775**

- Young JL, Sarkis E, Qiao M, Wietecha L. Once-daily treatment with atomoxetine in adults with attentiondeficit/hyperactivity disorder: a 24-week, randomized, double-blind, placebo-controlled trial. *Clin Neuropharmacol.* 2011;34(2):51-60.
- Pooled in: Wietecha L, Young J, Ruff D, Dunn D, Findling RL, Saylor K. Atomoxetine once daily for 24 weeks in adults with attention-deficit/hyperactivity disorder (ADHD): impact of treatment on family functioning. *Clin Neuropharmacol.* 2012;35(3):125-133.
- Wietecha LA, Clemow DB, Buchanan AS, Young JL, Sarkis EH, Findling RL. Atomoxetine Increased Effect over Time in Adults with Attention-Deficit/Hyperactivity Disorder Treated for up to 6 Months: Pooled Analysis of Two Double-Blind, Placebo-Controlled, Randomized Trials. *CNS Neurosci Ther.* 2016;22(7):546-557.
- https://clinicaltrials.gov/ct2/show/NCT00190775 (other ID: B4Z-US-LYCW(9043)
- Full CSR provided by Lilly

*Reason for exclusion: Adult participants*

**B. From the list of discarded trials in the updated search**

1. **Aharonovich2006**

- Aharonovich E, Garawi F, Bisaga A, et al. Concurrent cannabis use during treatment for comorbid ADHD and cocaine dependence: effects on outcome. *Am J Drug Alcohol Abuse.* 2006;32(4):629-635.

*Reason for exclusion: Concurrent CBT Adult participants*

1. **Altszuler2017**

- Altszuler AR, Morrow AS, Merrill BM, et al. The Effects of Stimulant Medication and Training on Sports Competence Among Children With ADHD. *J Clin Child Adolesc Psychol.* 2017:1-13.

*Reason for exclusion: No outcomes of interest; concomitant sport training*

*No safety data*

1. **Beery2013**

- Beery SH, Quay HC, Pelham WE, Jr. Differential Response to Methylphenidate in Inattentive and Combined Subtype ADHD. *J Atten Disord.* 2017;21(1):62-7

*Reason for exclusion: Concomitant behavioural treatment No safety data*

1. **Borcherding1989**

- Borcherding BG, Keysor CS, Cooper TB, Rapoport JL. Differential effects of methylphenidate and dextroamphetamine on the motor activity level of hyperactive children. *Neuropsychopharmacology.* 1989;2(4):255-263.
- Elia J, Borcherding BG, Potter WZ, Mefford IN, Rapoport JL, Keysor CS. Stimulant drug treatment of hyperactivity: biochemical correlates. *Clin Pharmacol Ther*. 1990;48(1):57-66
- Borcherding BG, Keysor CS, Rapoport JL, Elia J, Amass J. Motor/vocal tics and compulsive behaviors on stimulant drugs: is there a common vulnerability? *Psychiatry Res.*1990;33(1):83-94
- Sharp WS,Walter JM,MarshWL, Ritchie GF,HamburgerSD, Elia J, Borcherding BG, Potter WZ, Mefford IN, Rapoport JL, Keysor CS. Stimulant drug treatment of hyperactivity: biochemical correlates. *Clin PharmacolTher.* 1990;48(1):57-66.
- Elia J, Borcherding BG, Rapoport JL, Keysor CS. Methylphenidate and dextroamphetamine treatments of hyperactivity: are there true nonresponders? *Psychiatry Res.* 1991;36(2):141-155.
- Elia J, Welsh PA, Gullotta CS, Rapoport JL. Classroom academic performance: improvement with both methylphenidate and dextroamphetamine in ADHD boys. *J Child Psychol Psychiatry.* 1993;34(5):785-804.
- Schmidt ME, Kruesi MJ, Elia J, et al. Effect of dextroamphetamine and methylphenidate on calcium and magnesium concentration in hyperactive boys. *Psychiatry Res.* 1994;54(2):199-210
- Castellanos FX, Elia J, Kruesi MJP, et al. Cerebrospinal fluid homovanillic acid predicts behavioral response to stimulants in 45 boys with attention deficit hyperactivity disorder. *Neuropsychopharmacology.* 1996;14(2):125-137.
- Castellanos FX, Giedd JN, Elia J, et al. Controlled stimulant treatment of ADHD and comorbid Tourette's syndrome: effects of stimulant and dose. *J Am Acad Child Adolesc Psychiatry.* 1997;36(5):589-596.
- Castellanos FX. ADHD in girls: clinical comparability of a research sample. *J Am Acad Child Adolesc Psychiatry.* 1999;38(1):40–7

*Reason for exclusion: Co-treatment (therapeutic art) No safety data*

1. **Borden1989**

- Borden KA, Brown RT. Attributional outcomes: The subtle messages of treatments for attention deficit disorder. *Cognit Ther Res*. 1989(2):147-160.

*Reason for exclusion: No arms of interest for the present meta-analysis (cognitive training, methylphenidate+cognitive training, cognitive training + placebo) No safety data*

1. **Brown1985c**

- Brown RT, Wynne ME, Medenis R. Methylphenidate and cognitive therapy: A comparison of treatment approaches with hyperactive boys. *J Abnorm Child Psychol.* 1985(1):69-87.

*Reason for exclusion: No arms of interest for the present meta-analysis (methylphenidate, cognitive training, cognitive training plus methylphenidate, no treatment) No safety data*

1. **Brown1986a**

- Brown RT, Borden KA, Wynne ME, Schleser R, Clingerman SR. Methylphenidate and cognitive therapy with ADD children: a methodological reconsideration. *J Abnorm Child Psychol.* 1986;14(4):481-497.

*Reason for exclusion: No arms of interests for the present meta-analysis: methylphenidate, cognitive training, cognitive training plus methylphenidate, no treatment No safety data*

1. **Brown1986b**

- Brown RT, Wynne ME, Borden KA, Clingerman SR, Geniesse R, Spunt AL. Methylphenidate and cognitive therapy in children with attention deficit disorder: a double-blind trial. *J Dev Behav Pediatr.* 1986;7(3):163-174.

*Reason for exclusion: not appropriate design No safety data*

1. **Brown1987**

- Brown RT, Borden KA, Wynne ME. Compliance with pharmacological and cognitive treatments for attention deficit

disorder. *J Am Acad Child Adolesc Psychiatry.* 1987;26(4):521-526.

*Reason for exclusion: Concurrent additional treatments No safety data*

1. **Brown1988a**

- Brown RT, Borden KA, Wynne ME, Spunt AL, Clingerman SR. Patterns of compliance in a treatment program for children with attention deficit disorder. *J. Compliance Health Care.* 1988(1):23-39.

*Reason for exclusion: No pertinent arms for the present meta-analysis ((1) cognitive therapy plus placebo, (2) cognitive therapy plus methylphenidate, (3) methylphenidate plus attention control, or (4) placebo plus attention control) No safety data*

1. **Bukstein1998**

- Bukstein OG, Kolko DJ. Effects of methylphenidate on aggressive urban children with attention deficit hyperactivity disorder. *J Clin Child Psychol.* 1998;27(3):340-351.

*Reason for exclusion: Additional behavioral treatment component No cases (n) of side effects provided*

1. **Carpentier2005**

- Carpentier PJ, de Jong CA, Dijkstra BA, Verbrugge CA, Krabbe PF. A controlled trial of methylphenidate in adults with attention deficit/hyperactivity disorder and substance use disorders. *Addiction.* 2005;100(12):1868-1874.

*Reason for exclusion: Co-treatment during trial Adult participants*

1. **Conners1996a**

- Conners CK, Levin ED, Sparrow E, et al. Nicotine and attention in adult attention deficit hyperactivity disorder (ADHD). *Psychopharmacol Bull.* 1996;32(1):67-73.

*Reason for exclusion: Transdermal formulations Adult participants*

1. **Corkum 2020**

- Corkum P, Begum EA, Rusak B, et al. The Effects of Extended-Release Stimulant Medication on Sleep in Children with ADHD. J Can Acad Child Adolesc Psychiatry. 2020;29:33-43.

*Reason for exclusion: no outome of interest No outcome of interest*

1. **CTRI/2017/01/007665**

- http://www.ctri.nic.in/Clinicaltrials/pmaindet2.php?trialid=14324

*Reasons for exclusion: Atomoxetine vs psychological intervention vs combination Not RCT*

1. **Diamond1999**

- Diamond IR, Tannock R, Schachar RJ. Response to methylphenidate in children with ADHD and comorbid anxiety. *J Am Acad Child Adolesc Psychiatry.* 1999;38(4):402-409.

*Reason for exclusion: Co-intervention No useful data for headache measurement*

1. **Döpfner2004b**

- Dopfner M, Breuer D, Schurmann S, Metternich TW, Rademacher C, Lehmkuhl G. Effectiveness of an adaptive multimodal treatment in children with Attention-Deficit Hyperactivity Disorder - Global outcome. *Eur Child* *Adolesc Psychiatry, Supplement.* 2004;13(1):I/117-I/129.

*Reason for exclusion: No arms of interest for the present meta-analysis (methylphenidate + behavioural training vs.behavioural training No safety data*

1. **Duric2012**

- Duric NS, Assmus J, Gundersen D, Elgen IB. Neurofeedback for the treatment of children and adolescents with ADHD: a randomized and controlled clinical trial using parental reports. *BMC Psychiatry.* 2012;12:107.

*Reason for exclusion: No arms of interest for the present meta-analysis (methylphenidate, neurofeedback, methylphenidate +neurofeedback) No safety data*

1. **EUCTR2010-019981-94-FI**

- https://www.clinicaltrialsregister.eu/ctr-search/search?query=eudract_number:2010-019981-94

*Reasons for exclusion: Co-treatment Adult participants*

1. **Findling2001**

- Findling RL, Short EJ, Manos MJ. Short-term cardiovascular effects of methylphenidate and adderall. *J Am Acad Child Adolesc Psychiatry.* 2001;40(5):525-529.

*Reason for exclusion: Clinician blinded to dose but not identity of the medication*

*Head to head study*

1. **Firestone1981**

- Firestone P. Differential Effects of Parent Training and Stimulant Medication with Hyperactives: A Progress Report. *Children's Hospital of Eastern Ontario, Ottawa (Canada). 1979.*
- Firestone P, Kelly MJ, Goodman JT, Davey J. Differential effects of parent training and stimulant medication with hyperactives: a progress report. *J Am Acad Child Psychiatr*. 1981;20(1): 135–47.

*Reason for exclusion: No appropriate arms for the present meta-analysis No safety data*

1. **Firestone1986**

- Firestone P, Crowe D, Goodman JT, McGrath P. Vicissitudes of follow-up studies: differential effects of parent training and stimulant medication with hyperactives. *Am J Orthopsychiatry.* 1986;56(2):184-194.

*Reason for exclusion: Study arms (parent training +medication; parent training plus placebo; medicatin only) not appropriate for the present meta-analysis No safety data*

1. **Gittelman-Klein1976b**

- Gittelman-Klein R, Klein DF, Abikoff H, Katz S, Gloisten AC, Kates W. Relative efficacy of methylphenidate and behavior modification in hyperkinetic children: an interim report. *J Abnorm Child Psychol.* 1976;4(4):361-379.

*Reason for exclusion: No study arms of interest for the present meta-analysis No safety data*

1. **Granger1996**

- Granger DA, Whalen CK, Henker B, et al. ADHD boys' behavior during structured classroom social activities: Effects of social demands, teacher proximity, and methylphenidate. *J Atten Disord.* 1996;1(1): 16-30.

*Reason for exclusion: Co-intervention No safety data*

1. **Hinshaw1989a**

- Hinshaw SP, Henker B, Whalen CK, Erhardt D, Dunnington RE, Jr. Aggressive, prosocial, and nonsocial behavior in hyperactive boys: dose effects of methylphenidate in naturalistic settings. *J Consult Clin Psychol.* 1989;57(5):636-643.

*Reason for exclusion: Co-treatment Cross-over without wash out.*

1. **ISRCTN52376787**

- http://isrctn.com/ISRCTN52376787

*Reasons for exclusion: Methylphenidate vs. no methylphenidate No safety data provided*

1. **James2001**

- James RS, Walter JM, Sharp WS, Castellanos FX. Comparative efficacy of 3 amphetamine preparations in children with adhd. *39th Annual Meeting of the American College of* Neuropsychopharmacology*. 2000; Dec 10-14; San Juan, Puerto Rico.* 2000.
- James RS, Sharp WS, Bastain TM, et al. Double-blind, placebo-controlled study of single-dose amphetamine formulations in ADHD. *J Am Acad Child Adolesc Psychiatry.* 2001;40(11):1268-1276.

*Reason for exclusion: Co-treatment (behavior management techniques used during the programme) No number of headache cases (n) provided*

1. **Klein1997b**

- Klein RG, Abikoff H. Behavior therapy and methylphenidate in the treatment of children with ADHD. *J Atten Disord.* 1997;2(2):89-114.

*Reason for exclusion: No arms of interest for the present meta-analysis (Stimulants, parent and teacher training, stimulants+ parent and teacher training) No safety data*

1. **Konstenius2010**

- Konstenius M, Jayaram-Lindstrom N, Beck O, Franck J. Sustained release methylphenidate for the treatment of ADHD in amphetamine abusers: a pilot study. *Drug Alcohol Depend.* 2010;108(1-2):130-133.
- Konstenius M, Jayaram N, Guterstam J, Franck J. Pharmacological treatment of ADHD with amphetamine dependence. *Acta Neuropsychiatr.* 2013;25(S1): 13-14.

*Reason for exclusion: Co-treatment: skills training programme Adult participants*

1. **Kratochvil2011 (NCT00254462; K23MH066127)**

- Kratochvil CJ, Vaughan BS, Stoner JA, et al. A double-blind, placebo-controlled study of atomoxetine in young children with ADHD. *Pediatrics.* 2011;127(4):e862-868.
- https://clinicaltrials.gov/ct2/show/NCT00254462

*Reason for exclusion: Co-treatment with behavioral strategies No number of headache cases (n) provided*

1. **Levin1998**

- Levin FR, Evans SM, McDowell DM, Kleber HD. Methylphenidate treatment for cocaine abusers with adult attention-deficit/hyperactivity disorder: a pilot study. *J Clin Psychiatry.* 1998;59(6):300-305.
- Levin FR, Evans SM, Kleber HD. Methylphenidate Treatment for Cocaine Abusers with Adult Attention-Defict/Hyperactivity Disorder. *NIDA Res Monogr.* 1999:39.

*Reason for exclusion: Co-treatment with psychotherapy Adult participants*

1. **Levin2001**

- Levin ED, Conners CK, Silva D, Canu W, March J. Effects of chronic nicotine and methylphenidate in adults with attention deficit/hyperactivity disorder. *Exp Clin Psychopharmacol.* 2001;9(1):83-90.

*Reason for exclusion: No outcomes and no arms of interest for the present meta-analysis (placebo patch + placebo pill (control), nicotine patch + placebo pill (nicotine), placebo patch + methylphenidate pill (methylphenidate), and nicotine patch + methylphenidate pill (nicotine + methylphenidate) Adult participants*

1. **Levin2006 (NCT00061087)**

- Levin FR, Evans SM, Brooks D, Sullivan M, Nunes E, Vosburg S. Treatment of adult ADHD in methadone maintenance patients: Preliminary findings from a double-blind, three-armed, placebo-controlled trial. *Drug Alcohol* *Depend.* 2002;66:S102.
- Levin FR, Evans SM, Brooks DJ, Kalbag AS, Garawi F, Nunes EV. Treatment of methadone-maintained patients with adult ADHD: double-blind comparison of methylphenidate, bupropion and placebo. *Drug Alcohol Depend.* 2006;81(2):137-148.
- https://clinicaltrials.gov/ct2/show/NCT00061087

*Reason for exclusion*: *Co-treatment Adult participants*

1. **Levin2007 (NCT00136734)**

- Levin FR, Evans SM, Brooks DJ, Garawi F. Treatment of cocaine dependent treatment seekers with adult ADHD: double-blind comparison of methylphenidate and placebo. *Drug Alcohol Depend.* 2007;87(1):20-29.
- https://clinicaltrials.gov/ct2/show/NCT00136734

*Reason for exclusion: Co-treatment with cognitive therapy Adult participants*

1. **Levin2015 (NCT00553319)**

- Levin FR, Mariani JJ, Specker S, et al. Extended-Release Mixed Amphetamine Salts vs Placebo for Comorbid Adult Attention-Deficit/Hyperactivity Disorder and Cocaine Use Disorder: A Randomized Clinical Trial. *JAMA Psychiatry*. 2015;72(6):593-602.
- Levin FR, Mariani JJ, Mahony A, et al. Mixed amphetamine salts-extended release for ADHD adults with cocaine use disorder. *Drug Alcohol Depend.* 2015;146:e175.
- Notzon D, Mariani JJ, Pavlicova M, et al. Mixed-amphetamine salts increase abstinence from marijuana in patients with co-occurring attention-deficit/hyperactivity disorder and cocaine dependence. Drug *Alcohol Depend.* 2015;156:e164.
- Notzon DP, Mariani JJ, Pavlicova M, Glass A, Mahony AL, Brooks DJ, Grabowski J, Levin FR. Mixedamphetamine salts increase abstinence from marijuana in patients with co-occurring attention-deficit/hyperactivity disorder and cocaine dependence. *Am J Addict*. 2016;25(8):666-672
- https://clinicaltrials.gov/ct2/show/NCT00553319

*Reason for exclusion: Co-treatment with cognitive therapy Adult participants*

1. **Levin2018**

- Levin FR, Choi CJ, Pavlicova M, Mariani JJ, Mahony A, Brooks DJ, Nunes EV, Grabowski J. How treatment improvement in ADHD and cocaine dependence are related to one another: A secondary analysis. Drug Alcohol Depend. 2018;188:135-140.

*Reason for exclusion: Co-treatment with cognitive therapy Adult participants*

1. **Marchant2011 (NCT00506285; SLI381-404)**

- Marchant BK, Reimherr FW, Robison RJ, Olsen JL, Kondo DG. Methylphenidate transdermal system in ADHD adhd and impact on emotional and oppositional symptoms. *J Atten Disord.* 2011;15(4):295-304.
- Olsen JL, Reimherr FW, Marchant BK, Wender PH, Robison RJ. The effect of personality disorder symptoms on response to treatment with methylphenidate transdermal system in adults with attention-deficit/hyperactivity disorder. *Prim Care Companion CNS Disord.* 2012;14(5).
- Reimherr FW, Marchant BK, Olsen JL, Wender PH, Robison RJ. Oppositional defiant disorder in adults with ADHD. *J Atten Disord.* 2013;17(2):102-113.
- Gift TE, Reimherr FW, Marchant BK, Steans TA, Wender PH. Personality Disorder in Adult Attention-Deficit/Hyperactivity Disorder: Attrition and Change During Long-term Treatment. *J Nerv Ment Dis.* 2016;204(5):355-63.
- https://clinicaltrials.gov/ct2/show/NCT00506285

*Reason for exclusion: No oral formulations Adult participants*

1. **McGough2006b (NCT00466791)**

- McGough JJ, Wigal SB, Abikoff H, Turnbow JM, Posner K, Moon E. A randomized, double-blind, placebocontrolled, laboratory classroom assessment of methylphenidate transdermal system in children with ADHD. *J Atten* *Disord*.2006;9(3):476-485.
- Wigal S, Turnbow J, Abikoff H, McGough J, Cohen J. Parent rated effects of transdermal methylphenidate in children with ADHD. *Int J Neuropsychopharmacol.* 2008;11(Suppl. 1):232.
- https://clinicaltrials.gov/ct2/show/NCT00466791

*Reason for exclusion: No oral formulations participants were responders*

1. **NCT00736255 (SPD489-607)**

- https://clinicaltrials.gov/ct2/show/NCT00736255

*Reason for exclusion: Combined treatment Adult participants*

1. **NCT01711021**

- https://clinicaltrials.gov/ct2/show/NCT01711021

*Reason for exclusion: No formulation of interest for the present meta-analysis (transdermal) No safety data available*

1. **NCT00218322**

- https://clinicaltrials.gov/ct2/show/NCT00218322

*Reason for exclusion: Co-treatment with CBT participants including children and adults*

1. **NCT00261872**

- https://clinicaltrials.gov/ct2/show/NCT00261872

*Reason for exclusion: Co-treatment (behavioural therapy); refers to COMBINE Study Research Group. Testing combined pharmacotherapies and behavioural interventions for alcohol dependence (the COMBINE study): a pilot feasibility study. Alcohol Clin Exp Res. 2003 Jul; 27(7):1123-31. Adult participants*

1. **NCT00514202**

- https://clinicaltrials.gov/ct2/show/NCT00514202

*Reason for exclusion: Concomitant CBT Adult participants*

1. **Pelham2001a (NCT00269789)**

- Pelham WE, Hoffman MT, Lock T. Evaluation of once-a-day OROS methylphenidate HCI (MPH)extended-release tablets versus MPH tid in children with ADHD in natural school settings. *Pediatr Res*. 2000(4):31a.
- Pelham WE, Gnagy EM, Burrows-Maclean L, et al. Once-a-day Concerta methylphenidate versus three-times-daily methylphenidate in laboratory and natural settings. Pediatrics. 2001;107(6):E105.
- Williams L. Methylphenidate HCI extended-release tablets for children with ADHD: parent treatment prefence and satisfaction. Pediatr Res. 2001:429a.
- Swanson J. Impact of an OROS formulation of mwethylkphenidate on activity levels odf children with ADHD. Pediatr Res. 2001:429a.
- Commentary in: Connor. Once a day Concerta methylphenidate was equivalent to 3 times daily methylphenidate in children with ADHD. Evid Based Ment Health. 2002, 5, 20.
- Pooled in: Palumbo D, Spencer T, Lynch J, Co-Chien H, Faraone SV. Emergence of tics in children with ADHD: impact of once-daily OROS methylphenidate therapy. J Child Adolesc *Psychopharmacol*. 2004;14(2):185-194.

*Reason for exclusion: Co-treatment with behavioral intervention. Participants stabilized with MPH*

1. **Pelham2005**

- Pelham WE, Burrows-Maclean L, Gnagy EM, et al. Transdermal methylphenidate, behavioral, and combined treatment for children with ADHD. Exp Clin Psychopharmacol. 2005;13(2):111-126.
- Pelham WE, Jr., Manos MJ, Ezzell CE, et al. A dose-ranging study of a methylphenidate transdermal system in children with ADHD. J Am Acad Child Adolesc Psychiatry. 2005;44(6):522-529.

*Reason for exclusion: Transdermal formulation, no oral formulations Each treatment was administered for 1 day*

1. **Pelham2011**

- Pelham WE, Jr., Waschbusch DA, Hoza B, et al. Music and video as distractors for boys with ADHD in the classroom: comparison with controls, individual differences, and medication effects. *J Abnorm Child Psychol.* 2011;39(8):1085-1098.

*Reason for exclusion: Co-treatment (behavioral intervention) No usable safety data*

1. **Pelham2016**

- Pelham WE, Fabiano GA, Waxmonsky JG, et al. Treatment Sequencing for Childhood ADHD: A Multiple- Randomization Study of Adaptive Medication and Behavioral Interventions. *J Clin Child Adolesc* *Psychol.* 2016;45(4):396-415.

*Reason for exclusion: No design of interest for the present meta-analysis medication vs behavioral parent training*

1. **Perez-Alvarez2009**

- Perez-Alvarez F, Serra-Amaya C, Timoneda-Gallart CA. Cognitive versus behavioral ADHD phenotype: what is it all about? *Neuropediatrics.* 2009;40(1):32-38.

*Reason for exclusion: No arms of interest for the present meta-analysis (medication vs. psychological treatment vs combined) No safety data*

1. **Schachar1997**

- Schachar RJ, Tannock R, Cunningham C, Corkum PV. Behavioral, situational, and temporal effects of treatment of ADHD with methylphenidate. J Am Acad Child Adolesc Psychiatry. 1997;36(6):754-763.
- Diamond IR, Tannock R, Schachar RJ. Response to methylphenidate in children with ADHD and comorbid anxiety. J Am Acad Child Adolesc Psychiatry. 1999;38(4):402-409.
- Law SF, Schachar RJ. Do typical clinical doses of methylphenidate cause tics in children treated for attention-deficit hyperactivity disorder? J Am Acad Child Adolesc Psychiatry. 1999;38(8):944-951.
- Summarized in: Killeen MR. Do typical clinical doses of methylphenidate cause tics in children treated for attention-deficit hyperactivity disorder? J Child Fam Nurs. 2000;3(1):46-48.
- Follow up in: Charach A, Ickowicz A, Schachar R. Stimulant treatment over five years: adherence, effectiveness, and adverse effects. J Am Acad Child Adolesc Psychiatry. 2004;43(5):559-567.
- Charach A, Figueroa M, Chen S, Ickowicz A, Schachar R. Stimulant treatment over 5 years: effects on growth. J Am Acad Child Adolesc Psychiatry. 2006;45(4):415-421.

*Reason for exclusion: Co-intervention: parent training No number of headache cases (n) provided*

1. **Schubiner2002**

- Downey KK, Sclrubiner H, Schuster CR. Double-blind placebo controlled stimulant trial for cocaine dependent adhd adults. NIDA Res Monogr. 2000:116.
- Schubiner H, Saules KK, Arfken CL, et al. Double-blind placebo-controlled trial of methylphenidate in the treatment of adult ADHD patients with comorbid cocaine dependence. Exp Clin Psychopharmacol. 2002;10(3):286-294.

*Reason for exclusion: Co-intervention (CBT) Adult participants*

1. **Thurstone2010 (NCT00399763)**

- Thurstone C, Riggs PD, Salomonsen-Sautel S, Mikulich-Gilbertson SK. Randomized, controlled trial of atomoxetine for attention-deficit/hyperactivity disorder in adolescents with substance use disorder. *J Am Acad Child* *Adolesc Psychiatry.* 2010;49(6):573-582.
- Thurstone C, Salomensen-Sautel S, Riggs PD. How adolescents with substance use disorder spend research payments. *Drug Alcohol Depend.* 2010;111(3):262-264.
- https://clinicaltrials.gov/ct2/show/NCT00399763

*Reason for exclusion: Co-treatment (CBT) No number of headache cases (n) provided*

1. **Tucker2009**

- Tucker JD, Suter W, Petibone DM, et al. Cytogenetic assessment of methylphenidate treatment in pediatric patients treated for attention deficit hyperactivity disorder. *Mutat Res.* 2009;677(1-2):53-58.
- Zhou Y, Muni R, Tucker JD, Kumar V. Extendedrelease methylphenidate exposure and the frequency of cytogenetic abnormalities in children with attention-deficithyperactivity disorder. *J Child Adolesc* *Psychopharmacol. Proceedings of the 49th Annual National Institute of Mental Health (NIMH) New Clinical Drug* *Evaluation Unit (NCDEU) Meeting; 2009 June 29- July 2;Hollywood, Florida 2009;19(6):785.*

*Reason for exclusion: Co-intervention (behavioural therapy) No safety data of headache*

1. **Weiss2006a**

- Weiss M, Hechtman L. A randomized double-blind trial of paroxetine and/or Dextroamphetamine and problemfocused therapy for attention- deficit/hyperactivity disorder in adults. *J Clin Psychiatry* 2006, 67(4):611-619
- Weiss MD, Wasdell M, Gadow KD, Greenfield B, Hechtman L, Gibbins C. Clinical correlates of oppositional defiant disorder and attention-deficit/hyperactivity disorder in adults. *Postgrad Med.* 2011;123(2):177-184.
- Secondary analysis in: Weiss M, Murray C, Wasdell M, Greenfield B, Giles L, Hechtman L. A randomized controlled trial of CBT therapy for adults with ADHD with and without medication. *BMC Psychiatry.* 2012;12:30.

*Reason for exclusion: Co-treatment: psychotherapy. Adult participants*

1. **Wender1990**

- Wender PH, Reimherr FW. Bupropion treatment of attention-deficit hyperactivity disorder in adults. *Am J Psychiatry.* 1990;147(8):1018-1020.

*Reason for exclusion: Open label Adult participants*

1. **Whalen1989**

- Whalen CK, Henker B, Buhrmester D, Hinshaw SP, Huber A, Laski K. Does stimulant medication improve the peer status of hyperactive children? *J Consult Clin Psychol.* 1989;57(4):545-549.

*Reason for exclusion: Co-intervention (group CBT) No safety data*

1. **Wilens2008a (NCT00151970)**

- Frazier TW, Weiss M, Hodgkins P, Manos MJ, Landgraf JM, Gibbins C. Time course and predictors of healthrelated quality of life improvement and medication satisfaction in children diagnosed with attentiondeficit/

hyperactivity disorder treated with the methylphenidate transdermal system. *J Child Adolesc*

*Psychopharmacol.* 2010;20(5):355-364.

- López FA, Wilens TE, Wigal SB, Turnbow JM. Effects of variable wear times on transdermalmethylphenidate in attention-deficit/hyperactivity disorder. *Eur Neuropsychopharmacol.* Papers of the 21st ECNP Congress; 2008 August 30 - September 3; Barcelona, Spain. 2008; Vol. 18:S561–2.
- López FA, Landgraf JM, Wilens TE. Quality of life and parent satisfaction with the methylphenidate transdermal system. European Neuropsychopharmacology. *Papers of the 21st ECNP Congress; 2008 August 30 - September 3;* *Barcelona, Spain. 2008; 4: S562*
- Manos M, Frazier TW, Landgraf JM, Weiss M, Hodgkins P. HRQL and medication satisfaction in children with ADHD treated with the methylphenidate transdermal system. *Curr Med Res Opin.* 2009;25(12):3001-3010.
- Wilens TE, Boellner SW, Lopez FA, et al. Varying the wear time of the methylphenidate transdermal system in children with attention-deficit/hyperactivity disorder. *J Am Acad Child Adolesc Psychiatry.* 2008;47(6):700-708.
- https://clinicaltrials.gov/ct2/show/NCT00151970

*Reason for exclusion: Transdermal formulation No number of headache cases (n) of both treatment and placebo arm provided*

1. **Wilens2010 (NCT00586157)**

- Wilens T, Hammerness P, Utzinger L, Georgiopoulos A, Doyle R, Brodziak K, et al. Before-school ADHD symptoms and functioning in youth treated with the Methylphenidate Transdermal Patch (MTS). J Child Adolesc Psychopharmacol. Abstracts of the 49th Annual National Institute of Mental Health (NIMH)New Clinical Drug Evaluation Unit (NCDEU) Meeting;2009; June 29 - July 2; Hollywood, Florida. 2009; Vol. 19, 6:785–6.
- Wilens TE, Hammerness P, Martelon M, Brodziak K, Utzinger L, Wong P. A controlled trial of the methylphenidate transdermal system on before-school functioning in children with attention-deficit/hyperactivity disorder. *J Clin* *Psychiatry.* 2010;71(5):548-556.
- https://clinicaltrials.gov/ct2/show/NCT00586157

*Reason for exclusion: Transdermal formulation Cross-over without wash out.*

1. **Zhang2020**

- Zhang F, Zhu P, Wu L-H. Association of microRNA expression before and after drug therapy with clinical symptoms in children with attention deficit hyperactivity disorder. Chinese Journal of Contemporary Pediatrics. 2020;22(2):152-157.

*Reason for exclusion: Authors contacted to check eligibility but no response. No placebo arm; this article was written in Chinese*

**eTable 2.** Summary of characteristics of included clinical trials (*n* = 58)

| Article, study ID | Country | Study design, treatment period | Participants safety analysis (male%)^a^ | Age (y) | Comorbidity | Other remarks | Medication,  dosage | Diagnostic assessment | | Odds ratio  (95% CI) | | RoB2 | | | | | | |
| --- | --- | --- | --- | --- | --- | --- | --- | --- | --- | --- | --- | --- | --- | --- | --- | --- | --- | --- |
|  |  |  |  |  |  |  |  | ADHD | Headache |  |  | D1 | D2 | | D3 | D4 | D5 | Overall |
| Allen (2005),  B4Z-MC-LYAS | USA | Double blind, parallel, 18w, | Treatment: n = 76 (96.1)  Placebo: n = 72 (84.7) | 7-17 | Tics, ODD, MDD, GAD, OCD | All comorbid with tics | Atomoxetine,  0.5-1.5 mg/kg,  mean 1.33 mg/kg | DSM-IV | NR | 1.10  (0.49-2.47) | | L | L | | L | L | L | L |
| Bangs (2007),  B4Z-MC-LYAX, | USA | Double blind, parallel, 9w | Treatment: n = 72 (72.2)  Placebo: n = 70 (74.3) | 12-18 | MDD | All comorbid with MDD | Atomoxetine,  1.2-1.8 mg/kg,  mean 1.51mg/kg | DSM-IV | NR | 1.80  (0.66-4.88) | | L | L | | L | L | L | L |
| Bangs (2008),  B4Z-MC-LYAX,  NCT00191698 | Europe, Australia | Double blind, parallel, 8w | Treatment: n = 156 (91.7)  Placebo: n = 70 (97.1) | 6-12 | ODD | All comorbid with ODD | Atomoxetine,  1.2 mg/kg | DSM-IV | Open-ended question | 1.53  (0.82-2.87) | | L | L | | L | L | L | L |
| Biederman (2002),  SLI 381-301 | USA | Double blind, parallel, 3w | Treatment: n = 374 (79.4)  Placebo: n = 210 (72.9) | 6-12 | not specified |  | Amphetamine,  10, 20, 30 mg (3 arms) | DSM-IV | NR | 0.80  (0.52-1.22) | | L | L | | L | L | L | L |
| Biederman (2005), Study 311 Cephalon | USA | Double blind, parallel, 9w | Treatment: n = 164 (68.9)  Placebo: n = 82 (74.4) | 6-17 | none |  | Modafinil, 170-425 mg, mean 368.5 mg | DSM-IV | Spontaneously reported | 1.41  (0.69-2.92) | | L | | L | L | L | L | L |
| Biederman (2006) | USA | Double blind, parallel, 4w | Treatment: n = 197 (74.6)  Placebo: n = 51 (74.5) | 6-13 | none |  | Modafinil,  3 arms with 300 mg, 1 arm with 400 mg | DSM-IV | Spontaneously reported | 0.55  (0.25-1.21) | | S | | L | L | L | L | S |
| Biederman (2007), NRP104-301, NCT00248092 | USA | Double blind, parallel, 4w | Treatment: n = 218 (69.3)  Placebo: n = 72 (69.4) | 6-12 | none |  | Amphetamine,  30, 50, 70 mg (3 arms) | DSM-IV-TR | Open-ended question | 1.26  (0.52-3.03) | | L | | L | L | L | L | L |
| Biederman (2008),  SPD503-301, NCT00152009 | USA | Double blind, parallel, 8w | Treatment: n = 259 (74.5)  Placebo: n = 86 (74.4) | 6-17 | none |  | Guanfacine,  2, 3, 4 mg (3 arms) | DSM-IV-TR | NR | 1.10  (0.63-1.94) | | L | | L | L | L | L | L |
| Block (2009),  B4Z-US-LYCC, NCT00486122, | USA | Double blind, parallel, 6w | Treatment: n = 195 (71.8)  Placebo: n = 92 (74.2) | 6-12 | ODD |  | Atomoxetine,  0.47-1.81 mg/kg,  mean 1.25 and 1.26 mg/kg (AM and PM) | DSM-IV-TR | Open-ended question | 2.13  (0.69-6.50) | | L | | L | L | L | L | L |
| Brams (2018), NCT02466425 | USA | Double blind, parallel, 4w | Treatment: n = 132 (65.2)  Placebo: n = 131 (58.8) | 6-17 |  |  | Amphetamine,  12.5 or 25 mg | DSM-IV-TR | NR | 1.15  (0.54-2.47) | | S | | L | L | L | L | S |
| Childress (2009), CRIT124E2305, NCT00301236 | USA | Double blind, parallel, 5w | Treatment: n = 182 (63.8)  Placebo: n = 63 (66.2) | 6-12 | none |  | Methylphenidate,  10, 20, 30 mg (3 arms) | DSM-IV-TR | NR | 0.95  (0.40-2.25) | | L | | L | L | L | L | L |
| Coghill (2013), SPD489-325 | Europe  (10 countries) | Double blind, parallel, 7w | Treatment (A): n = 111 (78.4)  Treatment (M): n = 111 (81.1)  Placebo: n = 110 (82.7) | 6-17 | ODD, and other unspecified |  | Amphetamine,  30-70 mg,  mean 53.8 mg;  Methylphenidate,  18-54 mg,  mean 45.4 mg | DSM-IV-TR | NR | A: 0.67  (0.33-1.37)  M: 0.99  (0.51-1.91) | | L | | L | L | L | L | L |
| Connor (2010), SPD503-307, NCT00367835 | USA | Double blind, parallel, 9w | Treatment: n = 136 (64.0)  Placebo: n = 78 (76.9) | 6-12 |  |  | Guanfacine,  1-4 mg,  mean 2.9 mg | DSM-IV-TR | NR | 1.29  (0.64-2.62) | | L | | L | L | L | L | L |
| CRIT124DUS02 (2004) | USA | Double blind, crossover, 4w | Treatment: n = 102 (0)  Placebo: n = 101 (0) | 12-17 |  | All females | Methylphenidate,  20-60 mg | DSM-IV-TR | NR | 1.52  (0.76-3.04) | | S | | L | L | L | L | S |
| Daviss (2008), NCT00031395 | USA | Double blind, parallel, 16w | Treatment (C): n = 31 (87.1)  Treatment (M): n = 29 (82.8)  Placebo: n = 30 (76.7) | 7-12 | ODD, CD |  | Clonidine,  mean 0.24 mg;  Methylphenidate,  mean 30.2 mg | DSM-IV | Spontaneously reported | C: 1.73  (0.38-7.99)  M: 0.32  (0.03-3.28) | | L | | L | L | L | L | L |
| Dell’Agnello (2009) | Italy | Double blind, parallel, 8w | Treatment: n = 107 (93.3)  Placebo: n = 32 (90.6) | 6-15 | ODD, GAD, OCD, SAD, MDD, panic disorder, specific phobia, adjustment disorder, dysthymia | All comorbid with ODD | Atomoxetine,  0.85-1.33 mg/kg,  mean 1.10 mg/kg | DSM-IV | NR | 1.92  (0.61-6.02) | | S | | S | L | L | L | S |
| Dittmann (2011) | Germany | Double blind, parallel, 9w | Treatment: n = 121 (86.0)  Placebo: n = 59 (81.4) | 6-17 | ODD, CD, adjustment disorder | All comorbid with ODD or CD | Atomoxetine,  1.2 mg/kg | DSM-IV-TR | Open-ended question | 1.37  (0.59-3.18) | | L | | L | L | L | L | L |
| Findling (2010), NCT00499863, SPD485-40 | USA | Double blind, parallel, 7w | Treatment: n = 145 (75.2)  Placebo: n = 72 (73.6) | 13-17 |  | Transdermal | Methylphenidate,  10-30 mg,  mean 24.3 mg | DSM-IV-TR | Spontaneously reported | 0.99  (0.42-2.33) | | L | | S | L | S | L | S |
| Findling (2011),  SPD 489-305, NCT00735371 | USA | Double blind, parallel, 4w | Treatment: n = 233 (70.6)  Placebo: n = 77 (68.4) | 13-17 |  |  | Amphetamine,  30, 50, 70 mg (3 arms) | DSM-IV-TR | Spontaneously reported | 1.14  (0.54-2.44) | | L | | L | L | L | L | L |
| Froehlich (2018), NCT01727414 | USA | Double blind, crossover, 1w  (the first week data included) | Treatment: n = 108 (71.3)  Placebo: n = 53 (71.3) | 7-11 | ODD, CD, OCD, PTSD, MDD, SAD, GAD, dysthymia, social phobia, panic disorder | All stimulant-naïve | Methylphenidate,  18 mg | DSM-IV | Parent report questionnaire | 2.26  (0.96-5.35) | | S | | L | L | L | L | S |
| Gau (2007),  B4Z-TW-S010, NCT00485459 | Taiwan | Double blind, parallel, 6w | Treatment: n = 72 (90.3)  Placebo: n = 34 (85.3) | 6-16 | ODD, CD |  | Atomoxetine,  1.2-1.8 mg/kg, mean 1.4 mg/kg | DSM-IV | Open-ended question | 1.72  (0.34-8.77) | | L | | L | L | L | L | L |
| Geller (2007),  B4Z-US-LYBP | USA | Double blind, parallel, 10w | Treatment: n = 77 (62.1)  Placebo: n = 80 (67.4) | 8-17 |  | All comorbid with anxiety disorders^b^ | Atomoxetine,  1.2-1.8 mg/kg,  mean 1.43 mg/kg | DSM-IV | Open-ended question | 1.74  (0.64-4.75) | | L | | L | L | L | L | L |
| Greenhill (2002) | USA | Double blind, parallel, 3w | Treatment: n = 155 (82.6)  Placebo: n = 161 (81.1) | 6-16 |  |  | Methylphenidate,  20-60 mg,  mean 36.8 mg | DSM-IV | Participants: spontaneously reported (data used);  Parents and teachers:  questionnaire | Participants:  1.48  (0.76-2.88);  Parents:  1.23  (0.71-2.15) | | S | | L | L | L | L | S |
| Greenhill (2006a), Study 309 Cephalon | USA | Double blind, parallel, 9w | Treatment: n = 131 (72.5)  Placebo: n = 67 (73.1) | 6-17 |  |  | Modafinil,  170-425 mg,  mean 361.4 mg | DSM-IV | Spontaneously reported | 2.89  (1.14-7.36) | | L | | L | L | L | L | L |
| Greenhill (2006b), CRIT124E2301 | USA | Double blind, parallel, 7w | Treatment: n = 53 (58.5)  Placebo: n = 47 (70.0) | 6-17 |  | Classroom setting | Methylphenidate,  5-30 mg,  mean 24 mg | DSM-IV | Spontaneously reported | 2.73  (0.89-8.36) | | S | | L | L | L | L | S |
| Griffiths (2018), ACTRN 12607000535471 | Australia | Double blind, crossover, 6w | Treatment: n = 107 (78.4)  Placebo: n = 113 (78.4) | 6-17 | SAD, GAD, MDD, OCD, ODD, CD, PTSD, social anxiety, |  | Atomoxetine,  1.0-1.4 mg/kg,  mean 1.35 mg/kg | DSM-IV | Parent report questionnaire | 2.16  (0.39-12-02) | | L | | L | L | L | L | L |
| Harfterkamp (2012), NCT00380692 | the Netherlands | Double blind, parallel, 8w | Treatment: n = 48 (87.5)  Placebo: n = 49 (83.7) | 6-17 | ASD | All comorbid with ASD | Atomoxetine,  1.2 mg/kg | DSM-IV-TR | Open-ended question | 1.48  (0.56-3.93) | | L | | L | L | L | L | L |
| Hervas (2014), SPD503-316, NCT01244490, EudraCT: 2010- 018579-12 | Europe, USA, Canada | Double blind, parallel, 13w | Treatment (G): n = 114 (66.7)  Treatment (A): n = 112 (77.7)  Placebo: n = 111 (77.5) | 6-17 | ODD |  | Guanfacine,  1-7 mg,  mean 0.09 mg/kg;  Atomoxetine,  0.5-1.4 mg/kg,  mean 1.03 mg/kg | DSM-IV | NR | G: 1.11  (0.61-2.03)  A: 0.76  (0.40-1.44) | | L | | L | L | L | L | L |
| Ichikawa (2020), Japic CTI-152770 | Japan | Double blind, parallel, 4w | Treatment: n = 57 (82.5)  Placebo: n = 19 (84.2) | 6-17 |  |  | Amphetamine,  30, 50, 70 mg (3 arms) | DSM-5 | NR | no case in placebo arm | | S | | L | L | L | L | S |
| Kahbazi (2009) | Iran | Double blind, parallel, 6w | Treatment: n = 23 (78.3)  Placebo: n = 23 (73.9) | 6-15 |  |  | Modafinil,  200-300 mg | DSM-IV-TR | Checklist administered by a child  psychiatrist | 2.10  (0.18-24.87) | | L | | L | L | L | L | L |
| Kelsey (2004),  B4Z-US-LYBG | USA | Double blind, parallel, 8w | Treatment: n = 131 (70.7)  Placebo: n = 63 (70.3) | 6-12 | ODD, CD |  | Atomoxetine,  1.8 mg/kg | DSM-IV | Open-ended question | 0.44  (0.17-1.18) | | L | | L | L | L | L | L |
| Kollins (2011), SPD503-206, NCT00150592 | USA | Double blind, parallel, 6w | Treatment: n = 121 (66.1)  Placebo: n = 57 (77.2) | 6-17 |  | Laboratory classroom | Guanfacine,  1-3 mg,  mean 0.052 mg/kg | DSM-IV-TR | NR | 1.38  (0.63-3.00) | | L | | L | L | L | L | L |
| Martenyi (2010), B4Z-MW-LYCZ, NCT00386581 | Russia | Double blind, parallel, 6w | Treatment: n = 72 (87.5)  Placebo: n = 33 (81.8) | 6-16 |  |  | Atomoxetine,  1.2-1.8 mg/kg | DSM-IV | Open-ended question | 1.16  (0.21-6.30) | | L | | L | L | L | L | L |
| Mattingly (2020),  NCT03325881 | USA | Double blind, parallel, 4w | Treatment: n = 45 (67.4)  Placebo: n = 43 (60.0) | 6-12 |  |  | Methylphenidate,  6.25mg | DSM-5 | NR | 0.70  (0.15-3.31) | | L | | L | L | L | L | L |
| Michelson (2001), B4Z-MC-LYAC | USA | Double blind, parallel, 8w | Treatment: n = 211 (71.4)  Placebo: n = 83 (71.4) | 8-18 | ODD, GAD |  | Atomoxetine, 0.5, 1.2, 1.8 mg/kg (3 arms) | DSM-IV | Open-ended question | 1.07  (0.59-1.96) | | L | | L | L | L | L | L |
| Michelson (2002), B4Z-MC-LYAT | USA | Double blind, parallel, 6w | Treatment: n = 85 (70.6)  Placebo: n = 85 (70.6) | 6-16 | ODD, GAD, depression, specific phobia |  | Atomoxetine,  1.0-1.5 mg/kg | DSM-IV | Open-ended question | 1.17  (0.54-2.52) | | L | | L | L | L | L | L |
| Montoya (2009),  B4Z-XM-LYDM, NCT00191945 | Spain | Double blind, parallel, 12w | Treatment: n = 100 (79.0)  Placebo: n = 51 (80.4) | 6-15 | ODD, tics, anxiety disorder, affective disorder | All treatment-naïve | Atomoxetine,  0.8-1.4mg/kg,  mean 1.2 mg/kg | DSM-IV-TR | NR | 3.51  (0.98-12.55) | | L | | L | L | L | L | L |
| NCT00936299 (2019) | USA | Quadruple blind, parallel, 16w | Treatment: n = 53 (86.8)  Placebo: n = 52 (88.5) | 13-19 | SUD | All with SUD; add-on CBT | Bupropion,  300 mg | DSM-IV | NR | 0.79  (0.33-1.93) | | S | | S | L | L | L | S |
| Newcorn (2008), B4Z-MC-LYBI | USA | Double blind, parallel, 6w | Treatment (A): n = 221 (77.5)  Treatment (M): n = 219 (70.9)  Placebo: n = 74 (74.3) | 6-16 | ODD |  | Atomoxetine,  0.8-1.8 mg/kg,  mean 1.45 mg/kg;  Methylphenidate,  18-54 mg,  mean 39.9 mg | DSM-IV | Open-ended question | A: 2.05  (0.87-4.81)  M: 1.23  (0.51-2.98) | | L | | L | L | L | L | L |
| Newcorn (2013), SPD503-314, NCT00997984 | USA | Double blind, parallel, 8w | Treatment: n = 221 (67.9)  Placebo: n = 112 (75.9) | 6-12 |  |  | Guanfacine,  1-4 mg,  mean 0.084 mg/kg | DSM-IV-TR | Spontaneously reported | 1.68  (0.84-3.36) | | S | | H | L | H | L | H |
| Newcorn (2017)^c^, SPD489-405, NCT01552915 | USA | Double blind, parallel, 8w | Treatment (A): n = 184 (66.3)  Treatment (M): n = 184 (66.3)  Placebo: n = 91 (67.0) | 13-17 |  |  | Amphetamine,  30-70 mg,  mean 55.15 mg;  Methylphenidate,  18-72 mg,  mean 44.47 mg | DSM-IV-TR | NR | A: 2.15  (0.90-5.14)  M: 2.15  (0.90-5.14) | | L | | L | L | L | L | L |
| Newcorn (2017)^c^, SPD489-406, NCT01552902 | USA, Canada, Germany, Hungary, Sweden | Double blind, parallel, 6w | Treatment (A): n = 218 (68.5)  Treatment (M): n = 219 (61.9)  Placebo: n = 110 (69.1) | 13-17 |  |  | Amphetamine,  70 mg;  Methylphenidate,  72 mg | DSM-IV-TR | NR | A: 2.00  (0.92-4.35)  M: 2.13  (0.99-4.62) | | L | | L | L | L | L | L |
| Pliszka (2000) | USA | Double blind, parallel, 3w | Treatment (A): n = 20 (NR)  Treatment (M): n = 20 (NR)  Placebo: n = 18 (NR) | 6-12 | ODD, CD, anxiety disorder |  | Amphetamine,  10-30 mg,  mean 12.5 mg;  Methylphenidate,  10-50 mg,  mean 25.2 mg | DSM-IV | Parent report questionnaire | A: 1.89  (0.16-22.79)  M: no case | | L | | L | L | L | L | L |
| Pliszka (2017), NCT02520388 | USA | Double blind, parallel, 3w | Treatment: n = 81 (67.9)  Placebo: n = 80 (72.5) | 6-12 |  |  | Methylphenidate,  40-80 mg | DSM-5 | Spontaneously reported | 2.08  (0.60-7.21) | | S | | S | L | S | L | S |
| Riggs (2011), NCT00264797, | USA | Double blind, parallel, 16w | Treatment: n = 151 (80.8)  Placebo: n = 152 (77.0) | 13-18 | SUD, CD, MDD | All with SUD; add-on CBT | Methylphenidate,  72 mg | DSM-IV | NR | 1.19  (0.75-1.87) | | S | | L | L | L | L | S |
| Rugino (2003) | USA | Double blind, parallel, 6w | Treatment: n = 11 (63.6)  Placebo: n = 11 (63.6) | 5-15 | ODD, CD, SAD, specific phobia, enuresis |  | Modafinil,  200-300 mg,  mean 264 mg | DSM-IV | NR | no case in placebo arm | | L | | L | L | L | L | L |
| Rugino (2018), NCT01156051 | USA | Double blind, parallel, 5w | Treatment: n = 11 (81.8)  Placebo: n = 16 (50.0) | 6-12 |  |  | Guanfacine,  1-4 mg,  mean 3 mg | DSM-IV-TR | NR | 4  (0.58-27.41) | | S | | S | L | L | L | S |
| Sallee (2009), SPD503-304, NCT00150618 | USA | Double blind, parallel, 9w | Treatment: n = 256 (73.2)  Placebo: n = 66 (68.2) | 6-17 |  |  | Guanfacine,  1, 2, 3, 4 mg (4 arms) | DSM-IV-TR | NR | 2.2  (0.95-5.10) | | L | | L | L | L | L | L |
| Spencer (2002)^d^, B4Z-MC-HFBD, B4Z-MC-HFBK | USA | Double blind, parallel, 12w | Treatment: n = 129 (76.0)  Placebo: n = 124 (83.1) | 7-12 |  |  | Atomoxetine,  maximum 2 mg/kg | DSM-IV | Spontaneously reported | 1.10  (0.64-1.90) | | L | | L | L | L | L | L |
| Spencer (2006), SLI381-314, NCT00507065 | USA | Double blind, parallel, 4w | Treatment: n = 233 (65.0)  Placebo: n = 54 (67.3) | 13-17 |  |  | Amphetamine,  10, 20, 30, 40 mg (4 arms) | DSM-IV-TR | NR | 0.68  (0.33-1.41) | | L | | L | L | L | L | L |
| Svanborg (2009), B4Z-SO-LY15, EUCTR2004-003941-42-SE, NCT00191542 | Sweden | Double blind, parallel, 10w | Treatment: n = 49 (79.6)  Placebo: n = 50 (82.0) | 7-15 | Tics, ODD, depression | All stimulant-naïve | Atomoxetine,  0.6-1.4 mg/kg,  mean 1.1 mg/kg | DSM-IV | NR | 2.89  (1.15-7.26) | | L | | L | L | L | L | L |
| Swanson (2006) | USA | Double blind, parallel, 7w | Treatment: n = 125 (74.4)  Placebo: n = 64 (65.6) | 6-17 |  |  | Modafinil,  340 or 425 mg | DSM-IV-TR | Parent report questionnaire | 1.23  (0.53-2.88) | | S | | L | L | L | L | S |
| Takahashi (2009), B4Z-JE-LYBC, NCT00191295 | Japan | Double blind, parallel, 8w | Treatment: n = 183 (85.8)  Placebo: n = 62 (83.9) | 6-17 |  |  | Atomoxetine,  0.5, 1.2, 1.8 mg/kg (3 arms) | DSM-IV | NR | 0.93  (0.28-3.03) | L | | | L | L | L | L | L |
| Wehmeier (2012), B4Z-SB-LYDV, NCT00546910 | Germany | Double blind, parallel, 8w | Treatment: n = 63 (74.6)  Placebo: n = 62 (80.6) | 6-12 | ODD, CD, Tics, mood disorder |  | Atomoxetine,  1.2 mg/kg | DSM-IV-TR | Open-ended question | 0.57  (0.13-2.50) | L | | | L | L | L | L | L |
| Wigal (2004) | USA | Double blind, parallel, 4w | Treatment: n = 90 (90.0)  Placebo: n = 42 (83.3) | 6-17 |  |  | Methylphenidate,  mean 18.25 mg (d-MPH), 32.14 mg (d,l-MPH) | DSM-IV | NR | 2.21  (0.70-7.04) | S | | | L | L | L | L | S |
| Wigal (2015), NCT01239030, | USA | Double blind, parallel, 1w | Treatment: n = 183 (67.8)  Placebo: n = 47 (63.8) | 6-18 |  |  | Methylphenidate, 10, 15, 20, 40 mg (4 arms) | DSM-IV-TR | NR | 1.17  (0.38-3.61) | S | | | S | L | S | L | S |
| Wilens (2015), SPD503-312, EUCTR2011-002221-21, NCT01081132 | USA | Double blind, parallel, 13w | Treatment: n = 157 (65.6)  Placebo: n = 155 (63.9) | 13-17 | ODD |  | Guanfacine,  1-7 mg,  mean 4.3 mg | DSM-IV-TR | NR | 1.66  (0.96-2.84) | L | | | L | L | L | L | L |

NR, not reported; RoB 2, Revised Cochrane risk-of-bias tool for randomized trials; ASD, autism spectrum disorder; MDD, major depressive disorder; ODD, oppositional defiant disorder; GAD, generalized anxiety disorder; OCD, obsessive-compulsive disorder; CD, conduct disorder; SUD, substance use disorders; SAD, separation anxiety disorder; PTSD, post-traumatic stress disorder; CBT, cognitive-behavioral therapy;

^a^Sex distribution in the intention-to-treat sample

^b^Anxiety disorders: generalized anxiety disorder, separation anxiety disorder, and/or social phobia

^c^This article reported the results of two studies

^d^This article reported the pooled data of two studies

**eFigure 3.** Forest plot of risk of headache between amphetamine treatment groups and placebo groups


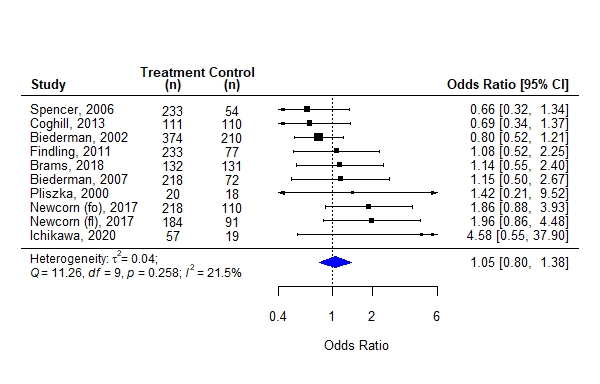


**eFigure 4.** Funnel plot of trials on the association between amphetamine and headache among children with ADHD


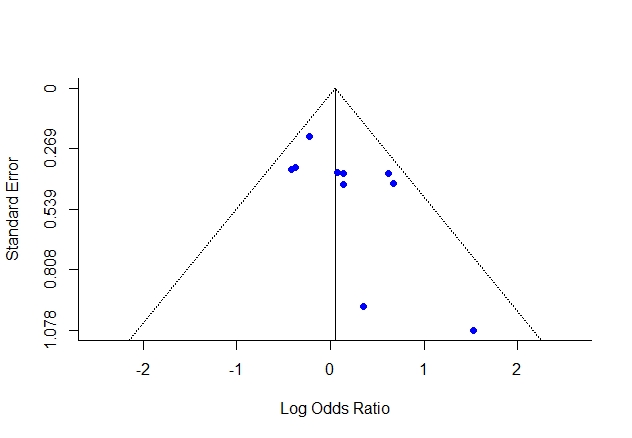


Egger's test: *t* = 2.10, *df* = 8, *p* = 0.069

**eFigure 5.** Forest plot of risk of headache between atomoxetine treatment groups and placebo groups, with subgroup analysis based on maximum dosage


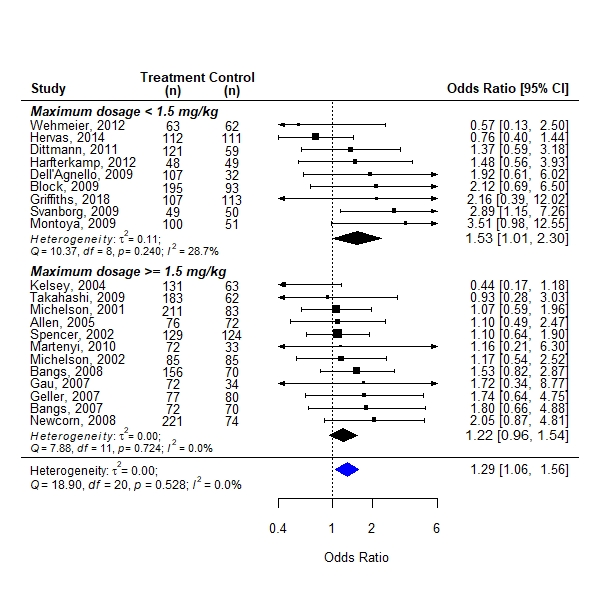


**eFigure 6.** Forest plot of risk of headache between atomoxetine treatment groups and placebo groups, with subgroup analysis based on dosing strategy


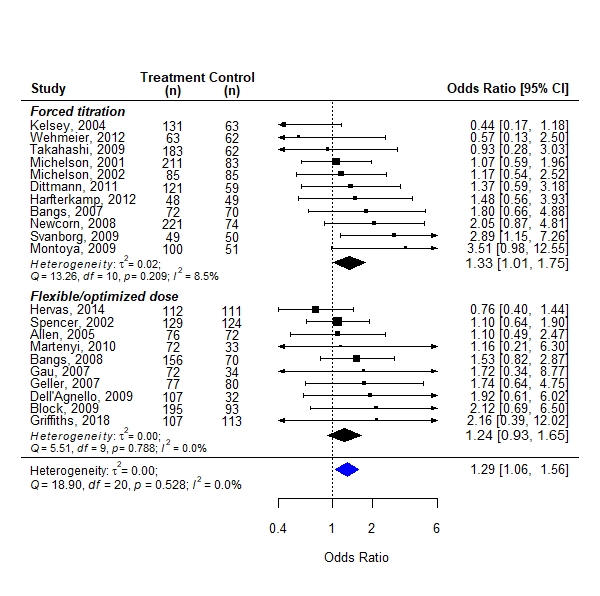


**eFigure 7.** Funnel plot of trials on the association between atomoxetine and headache among children with ADHD


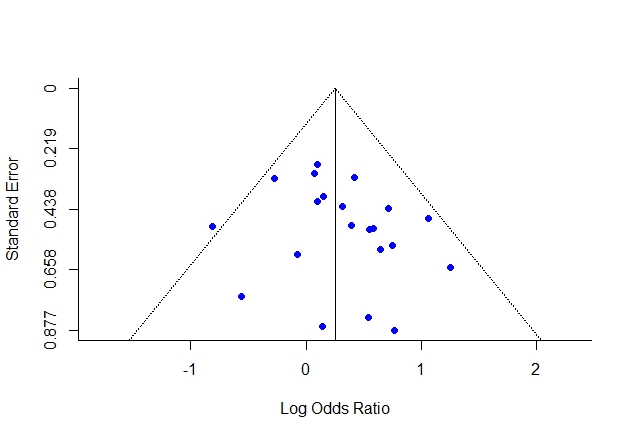


Egger's test: *t* = 1.27, *df* = 19, *p* = 0.221

**eFigure 8.** Forest plot of risk of headache between guanfacine treatment groups and placebo groups


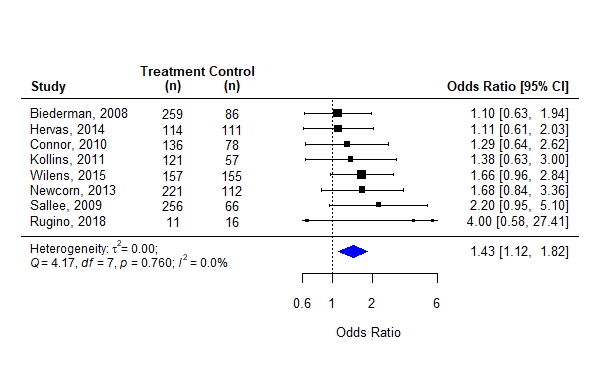


**eFigure 9.** Funnel plot of trials on the association between guanfacine and headache among children with ADHD


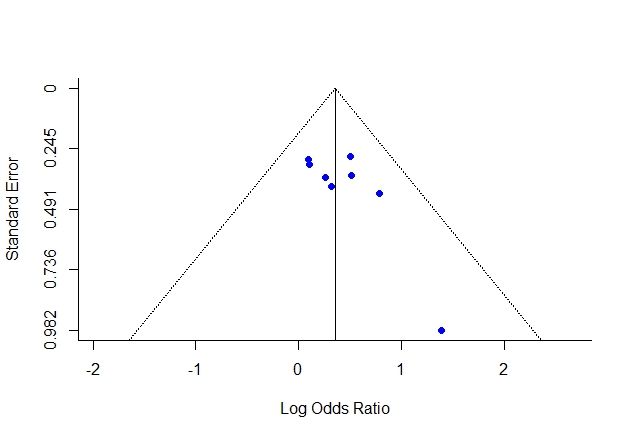


Egger's test: *t* = 2.05, *df* = 20, *p* = 0.086

**eFigure 10.** Forest plot of risk of headache between methylphenidate treatment groups and placebo groups


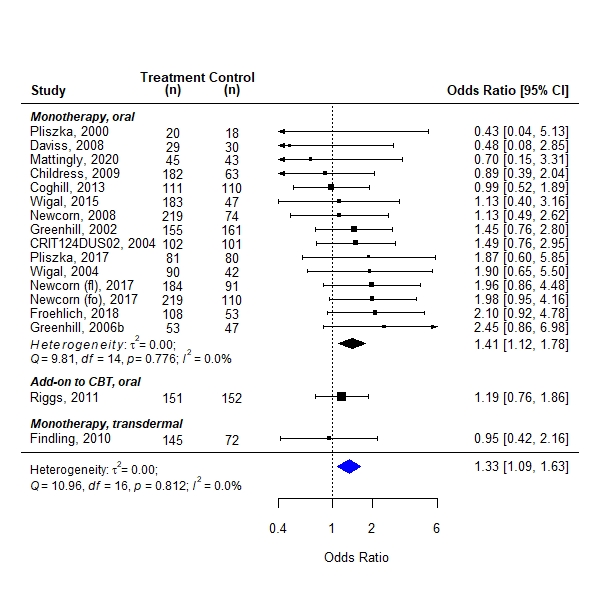


**eFigure 11**. Funnel plot of trials on the association between methylphenidate and headache among children with ADHD


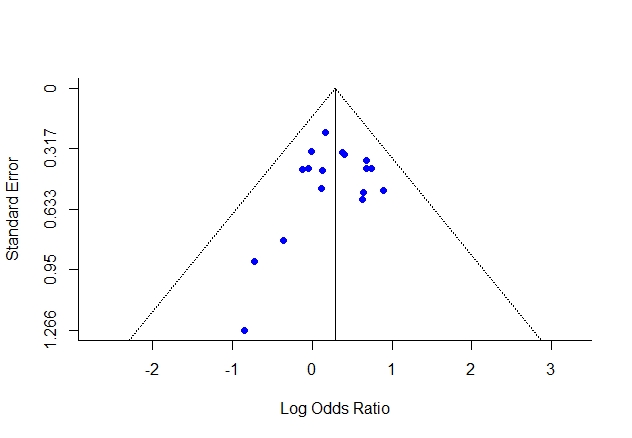


Egger's test: *t* = -0.58, *df* = 15, *p* = 0.570

**eFigure 12.** Forest plot of risk of headache between modafinil treatment groups and placebo groups


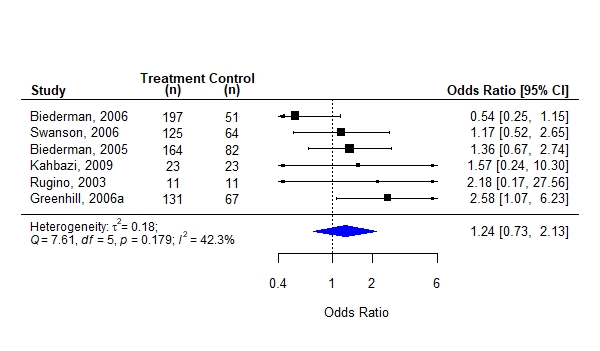


**eFigure 13.** Funnel plot of trials on the association between modafinil and headache among children with ADHD


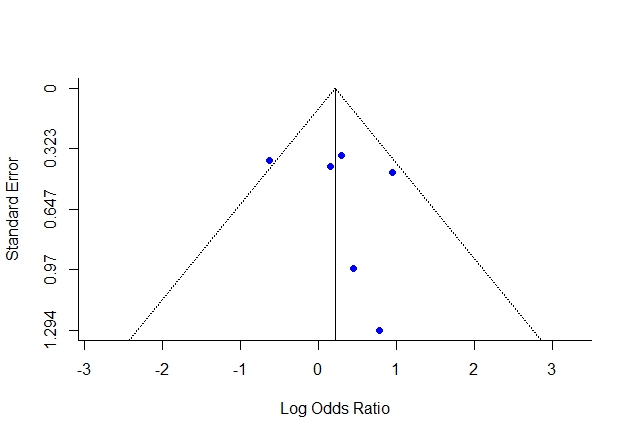


Egger's test: *t* = 0.59, *df* = 4, *p* = 0.589


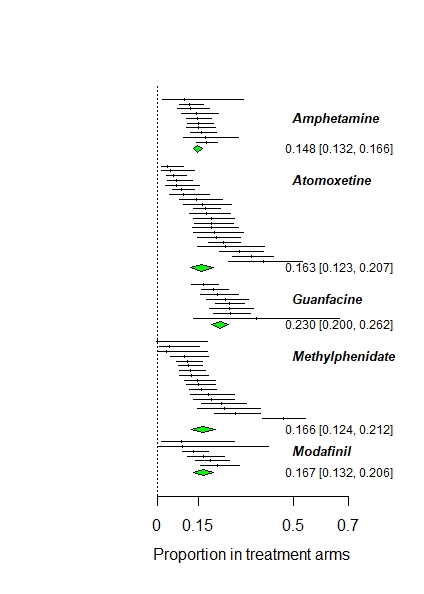
**eFigure 14.** Caterpillar plot of trials on the prevalence of headache among children with ADHD treated with ADHD medications

**eFigure 15.** Caterpillar plot of trials on the prevalence of headache among children with ADHD in placebo arms


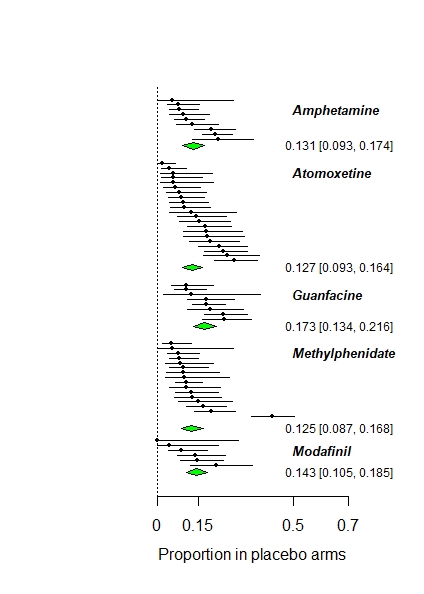


**eFigure 16.** Caterpillar plot of trials on the prevalence of headache among youths with ADHD in placebo arms based on different age


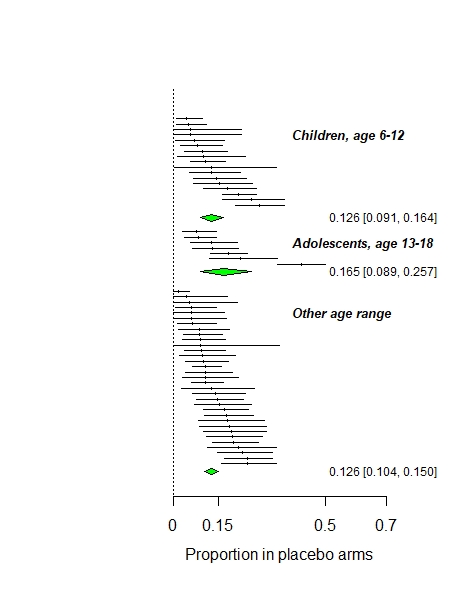

Supplement: Supplementary file 1 [file S0033291721004141sup001.docx]
